# Trends in the prevalence and burden of mental disorders among adolescents and young adults, 1990–2021

**DOI:** 10.3389/fpubh.2026.1767326

**Published:** 2026-02-19

**Authors:** Huiyuan Pang, Yixuan Lu, Xianxian Yuan, Ruihua Yang, Yujie Zhang, Xin Yan, Lirui Zhang, Junhua Huang, Wei Zheng, Guanghui Li

**Affiliations:** 1 Division of Endocrinology and Metabolism, Department of Obstetrics, Beijing Obstetrics and Gynecology Hospital, Capital Medical University, Beijing, China; 2 Beijing Maternal and Child Health Care Hospital, Beijing, China

**Keywords:** adolescents, COVID-19 impact, global burden of disease, mental disorders, young adults

## Abstract

**Background:**

Mental disorders significantly contribute to the global disease burden, often beginning in childhood and adolescence. This study evaluates trends and disease burdens of mental disorders in individuals aged 10 to 24 from 1990 to 2021.

**Methods:**

We utilized data from the Global Burden of Diseases, Injuries, and Risk Factors Study (GBD) 2021 to analyze age-standardized prevalence (ASP) and age-standardized disability-adjusted life years (DALYs) for 10 mental disorder categories. Trend analysis employed joinpoint regression and the age-period-cohort model to assess interactions among age, period, and cohort effects while decomposing DALY trends into population growth, aging, and morbidity changes.

**Results:**

The ASP of mental disorders rose from 13,824.9 (95% uncertainty intervals [UI]: 12,010.6–15,751.2) per 100,000 in 1990 to 14,764.9 (95% UI: 12,804.9–16,908.1) in 2021. Age-standardized DALYs increased from 1,680.3 (95% UI: 1,215.3–2,226.5) to 1,919.2 (95% UI: 1,370.5–2,564.1). Anxiety disorders had the highest ASP at 4,968.0 (95% UI: 3,639.4–6,612.3) and DALYs at 609.4 (95% UI: 377.5–909.1) in 2021. Significant increases in ASP and DALYs for anxiety and depressive disorders were noted in 2020 and 2021, particularly among females and across all socio-demographic index (SDI) regions, with higher SDI regions experiencing more rapid growth.

**Conclusion:**

This study reveals a concerning rise in mental disorders among adolescents and young adults, especially anxiety and depression, with notable accelerations observed in recent years. These trends highlight the urgent need for targeted mental health interventions for this vulnerable group.

## 1 Introduction

Mental disorders rank among the top 10 leading causes of disease-related burden worldwide, impacting millions of individuals globally (1, 2). Longitudinal studies tracking individuals from birth to adulthood reveal that the majority of adult mental health disorders originate during childhood and adolescence, which is a critical period for developing the social and emotional competencies essential for mental well-being (3). Recent developmental and public health frameworks have proposed an expanded definition of adolescence spanning ages 10–24 years, reflecting contemporary patterns of biological maturation, brain development, and delayed social role transitions (4, 5). Individuals aged 10–24 years account for approximately 24% of the global population, underscoring the substantial demographic importance of this age group (6). It is estimated that approximately one in seven individuals aged 10 to 19 are affected by mental health conditions worldwide (7). Alarmingly, suicide ranks as the second leading cause of death among individuals aged 15 to 24 years (8). The early onset of these disorders can hinder the transition to a healthy adulthood and reduce future productivity, leading to a range of negative outcomes, including increased rates of school dropout, diminished workforce participation, and elevated suicide risk (9–11). The landscape of mental health has been further complicated by the COVID-19 pandemic, which has significantly affected the well-being of young people. In 2021, over a third of high school students reported experiencing poor mental health during the pandemic (12).

Understanding the prevalence and burden of mental disorders during adolescence and young adulthood is essential for effective public health planning and service delivery. However, most existing studies have relied on cross-sectional or short-term data, offering limited insight into long-term temporal dynamics. In addition, few studies have systematically examined trends across socio-demographic development levels or disentangled the drivers underlying observed changes. Therefore, a comprehensive assessment of long-term trends from 1990 to 2021, stratified by sex and SDI regions and supported by complementary analytical approaches, is needed to better characterize the evolving burden of mental disorders in this population. Accordingly, this study uses data from the Global Burden of Diseases, Injuries, and Risk Factors Study (GBD) 2021 to assess trends and disease burden among adolescents and young adults.

## 2 Methods

### 2.1 Data source

This study utilized data from the GBD 2021, which provides estimates for the disease burden associated with 371 diseases and injuries, as well as 88 risk factors, disaggregated by age and sex for 21 regions, 204 countries and territories, covering the period from 1990 to 2021. For further details regarding the location, disease, and risk hierarchies utilized in the GBD 2021, please refer to the Global Health Data Exchange (GHDx).^1^

The GBD 2021 study synthesizes a comprehensive array of data sources to estimate mortality, causes of death, illness, and risk factors. This research specifically focuses on estimating the prevalence and burden associated with mental disorders among individuals aged 10 to 24 years worldwide from 1990 to 2021. The GBD 2021 dataset consists of 1,340 input sources and includes a total of 35,880 metadata entries related to mental disorders. After extracted data for individuals aged 10 to 24 years, 5,565 metadata entries remain. The analyses and reports presented in this study adhere to established guidelines for the accurate and transparent reporting of health estimates.

### 2.2 Definition of mental diseases

The mental disorders included in this study were extracted from the GBD 2021 cause hierarchy at levels 2 and 3. These disorders encompass total mental disorders and are further classified into anxiety disorders, attention-deficit/hyperactivity disorder (ADHD), autism spectrum disorders, bipolar disorder, conduct disorder, depressive disorders (including major depressive disorder and dysthymia), eating disorders (anorexia nervosa and bulimia nervosa), idiopathic developmental intellectual disability, schizophrenia, and a residual category for other mental disorders. Detailed definitions of these mental disorders are provided in the Supplementary methods, along with the corresponding cataloging system codes (ICD-10 and DSM-IV-TR).

### 2.3 Estimates of disease burdens

We selected prevalence and disability-adjusted life years (DALYs) data for mental disorders from GBD 2021 as the analysis metrics. Prevalence for mental disorders is defined as the number of cases within a population at a specific point in time. The age-standardized prevalence (ASP) was calculated by multiplying crude rates of 5-year age groups by their proportions in the standard population and summing the results.

DALYs are a measure of disease burden that quantify the total years of healthy life lost due to a disease, encompassing both years of life lost (YLLs) and years lived with disability (YLDs). In this study, YLLs associated with mental disorders are set to 0 due to the lack of direct attribution of related deaths in the GBD estimates, resulting in DALYs being equivalent to YLDs. Additionally, age-standardized DALYs were reported in this study.

The estimates were generated using DisMod-MR 2.1, a Bayesian mixed-effects meta-regression modeling tool specifically developed for GBD analyses. This modeling approach systematically adjusted epidemiological data to mitigate biases resulting from variations in data sources, definitions, and measurement methods.

### 2.4 Classifications of regions, countries and territories, and socio-demographic index

The GBD 2021 data were presented through multiple stratifications, including 21 GBD regions, 204 countries and territories, and classifications based on the SDI developed by the Institute for Health Metrics and Evaluation (IHME) in 2015. SDI serves as a comprehensive indicator of social development and its relationship to population health outcomes across countries and regions. The SDI is calculated as the geometric mean of three standardized indicators: total fertility rate for individuals under 25 years old, mean years of schooling for those aged 15 and older, and lag-distributed income per capita. For the GBD 2021 study, SDI classified into five SDI categories: low, low-middle, middle, high-middle, and high. A low SDI indicates low income and education levels with high fertility, while a high SDI signifies high income and educational attainment with low fertility.

### 2.5 Statistical methods

This study reports the ASP and age-standardized DALYs per 100,000 population, with 95% uncertainty intervals (UIs). Joinpoint regression was employed to identify turning points, known as joinpoints, in disease temporal trend changes and to calculate the annual percent change (APC) between these turning points, along with the overall average annual percent change (AAPC) (13). Initially, piecewise regression was performed using a logarithmic linear model (ln y = *β* × x). A grid search method was utilized to identify all potential joinpoints, calculating the mean squared errors (MSE) for each scenario and selecting the grid point with the smallest MSE as the joinpoint. To determine the optimal model for joinpoint regression, a Monte Carlo permutation test was conducted, with the maximum potential number of joinpoints set at 5. Based on the optimal model, the APC and AAPC were calculated to quantify trends from 1990 to 2021. The APC is computed as follows: APC = (e*^β^* - 1) × 100%, where β is the regression coefficient from the logarithmic linear model. The AAPC is calculated by weighting the APC values across segments according to their interval spans, representing the overall prevalence trend from 1990 to 2021.

In this study, we employed the age-period-cohort model to examine the complex interactions among age, period, and cohort, identifying their collective influence on the disease burden of mental disorders. The estimate of net drift represents the log-linear trend across periods and cohorts for the entire population, representing the overall APC adjusted for age group over time. In contrast, local drift indicates the annual percentage change specific to each age group. The longitudinal age curve illustrates the fitted age-specific rates in the reference cohort, adjusted for period effects. The period relative risk (RR) quantifies the RR for the population across different periods, adjusted for age and cohort effects, while the cohort RR assesses the RR across various cohorts, adjusted for age and period. For the age-period-cohort analysis, data were organized into consecutive 5-year age groups (10–14, 15–19, and 20–24 years) and 5-year calendar periods from 1990–1994 to 2015–2019. The remaining years (2020–2021) were combined into the final period. Birth cohorts were subsequently derived from the age-period structure following standard APC modeling conventions. We utilized the Age-Period-Cohort Web Tool developed by the Biostatistics Branch of the National Cancer Institute in Bethesda, MD, US, to derive the estimable parameters (14). The significance of these estimable parameters and functions was assessed using Wald chi-square tests.

We next decomposed trends in DALYs into three components—population growth, population aging, and morbidity change—using the method established by Cheng et al. (15). In this study, “population aging” refers to shifts in the internal age composition within the 10-24-year population, such as an increasing proportion of individuals aged 20–24 years relative to younger adolescents. This analysis evaluates how an aging population, overall population increases, and variations in health conditions contribute to DALYs. It also considers interactions among these components to capture their combined effects. By examining these factors and their interrelationships, we determined their relative contributions to changes in DALYs over time, with detailed formulas provided in the previous report (15).

## 3 Results

**Figure 1:**
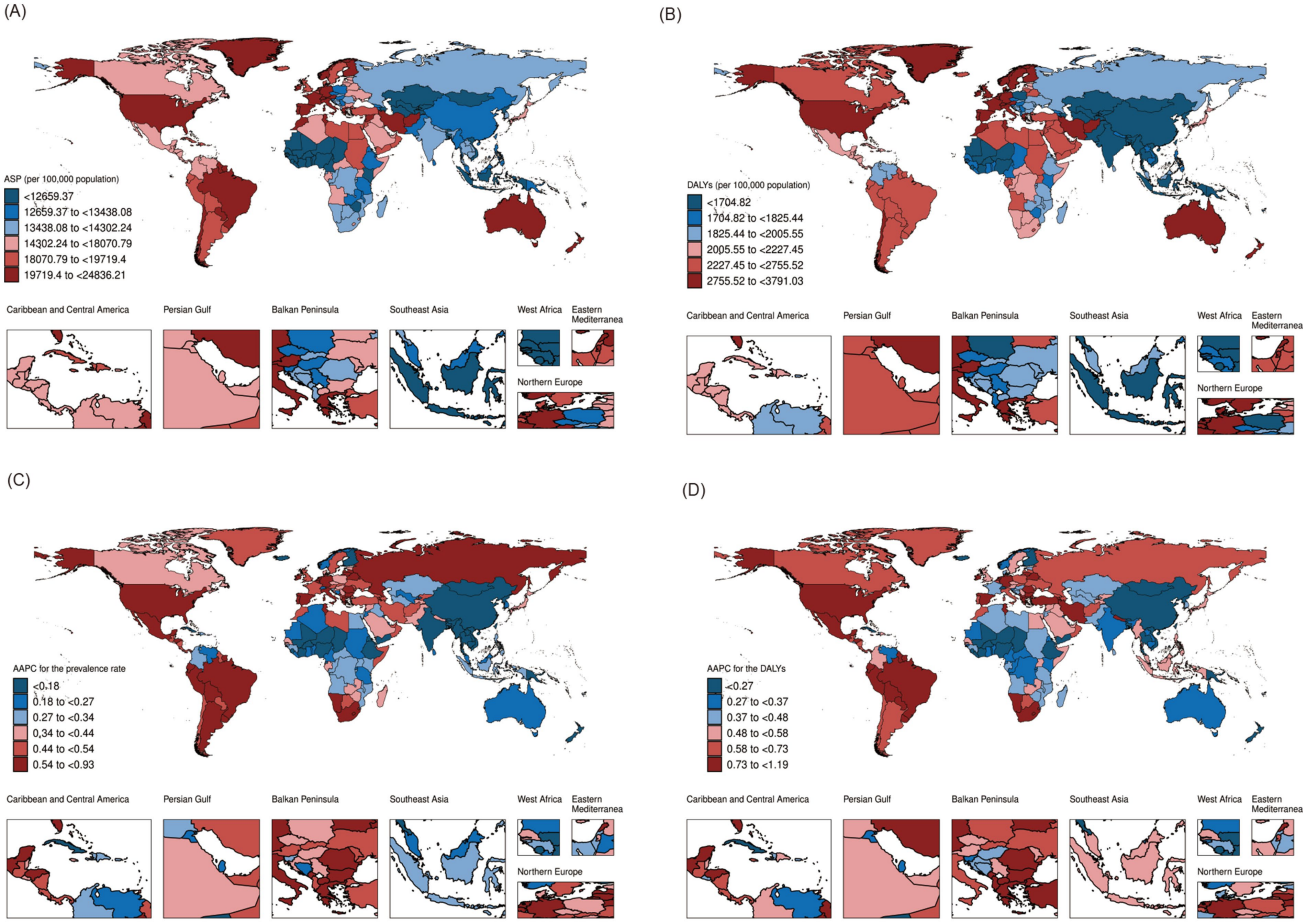
Global map of **(A)** age-standardized prevalence (ASP) and **(B)** age-standardized disability-adjusted life years (DALYs) for total mental disorders in individuals aged 10–24 in 2021, and average annual percent change in **(C)** ASP and **(D)** DALYs from 1990 to 2021 across 204 countries and territories.

The ASP of total mental disorders among adolescents and young adults per 100,000 population increased from 13,824.9 (95% UI: 12,010.6-15,751.2) in 1990 to 14,764.9 (95% UI: 12,804.9-16,908.1) in 2021. Similarly, the age-standardized DALYs per 100,000 population rose from 1,680.3 (95% UI: 1,215.3-2,226.5) to 1,919.2 (95% UI: 1,370.5-2,564.1) during the same period. Notably, the increase in ASP and age-standardized DALYs was more pronounced among females compared to males. Furthermore, an upward trend in both ASP and age-standardized DALYs was observed across all five SDI regions, with higher SDI regions experiencing more rapid growth. Among the 21 GBD regions, both ASP and age-standardized DALYs increased, except for East Asia and South Asia, where no increase in ASP was identified (Table 1; Figure 1). Among the 204 countries and territories analyzed, 195 exhibited an increase in ASP, while only 4 reported a decrease, and 5 showed no notable change. Regarding age-standardized DALYs, 201 countries and territories experienced a substantial increase, 2 reported a decline, and 1 remained unchanged (Supplementary Table S1; Figure 1).

**Table 1:** Global and regional age-standardized prevalence and DALYs of mental disorders in adolescents and young adults, 1990–2021.

Characteristics	Age-standardized-prevalence per 100,000 population			Age-standardized-DALYs per 100,000 population		
	1990(95% UI)	2021(95% UI)	AAPC (95% CI)	1990(95% UI)	2021(95% UI)	AAPC (95% CI)
Total	13824.9(12010.6–15751.2)	14764.9(12804.9–16908.1)	0.26(0.21–0.33)*	1680.3(1215.3–2226.5)	1919.2(1370.5–2564.1)	0.47(0.40–0.56)*
Male	14020.3(12141.8–15986.1)	14490.4(12612.8–16489.7)	0.14(0.10–0.19)*	1566.7(1136.9–2064.6)	1758.2(1265.5–2324.5)	0.40(0.34–0.47)*
Female	13611.8(11774.4–15674.8)	15043.2(12939.2–17388.8)	0.38(0.32–0.47)*	1796.2(1280.5–2404.7)	2087.2(1472.4–2808.5)	0.53(0.46–0.64)*
SDI						
Low	13079.5(11226.5–15100.5)	13696.9(11748.0–15863.9)	0.18(0.15–0.24)*	1658.0(1183.2–2220.9)	1821.6(1297.9–2448.7)	0.33(0.28–0.41)*
Low-Middle	13877.2(11947.1–15989.2)	14144.1(12181.4–16238.5)	0.12(0.07–0.19)*	1621.3(1164.3–2154.1)	1808.6(1287.1–2416.5)	0.41(0.34–0.53)*
Middle	13456.3(11695.9–15393.4)	14512.1(12593.8–16565.5)	0.28(0.23–0.34)*	1574.1(1134.0–2091.4)	1817.6(1294.6–2432.8)	0.52(0.45–0.60)*
High-Middle	13583.2(11820.7–15529.5)	15125.5(12996.7–17405.4)	0.35(0.34–0.38)*	1664.2(1200.1–2212.4)	1930.0(1367.7–2584.3)	0.51(0.48–0.55)*
High	15839.8(13874.4–17927.3)	19109.6(16650.2–21744.7)	0.70(0.63–0.80)*	2139.5(1547.2–2837.0)	2728.7(1958.7–3629.6)	0.92(0.82–1.05)*
Regions						
Andean Latin America	15679.3(13178.0–18553.7)	18726.5(15603.4–22512.3)	0.73(0.56–0.96)*	1834.7(1303.7–2493.1)	2341.2(1623.3–3222.1)	1.00(0.77–1.31)*
Australasia	22695.4(19732.4–25942.6)	24098.1(20921.1–27731.8)	0.22(0.20–0.25)*	3076.4(2205.5–4087.2)	3351.4(2413.1–4499.1)	0.31(0.28–0.34)*
Caribbean	17068.8(14533.3–20009.7)	18509.3(15538.5–21862.3)	0.29(0.25–0.34)*	1906.4(1366.9–2593.8)	2119.5(1497.9–2903.6)	0.40(0.34–0.48)*
Central Asia	11085.5(9537.1–12728.7)	12073.7(10,334–14021.5)	0.31(0.28–0.36)*	1406.2(1008.2–1877.2)	1602.2(1144.7–2152.6)	0.47(0.41–0.53)*
Central Europe	11672.4(10034.0–13502.9)	13371.8(11416.4–15555.7)	0.45(0.42–0.49)*	1477.5(1061.8–1962.3)	1777.6(1267.2–2392.8)	0.62(0.59–0.67)*
Central Latin America	13001.5(11225.7–14897.2)	15438.8(13230.8–17,875)	0.65(0.53–0.76)*	1649.5(1192.8–2195.5)	2069.2(1471.7–2788.4)	0.84(0.69–0.98)*
Central Sub-Saharan Africa	13336.1(11148.0–15785.4)	14302.1(11,931–17092.5)	0.27(0.21–0.37)*	1961(1,366–2676.4)	2112.5(1482.3–2937.8)	0.30(0.21–0.39)*
East Asia	12805.2(10984.7–14767.5)	12975.4(11038.8–15026.7)	−0.01(−0.07–0.03)	1415.9(1017.1–1883.1)	1374.7(977.4–1829.8)	−0.15(−0.19–−0.11)*
Eastern Europe	12162.9(10573.9–13862.9)	14,363(12447.5–16408.7)	0.54(0.54–0.55)*	1576.7(1127.2–2093.5)	1950.2(1382.1–2606.3)	0.70(0.68–0.71)*
Eastern Sub-Saharan Africa	12409.2(10638.7–14269.0)	13423.4(11469.3–15579.3)	0.27(0.25–0.30)*	1725.3(1230.2–2313.1)	1923.5(1362.3–2595.9)	0.37(0.34–0.41)*
High-income Asia Pacific	13149.8(11478.9–14872.2)	14382.6(12517.5–16338.6)	0.29(0.28–0.30)*	1746.3(1279.9–2285.9)	2011.6(1,460–2661.7)	0.46(0.45–0.47)*
High-income North America	17156.9(15108.4–19320.9)	21723.9(19114.2–24557.6)	0.83(0.72–0.93)*	2,315(1670.5–3051.8)	3176.8(2275.9–4199.3)	1.14(0.96–1.28)*
North Africa and Middle East	17871.5(15422.7–20554.0)	19460.6(16518.9–22810.8)	0.33(0.26–0.44)*	2329.5(1653.1–3131.2)	2682.6(1864.7–3654.8)	0.57(0.46–0.71)*
Oceania	12592.6(10555.9–14843.1)	13260.3(11012.5–15897.3)	0.15(0.09–0.17)*	1580.9(1113.5–2156.4)	1692.6(1165.1–2348.6)	0.19(0.12–0.22)*
South Asia	13839.3(11725.1–16111.8)	13648.3(11723.9–15757.1)	−0.02(−0.08–0.04)	1499.1(1,079–1988.1)	1637.2(1168.8–2181.7)	0.31(0.23–0.41)*
Southeast Asia	11789.9(10155.5–13544.0)	12546.4(10833.2–14446.6)	0.20(0.20–0.20)*	1410.6(1010.5–1884.4)	1644.2(1166.1–2213.5)	0.49(0.48–0.50)*
Southern Latin America	16536.4(14330.1–18966.0)	18954.3(15845.2–22448.7)	0.54(0.43–0.67)*	2302.6(1648.5–3086.9)	2730.7(1897.4–3728.7)	0.65(0.54–0.82)*
Southern Sub-Saharan Africa	11618.8(10054.5–13289.2)	13920.2(11936.8–16052.5)	0.64(0.60–0.71)*	1649.6(1185.4–2200.6)	2041.1(1449.9–2748.9)	0.75(0.67–0.82)*
Tropical Latin America	16786.3(14638.1–19263.0)	20051.4(17208.6–23077.9)	0.65(0.59–0.73)*	2087.4(1492.4–2791.1)	2,595(1827.7–3494.7)	0.77(0.69–0.90)*
Western Europe	18351.8(15912.4–21019.2)	20916.9(17839.6–24301.2)	0.55(0.46–0.67)*	2562.6(1832.8–3435.6)	3004.3(2128.7–4065.5)	0.65(0.55–0.81)*
Western Sub-Saharan Africa	11277.9(9644.2–13032.9)	11672.2(9985.6–13549.7)	0.17(0.12–0.22)*	1555.3(1109.5–2079.8)	1629.2(1156.5–2179.3)	0.22(0.17–0.29)*

DALYs, disability-adjusted life years; UI, uncertainty interval; AAPC, average annual percent change; CI, confidence interval; SDI, socio-demographic index. *p < 0.05.

Among the 10 categories of mental disorders, anxiety disorders had the highest ASP at 4968.0 (95% UI: 3639.4–6612.3) per 100,000 population in 2021, followed by depressive disorders at 3026.9 (95% UI: 2222.7–4111.1; Table 2). When stratified by sex, anxiety disorders ranked highest for both sexes, with ADHD following for males and depressive disorders for females. Higher prevalence rates of anxiety and depressive disorders were observed in regions with higher SDI, a trend consistent across other mental disorders. However, idiopathic developmental intellectual disability showed a decrease in ASP with increasing SDI.

**Table 2:** Global and regional age-standardized prevalence per 100,000 population (95% UI) of 10 mental disorders in adolescents and young adults, 2021.

Characteristics	Schizophrenia	Depressive disorders	Bipolar disorder	Anxiety disorders	Eating disorders	Autism spectrum disorders	Attention-deficit/hyperactivity disorder	Conduct disorder	Idiopathic developmental intellectual disability	Other mental disorders
Total	104.4(66.9–154.2)	3026.9(2222.7–4111.1)	410.8(297.4–567.1)	4968.0(3639.4–6612.3)	354.7(223.1–548.8)	831.3(701.1–975.6)	2179.5(1495.9–3085.1)	1802.9(1225.7–2,486)	1578.2(890.3–2246.9)	473.9(304.9–675.4)
Male	111(71.3–163)	2341.7(1708.6–3171.5)	395.3(287.7–543.4)	3781.6(2,751–5067.9)	250.8(148.5–417.3)	1101.7(930.3–1287.2)	3077.4(2118.6–4358.2)	2260.2(1560.9–3,068)	1,539(815.1–2241.7)	578.7(374.5–827)
Female	97.6(62.4–145)	3744.2(2753.1–5082.4)	427(307.9–591.8)	6212.9(4550.2–8,240)	463.1(298.1–696.3)	547(458.3–649.7)	1232.9(838.3–1755.4)	1318.4(850.4–1894.5)	1619.4(968.9–2250.6)	364.8(231.8–519.7)
SDI										
Low	89.1(55.0–138.9)	3207.0(2301.0–4395.7)	394.8(266.9–575.8)	4264.5(3014.3–5879.9)	223.6(138.1–355.1)	878(736.5–1031.1)	1165.5(784.6–1672.8)	1847.7(1264.9–2545.1)	1963.5(1107.4–2795.2)	473.3(302.2–673.4)
Low-Middle	101.5(63.8–152.1)	2986.8(2170.8–4075.4)	363.3(254–516.8)	4263.9(3093.2–5739.5)	288.2(179.4–455.4)	774.3(652.6–905.9)	1500.5(1012.5–2153.4)	1784.1(1209.0–2462.8)	2508.7(1526.1–3454.9)	448(285.8–640.6)
Middle	110.5(71.5–160.1)	2481.4(1826.6–3366.3)	393(285.6–533.4)	5236.4(3901.1–6838.4)	331.3(205.5–520.1)	754.3(632.8–888.7)	2708.6(1870.9–3838.7)	1761.8(1198.2–2434.8)	1220.7(651.5–1764.8)	432.8(274–618)
High-Middle	114.0(77.0–159.1)	2716.3(1957.5–3701.8)	368.4(256.4–525.8)	5585.1(4061.2–7449.9)	417.5(263.2–644.1)	850.3(714.3–1000.2)	3287.4(2269.5–4634.7)	1769.8(1213.6–2414.2)	543.2(180.2–897.4)	471.8(302.6–669.5)
High	110.2(73.8–159.3)	4821.9(3630.4–6367.3)	682.9(556.4–842.6)	6916.1(5,095–9183.3)	780.9(513.3–1179.6)	1113.6(936.1–1310.5)	3367.0(2282.1–4770.1)	1930.4(1327.4–2620.4)	361.7(66.4–681.6)	665.8(456.9–898.1)
Regions										
Andean Latin America	92.5(52.6–149.1)	2617.1(1799.3–3707.5)	818.6(524.5–1243.6)	8227.4(5398.3–12009.3)	533.5(326.7–824.2)	731.3(611.3–866.7)	4,336(2919.4–6206.4)	1855.9(1267.1–2540.4)	429.3(109–728.5)	528.9(339–732.2)
Australasia	193.1(157.6–234.1)	5,353(3798.6–7564.2)	1292.7(979.0–1658.2)	7513.2(5036.1–10831.2)	1644.3(1,136–2,368)	1245.6(1039.9–1,488)	6449.5(4644.6–8575.4)	1987.7(1381.0–2678.5)	296.6(50.2–597.1)	824.6(630.0–1047.8)
Caribbean	89.1(51.5–143.4)	3016.4(2075.6–4,352)	821.0(522.9–1248.7)	5934.7(3887.3–8648.4)	398.3(250.6–618)	723.3(607.7–859.5)	6065.0(4187.4–8581.7)	1836.5(1240.5–2542.3)	535.1(150.9–905.4)	526.7(337.5–729)
Central Asia	77.9(43.8–130.3)	2790.5(1945.1–3870.5)	376.8(234.4–585.4)	3047.9(2046.9–4471.4)	291.8(185.3–454.2)	945.7(794.2–1112.8)	2027.9(1368.5–2925.7)	1894.4(1291.9–2614.7)	724.5(265.9–1159.1)	528.6(338.7–731.7)
Central Europe	77.8(45.8–124.4)	2311.0(1664.5–3153.8)	408.6(279.7–591.9)	5045.7(3564.9–7016.2)	370.7(235.6–574.9)	1015.6(852.4–1198.2)	2063.0(1396.7–2950.8)	1947.5(1,340–2662.4)	387.1(57.8–715.4)	489.2(313.7–694.6)
Central Latin America	92.8(57.4–139.7)	2755.6(1996.3–3766.7)	818.8(595–1108.5)	5909.7(4231.2–8082.9)	451.4(278.6–703.6)	810.8(682.2–957.4)	2878.3(1976.4–4087.2)	1912.4(1321.1–2,627)	352.5(59.9–642.5)	463.6(296.1–663.1)
Central Sub-Saharan Africa	79.0(44.8–132.9)	4624.1(3119.0–6681.9)	410.1(255.1–632.3)	4566.4(2999.9–6800.7)	211.7(131.3–333.5)	957.9(801.6–1133.8)	1020.3(680.5–1488.8)	1855.2(1252.6–2560.2)	909.3(365.9–1414.5)	526.0(337.1–728.1)
East Asia	133.3(91.0–181.1)	1278.0(963–1666.2)	145.7(106.7–193.8)	4410.6(3311.0–5781.4)	256.3(155.7–414.4)	696.4(581.6–828.2)	4407.0(3072.9–6212.5)	1527.9(1032.0–2112.0)	376.8(112.0–647.5)	414.1(259.3–592.1)
Eastern Europe	74.7(47.7–109.5)	2940.2(2117.5–4015.3)	361.3(261.4–492.4)	5418.4(4069.1–7113.4)	364.5(231.5–558.8)	991.3(832.1–1178.3)	2122.0(1423.9–3057.2)	2042.0(1419.5–2768.7)	490.3(115.8–864)	416.7(261.6–596.1)
Eastern Sub-Saharan Africa	80.3(48.2–126.6)	3598.6(2584.6–4926.9)	463.4(316.3–670.5)	4666.6(3319.5–6420.2)	214.4(132.5–342.5)	964.6(813.3–1130.1)	1030.9(688.4–1487.9)	1922.6(1329.1–2621.4)	765.2(276.5–1255.4)	476.7(304.5–677)
High-income Asia Pacific	91.4(56.8–137.7)	2710.3(2028.1–3549.6)	420.6(304.8–567.7)	4391.9(3195.8–5882.7)	783.7(520.2–1156.7)	1630.7(1374.3–1915.3)	2708.0(1833.8–3888.1)	1919.2(1309.7–2605.7)	80.6(3.0–268.9)	553.0(366.1–760.7)
High-income North America	129.8(89.3–178.1)	6870.3(5,303–8820.3)	764(687.8–841.3)	6685.1(4989.4–8613.6)	831.2(535.5–1273.8)	1159.6(977.2–1366.9)	4257.6(2822.9–6097.3)	1836.5(1,243–2533.3)	490.5(84.4–895.3)	817.7(569.7–1,100)
North Africa and Middle East	96.9(57.6–156.3)	4519.7(3101.6–6396.3)	700.5(466.3–1044.3)	8169.8(5691.4–11376.9)	402.2(247.2–637.2)	808.3(679.8–952.5)	2346.4(1616.9–3331.8)	1708.4(1151.9–2349.1)	1832.9(1024.3–2595.1)	516(330.0–718.9)
Oceania	113.4(64.4–185.8)	2449.9(1683.5–3497.7)	194(119.2–303.6)	5063.2(3256.8–7648.9)	191.8(118.5–300.4)	719.9(605.8–862.6)	2215.2(1,510–3193.8)	1709.1(1171.5–2362.8)	797.5(313.2–1269.1)	530.6(340–734.5)
South Asia	105.7(68.2–154.2)	2714.2(2012–3664.8)	238.4(170.5–330.5)	3,189(2353.2–4202.7)	286.2(176.2–452.8)	735.7(619.6–859.4)	1137.9(757.5–1632.7)	1760.7(1192.8–2455.5)	3868.2(2494.9–5226.2)	418.3(263–598.5)
Southeast Asia	126.6(80.7–187.8)	2285.9(1634.3–3138.9)	230.4(156.7–329.8)	4642.5(3384.5–6214.7)	243.9(151.1–384.1)	723.3(607–855.2)	1842.7(1,247–2646.9)	1832.4(1251.2–2537.2)	817.6(367.6–1254.7)	454.5(290.1–650.8)
Southern Latin America	98.3(55.7–164.9)	4224.3(3006.3–5702.9)	700(454.9–1052.1)	8018.8(5131.5–11590.2)	662.2(421.6–1013.6)	1126.1(944.3–1325.2)	2,600(1756.4–3752.9)	1937(1317.1–2666.3)	463.6(77–847.5)	656.5(434.7–900.9)
Southern Sub-Saharan Africa	81.7(51.2–123.4)	3581.5(2620.1–4773.9)	398.1(279.6–557)	5519.3(4092.7–7277.4)	325.1(200.2–517.2)	975.1(821.7–1154.3)	1043.2(693.1–1502.9)	1965.3(1338.2–2709.4)	378.3(79.4–693.3)	444.2(283.2–634.8)
Tropical Latin America	93.1(60.3–134.9)	3,151(2334.6–4258.7)	1164.6(883.3–1,483)	9131.2(6912.4–11794.8)	468.3(292.5–712.6)	660.2(553–784.2)	4298.3(2926.5–6159.3)	1944.1(1335.7–2667.6)	402.3(97.8–689.6)	409.0(255.8–584.5)
Western Europe	87.5(56.0–134.4)	4630.4(3264.2–6,438)	801.8(582.3–1094.2)	9358.4(6736.9–12677.9)	879.9(580.7–1314.5)	949.1(798.2–1113.5)	2837.6(1890.7–4020.2)	2171.6(1511.5–2916.8)	434.8(121.3–768.4)	611.6(404.7–838.1)
Western Sub-Saharan Africa	87.0(53.7–134.6)	2631.1(1875.4–3588.7)	371.7(254.9–535.1)	4,010(2885.3–5463.8)	258.1(160.1–410.2)	951.8(801.4–1119.3)	1077.1(710.5–1571.2)	1884(1285.4–2586.9)	506.3(125.3–909.3)	463.3(295.9–661.1)

UI, uncertainty interval; SDI, socio-demographic index.

In terms of age-standardized DALYs, anxiety disorders again ranked highest at 609.4 (95% UI: 377.5–909.1) per 100,000 population, followed by depressive disorders at 564.5 (95% UI: 351.2–853) per 100,000 population, with this pattern consistent across sexes. DALYs for these disorders also increased with higher SDI, while idiopathic developmental intellectual disability exhibited a decline (Supplementary Table S2).

Regarding overall temporal trends from 1990 to 2021, anxiety and depressive disorders exhibited the highest AAPC in ASP, with depressive disorders at 0.76 (95% confidence interval [CI]: 0.64–0.93) and anxiety disorders at 0.68 (95% CI: 0.58–0.80). Other disorders showing an increase include eating disorders, bipolar disorder, autism spectrum disorders, conduct disorder, and other mental disorders. In contrast, ADHD, idiopathic developmental intellectual disability, and schizophrenia experienced declines (Supplementary Table S3). The trends in AAPC for these disorders were consistent across sexes. Further detailed results regarding ASP and age-standardized DALYs for 10 mental disorders in 2021, as well as the AAPC from 1990 to 2021 across 204 countries and territories, can be found in Supplementary Figures S1–S10.

**Figure 2:**
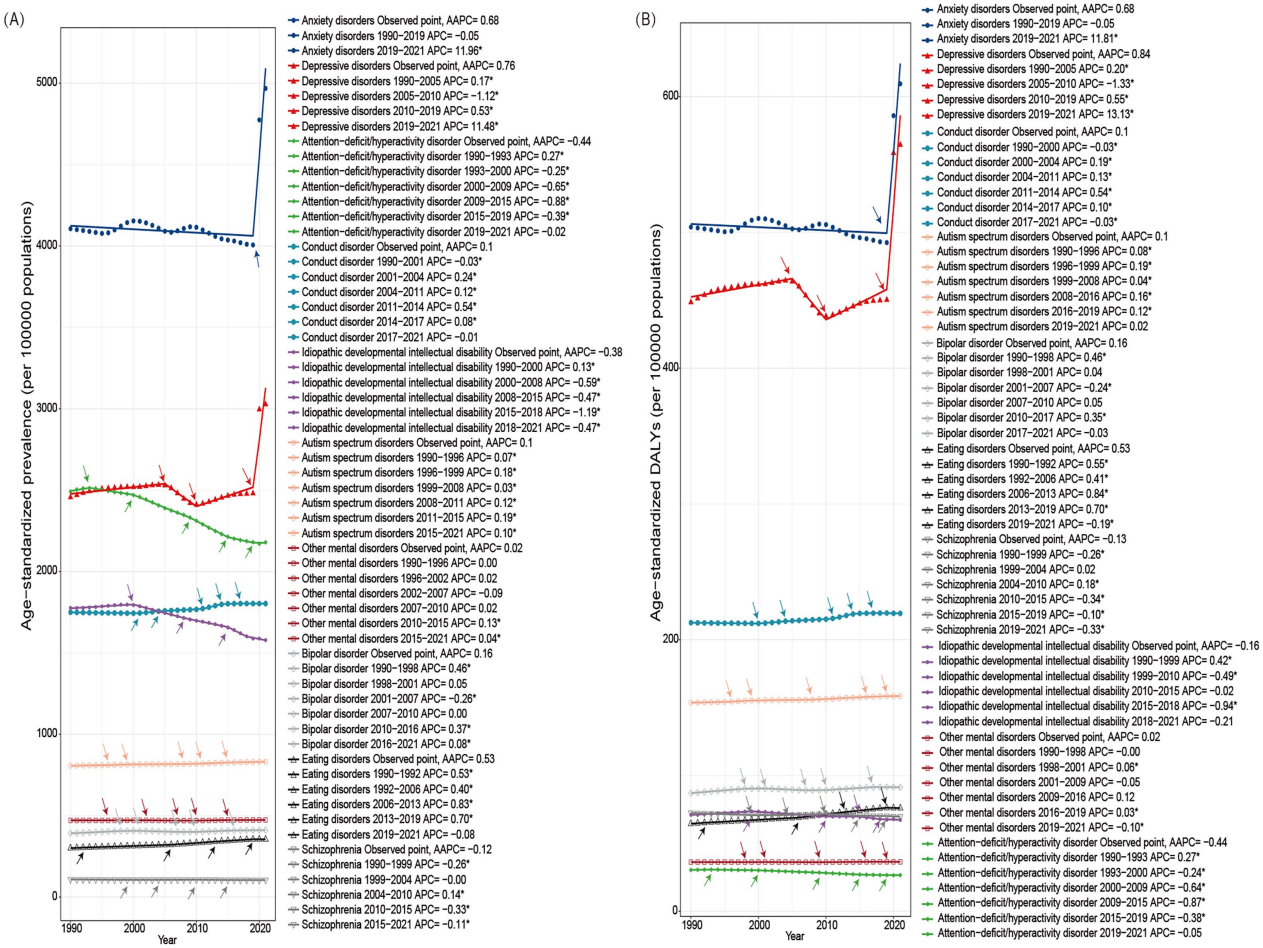
Temporal trends in age-standardized prevalence (ASP) and age-standardized disability-adjusted life years (DALYs) for 10 mental disorders among adolescents and young adults aged 10–24 years at the global level from 1990 to 2021. Panel **(A)** shows trends in ASP (per 100,000 population), and panel **(B)** shows trends in age-standardized DALYs (per 100,000 population). Observed data points represent GBD 2021 estimates for each year. Trend lines were fitted using joinpoint regression models, and arrows indicate statistically significant joinpoints where temporal trends changed. The annual percentage change (APC) is reported for each identified time segment, and the average annual percentage change (AAPC) summarizes the overall trend across the study period.

Notably, Joinpoint analysis revealed significant turning points in the ASP of depressive and anxiety disorders, with sharp increases observed in 2020 and 2021 (Figure 2). Correspondingly, age-standardized DALYs for these two disorders also rose markedly during this period, while other disorders exhibited varying degrees of increase. In contrast, ADHD and idiopathic developmental intellectual disability showed declines in DALYs (Figure 2; Supplementary Table S4). Stratified analyses by SDI and sex also indicated consistent sharp increases in ASP and age-standardized DALYs during 2020 and 2021, particularly for anxiety and depressive disorders (Supplementary Figures S11, S12). Further detailed results regarding the temporal trends and joinpoints for 10 mental disorders stratified by SDI can be found in Supplementary Figures S13–S22.

**Figure 3:**
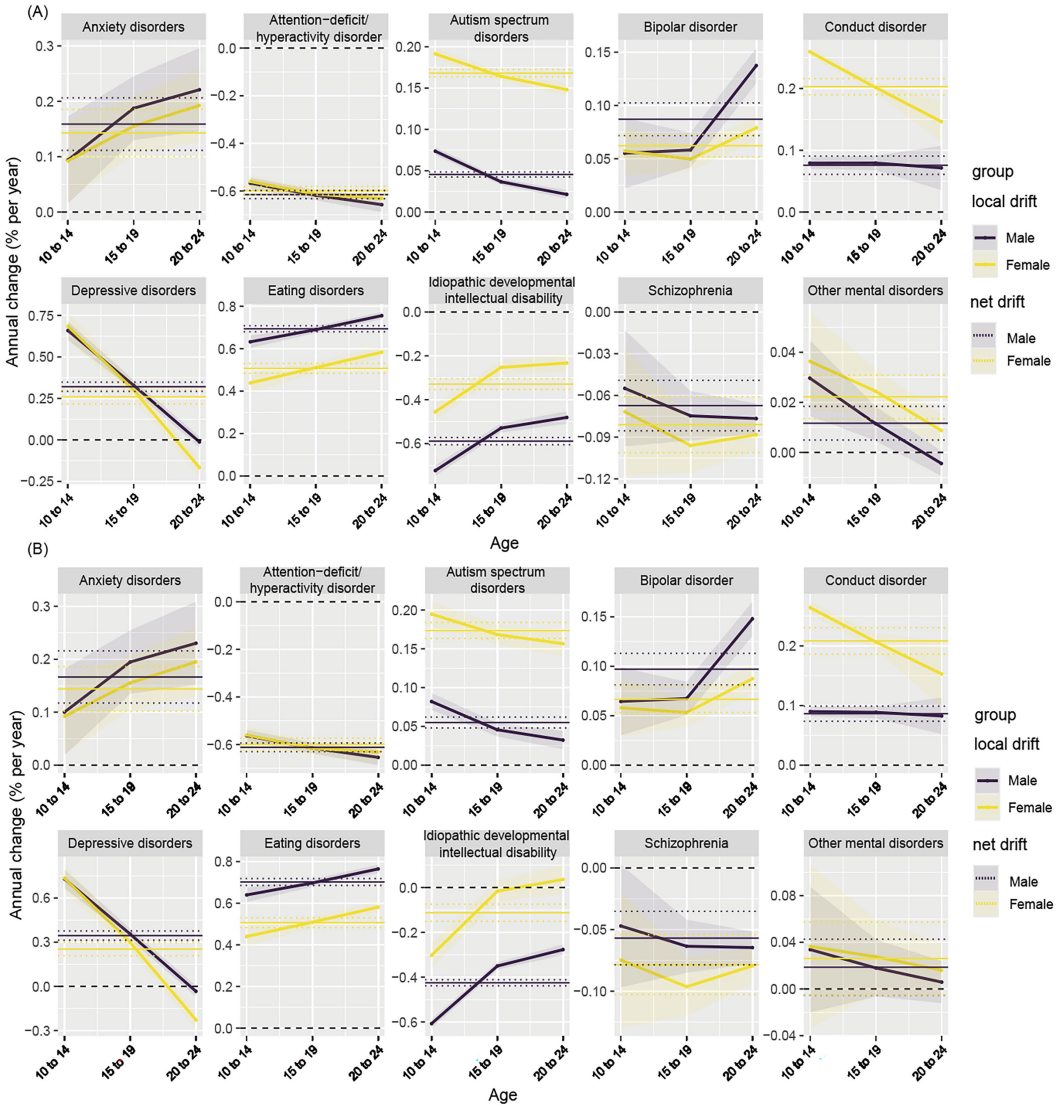
Age-specific annual percent change (APC) in prevalence and disability-adjusted life years (DALYs) for 10 mental disorders by sex from 1990 to 2021. Panel **(A)** presents local drift estimates and net drift for prevalence, and Panel **(B)** presents local drift estimates and net drift for DALYs across age groups (10–14, 15–19, and 20–24 years). Solid lines represent local drift estimates for males and females, reflecting age-specific annual percentage changes within each age group. Dashed lines indicate net drift, which represents the overall APC across all age groups combined. Positive APC values indicate increasing trends over time, whereas negative values indicate decreasing trends.

Consistent with the above findings, the age-period-cohort model showed that the net drift, representing the overall APC after accounting for age and cohort effects, demonstrated similar temporal patterns for both prevalence and DALYs of mental disorders. Most disorders, including anxiety disorders, depressive disorders, eating disorders, bipolar disorder, autism spectrum disorders, and conduct disorder, exhibited significant upward trends with positive net drift values, whereas ADHD, idiopathic developmental intellectual disability, and schizophrenia showed negative net drift values, indicating declining trends over time. Local drift analyses further revealed age-specific differences in temporal trends. As shown in Figure 3, the APC for anxiety disorders increased with age, whereas the APC for depressive disorders decreased with age in both sexes. Overall, positive local drift values indicate increasing burden over time, with higher APCs observed among older age groups for several mental disorders. Although joinpoint regression models may yield different temporal segments across age strata, the overall direction of trends was consistent across age groups, with age-related differences primarily reflected in the magnitude of APCs rather than in the timing of joinpoints. Detailed age, period, and cohort effects are presented in Supplementary Figures S23–S25.

Further analysis revealed that the increasing trends in DALYs for anxiety disorders, autism spectrum disorders, bipolar disorder, conduct disorder, depressive disorders, and eating disorders were attributed to both epidemiological changes and population growth, and the increasing trend for ADHD and idiopathic developmental intellectual disability, as well as schizophrenia, was primarily driven by population growth (Supplementary Figure S26).

## 4 Discussion

Adolescence and young adulthood represent a distinctive and formative phase characterized by significant physical, emotional, and social changes (5). During this time, exposure to adverse environmental factors can heighten vulnerability to mental health issues, potentially leading to early onset of disorders that have lasting negative effects on lifelong health and significantly contribute to the global disease burden (1, 16). In this study, we reveal a significant increase in the ASP and age-standardized DALYs for mental disorders among adolescents and young adults from 1990 to 2021 globally, with anxiety and depressive disorders remained exhibiting the highest rates. The increase in both ASP and age-standardized DALYs was particularly pronounced among females and was consistent across all SDI regions, with higher SDI regions experiencing more rapid growth. Notably, the analysis revealed that this rise was relatively steady from 1990 to 2019, but there were sharp increases in ASP and age-standardized DALYs for anxiety and depressive disorders in 2020 and 2021, coinciding with the global onset of the COVID-19 pandemic (17).

The COVID-19 pandemic has been widely recognized as a major global stressor with potential impacts on mental health, particularly among adolescents and young adults (17). In the present study, we observed a positive net drift in anxiety and depressive disorders, indicating an overall increasing temporal trend that extends beyond age- or cohort-specific effects. Consistent with this, decomposition analysis suggested that rising DALYs were driven by both population growth and unfavorable epidemiological changes. The increase observed during 2020–2021 coincided with the COVID-19 period and may partly reflect pandemic-related disruptions, however, these findings suggest that the observed trends are likely shaped by longer-term social, economic, and healthcare-related changes rather than the effect of the pandemic alone. Within this broader temporal context, the pronounced increases observed during 2020 and 2021 may have been amplified by pandemic-related stressors. Disruptions to daily life, including social isolation and the abrupt transition to remote learning, may have adversely affected mental well-being among adolescents and young adults by reducing social interaction and support (18). Heightened concerns about health risks to oneself and family members, along with widespread uncertainty related to infection risk, economic instability, and future prospects, may have further contributed to increased anxiety and depressive symptoms during this period (19, 20). In addition, disruptions in access to mental health services and support systems during the pandemic may have exacerbated existing mental health challenges by limiting timely care and intervention (21, 22). Collectively, these factors underscore the potential causes of the rising anxiety and depressive disorders during the pandemic. Furthermore, it is imperative to emphasize the need for future research to extend the follow-up period and facilitate a comprehensive examination of mental health changes beyond the COVID-19 pandemic.

We observed a higher prevalence of anxiety and depressive disorders among females compared to males globally, a trend consistently reported across various studies (23, 24). This disparity may be attributed to sociocultural and biological factors. During the pandemic, women were more likely to experience financial disadvantage and increased rates of domestic violence, particularly during lockdowns and stay-at-home orders (23). Additionally, gender socialization, which encourages females to express emotions and seek help, may contribute to the higher prevalence observed in this demographic (25).

Furthermore, our observations indicate a correlation between higher SDI regions and elevated rates of anxiety and depressive disorders, which seemingly contradicts the prevailing notion that poorer financial circumstances are a primary risk factor for such conditions (26, 27). In high SDI regions, competitive environments and heightened societal expectations may contribute to mental health challenges (28, 29). Additionally, higher SDI areas often provide better access to education and healthcare, resulting in increased awareness and diagnosis of mental health issues (30). In this context, it is noteworthy that East Asia and South Asia were the only regions without a statistically significant increase in age-standardized prevalence, which may reflect underdiagnosis, cultural stigma, and heterogeneous data quality rather than a truly stable burden. Beyond higher baseline prevalence, faster growth rates observed in high-SDI regions may reflect intensifying social stressors alongside increasing mental health awareness, improved diagnostic capacity, and expanded service coverage over time (31), whereas growth in lower-SDI regions may be attenuated by persistent underdiagnosis and data limitations.

Decomposition analysis highlighted heterogeneous drivers of DALYs change across mental disorders. Increases in DALYs for autism spectrum disorders, bipolar disorder, conduct disorder and eating disorders were driven by both population growth and unfavorable epidemiological changes, whereas DALYs increase for ADHD, idiopathic developmental intellectual disability, and schizophrenia were largely attributable to population growth, suggesting relatively stable underlying epidemiological patterns for these conditions (32). Anxiety disorders illustrate how the drivers of DALY changes vary across SDI settings. Overall, increases in anxiety-related DALYs were attributable to both epidemiological change and population growth. However, in high-SDI regions, the increase was predominantly driven by unfavorable epidemiological changes, suggesting a rising underlying risk or incidence of anxiety disorders. In contrast, in low- and middle-SDI regions, population growth contributed more substantially to DALY increases, indicating that demographic expansion rather than marked changes in epidemiological risk was the primary driver. These differences underscore the need for disorder-specific and SDI-specific strategies.

This study provides a comprehensive assessment of long-term trends in the global burden of mental disorders among adolescents and young adults from 1990 to 2021, but several limitations should be acknowledged. First, data quality remains a concern, as estimates are derived from heterogeneous and often sparse sources, particularly in low- and middle-SDI regions, which may lead to underestimation of the true burden. Second, most data are based on DSM-IV and ICD-10 classifications, while newer diagnostic systems such as DSM-5 and ICD-11 have not yet been fully incorporated. In addition, the scope of analysis was limited to mental disorders with sufficient global epidemiological data available in GBD 2021, and all estimates rely on statistical modeling. Although more recent GBD updates (GBD 2023) have become available, the use of GBD 2021 allows for internally consistent analyses of long-term trends up to 2021; future studies may benefit from incorporating updated releases as they emerge. Finally, current GBD methodologies do not fully capture premature mortality attributable to mental disorders or broader welfare losses associated with disability, potentially underestimating their overall societal impact.

These findings have several implications for mental health interventions among adolescents and young adults. For disorders in which DALY increases were driven by unfavorable epidemiological changes, particularly anxiety and depressive disorders, preventive strategies targeting modifiable social and environmental stressors should be prioritized. In high-SDI settings, where both prevalence and growth rates were elevated, expanded early screening, timely diagnosis, and youth-oriented mental health services are warranted, while in lower-SDI regions, efforts should focus on improving access to basic mental health care, strengthening surveillance systems, and reducing underdiagnosis.

## 5 Conclusion

This study highlights the significant trends in the global burden of mental disorders among adolescents and young adults from 1990 to 2021, revealing alarming increases in anxiety and depressive disorders, particularly during the COVID-19 pandemic. The observed disparities in prevalence rates between sexes and across different SDI regions further emphasize the complexity of mental health issues, suggesting that higher socioeconomic status does not necessarily equate to better mental health outcomes. Future research must extend the follow-up period to explore long-term mental health changes beyond the COVID-19 pandemic and consider the implications of these trends for public health policy and mental health services. Addressing the rising burden of mental disorders requires a comprehensive understanding of the underlying factors and a commitment to improving data quality and access to mental health resources, particularly for vulnerable populations.

## Data Availability

Publicly available datasets were analyzed in this study. This data can be found at: https://vizhub.healthdata.org/.

## Footnotes

1
https://www.healthdata.org/
