# Supplementary material for: Trends in the prevalence and burden of mental disorders among adolescents and young adults, 1990–2021
Source: Front Public Health. 2026 Feb 19;14:1767326. doi: 10.3389/fpubh.2026.1767326 (PMC12960524; doi:10.3389/fpubh.2026.1767326)
Supplement: Supplementary file 1 [file Data_Sheet_1.docx]

Supplementary Material

# **Supplementary methods**

**Definition of mental disorders in GBD 2021:**

| Anxiety disorders | Anxiety disorders incorporate disability caused by experiences of intense fear and distress in combination with other physiological symptoms. They are modelled as a single cause including at least three subtypes of anxiety disorders defined according to DSM-IV-TR and ICD-10 diagnostic criteria. |
| --- | --- |
| Attention-deficit/hyperactivity disorder (ADHD) | ADHD is an externalising disorder, incorporating disability from persistent inattention and/or hyperactivity-impulsivity. DSMIV-TR (314.0, 314.01) and ICD-10 (F90) diagnostic criteria were used. |
| Autism spectrum disorders (ASD) | ASD is a neurodevelopmental disorder with onset occurring in early childhood. It incorporates disability from pervasive impairment in several areas of development, including social interaction and communication skills, along with restricted and repetitive patterns of behaviours and/or interests. It was defined according to DSM-5 (299) and ICD-10 (F84) diagnostic criteria. |
| Bipolar disorder (BP) | BP is a chronic mood disorder, incorporating disability from manic or hypomanic episodes, accompanied by major depressive episodes. Burden was estimated for the entire spectrum of bipolar disorder rather than by subtype. DSM-IV-TR (296.0–296.7, 296.89, 301.13) and ICD-10 (F30.0-F30.9, F31.0–F31.6, F31.8–F31.9, F34.0) diagnostic criteria were used. |
| Conduct disorder (CD) | CD occurs in those aged below 18 years and incorporates disability from antisocial behaviour that violates basic rights of others or major age-appropriate societal norms. DSM-IV-TR (312.81–312.89) and ICD-10 (F91) diagnostic criteria were used. |
| Depressive disorders | This aggregate cause incorporates disability from major depressive disorder (MDD) and dysthymia. MDD involves the experience of depressed mood almost all day, every day, for two weeks. Dysthymia symptoms are less severe but chronic. DSM/ICD criteria were used. |
| Eating disorders | This aggregate cause incorporates deaths and disability from anorexia nervosa (AN) and disability from bulimia nervosa (BN). These are characterised by abnormal eating behaviours and concerns over food, eating, and body image. DSM-IV-TR (307.1, 307.51) and ICD-10 (F50.0, F50.2) criteria were used. |
| Idiopathic developmental intellectual disability (ID) | This cause captures the health loss resulting from intellectual disability that arises from environmental sources and unknown aetiologies. |
| Schizophrenia | Schizophrenia is a chronic psychotic disorder which involves the experience of positive symptoms (eg, delusions, hallucinations, thought disorder) and negative symptoms (eg, flat affect, loss of interest, emotional withdrawal). DSM-IV-TR (295.10-295.30, 295.60, 295.90) and ICD-10 (F20) diagnostic criteria were used. |
| Other mental disorders | This is a residual cause incorporating disability from personality disorders. Personality disorders are characterised by pervasive, inflexible, and maladaptive patterns of behaviour, which are markedly different from what is considered to be culturally acceptable. DSMIV-TR and ICD-10 diagnostic criteria were used. |

Supplementary Material should be uploaded separately on submission. Please include any supplementary data, figures and/or tables.

Supplementary material is not typeset so please ensure that all information is clearly presented, the appropriate caption is included in the file and not in the manuscript, and that the style conforms to the rest of the article.

# Supplementary Figures and Tables

## Supplementary Figures


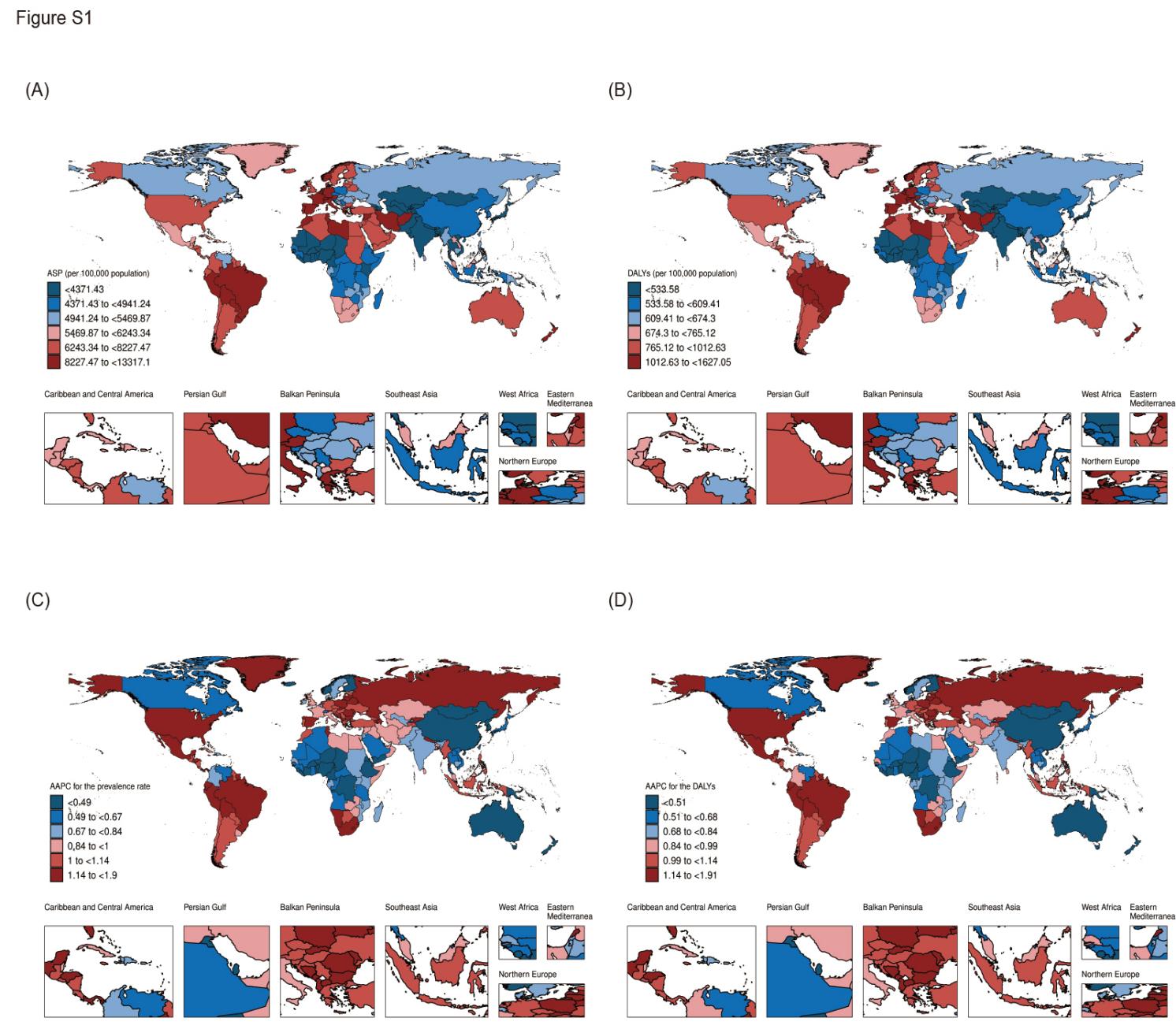


**Supplementary Figure 1.** Global map of (A) age-standardized prevalence (ASP) and (B) age-standardized disability-adjusted life years (DALYs) for anxiety disorders in individuals aged 10-24 in 2021, and average annual percent change in (C) ASP and (D) DALYs from 1990 to 2021 across 204 countries and territories.


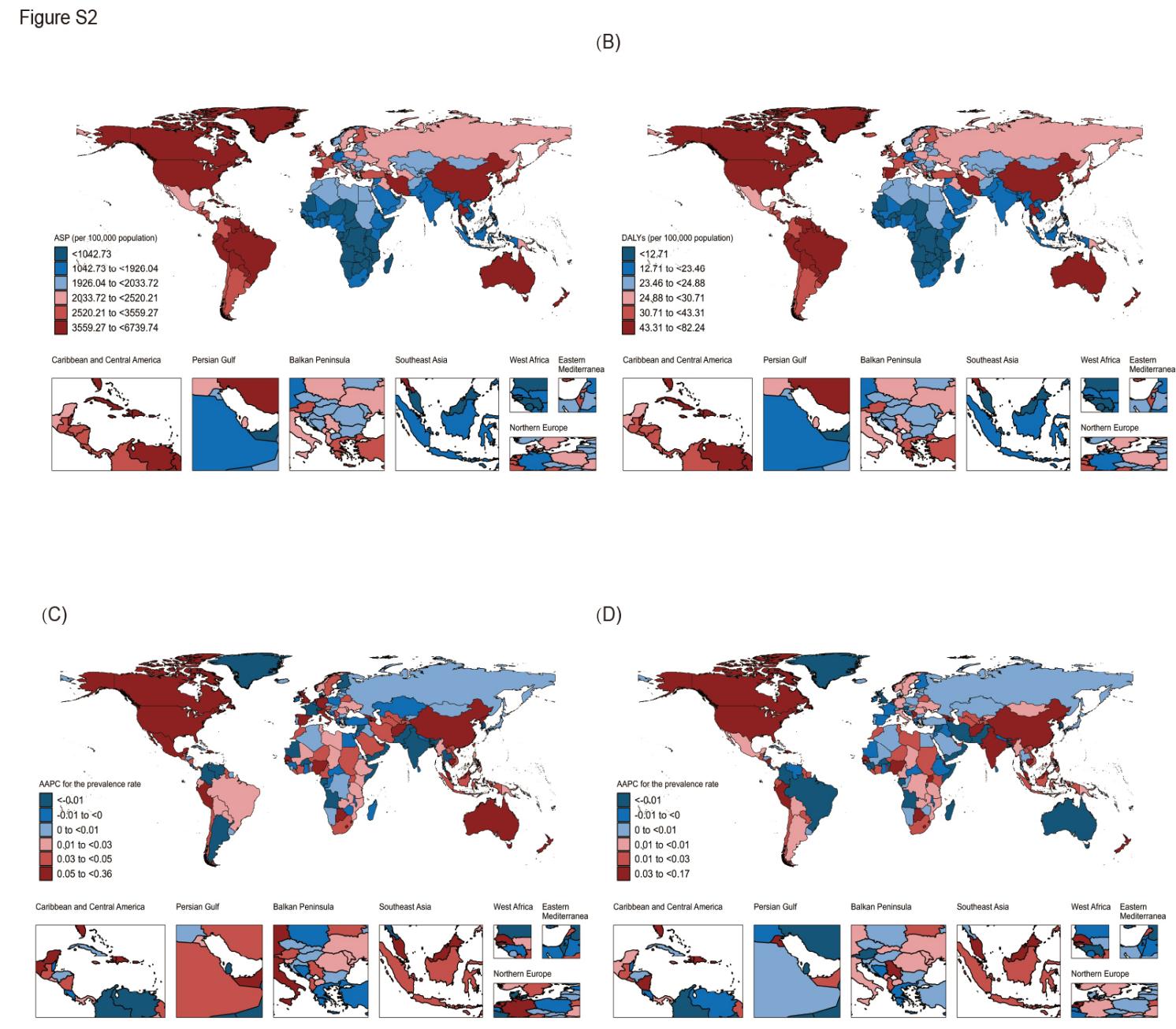


**Supplementary Figure 2.** Global map of (A) age-standardized prevalence (ASP) and (B) age-standardized disability-adjusted life years (DALYs) for attention−deficit/hyperactivity disorder in individuals aged 10-24 in 2021, and average annual percent change in (C) ASP and (D) DALYs from 1990 to 2021 across 204 countries and territories.


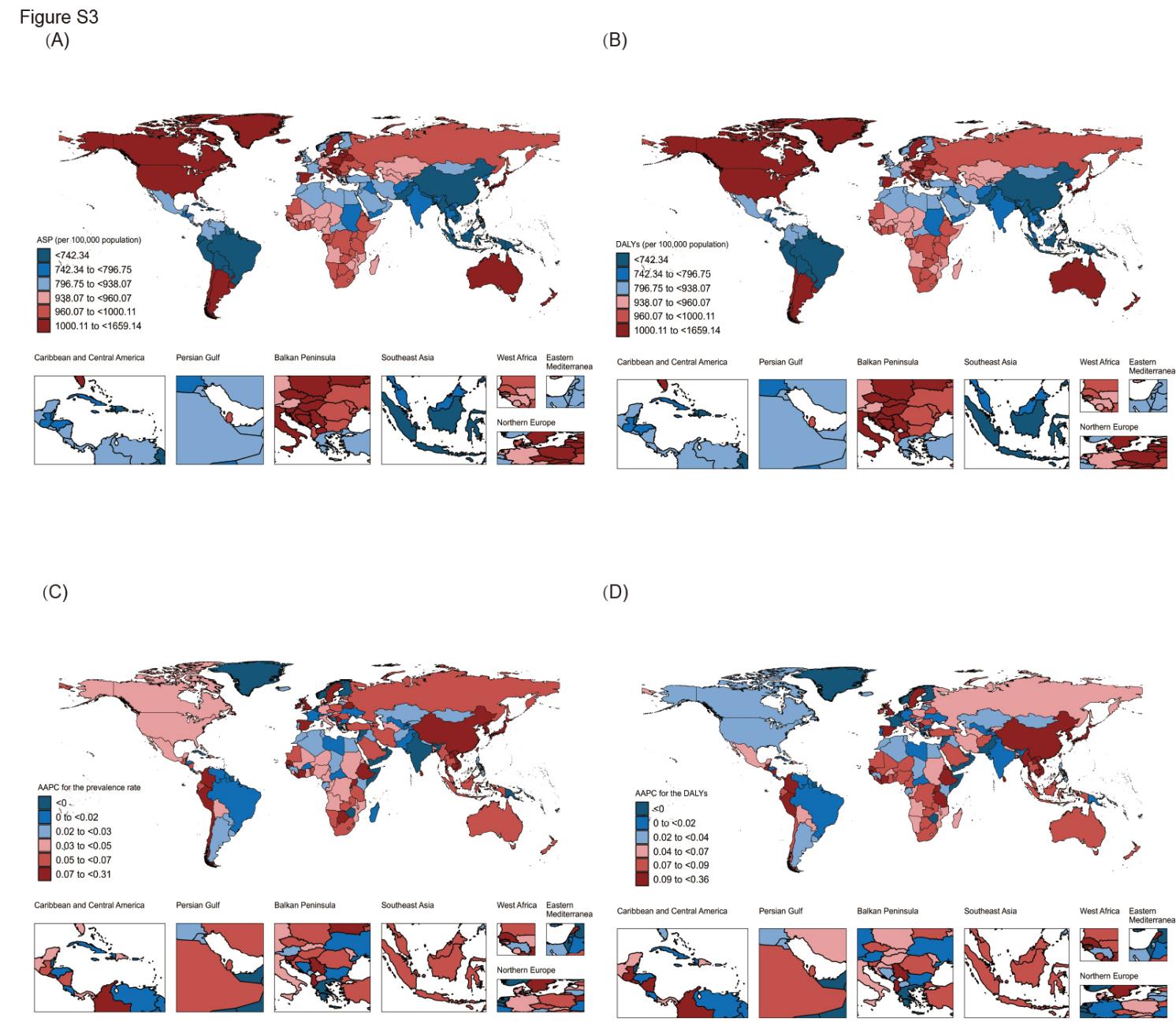


**Supplementary Figure 3.** Global map of (A) age-standardized prevalence (ASP) and (B) age-standardized disability-adjusted life years (DALYs) for autism spectrum disorders in individuals aged 10-24 in 2021, and average annual percent change in (C) ASP and (D) DALYs from 1990 to 2021 across 204 countries and territories.


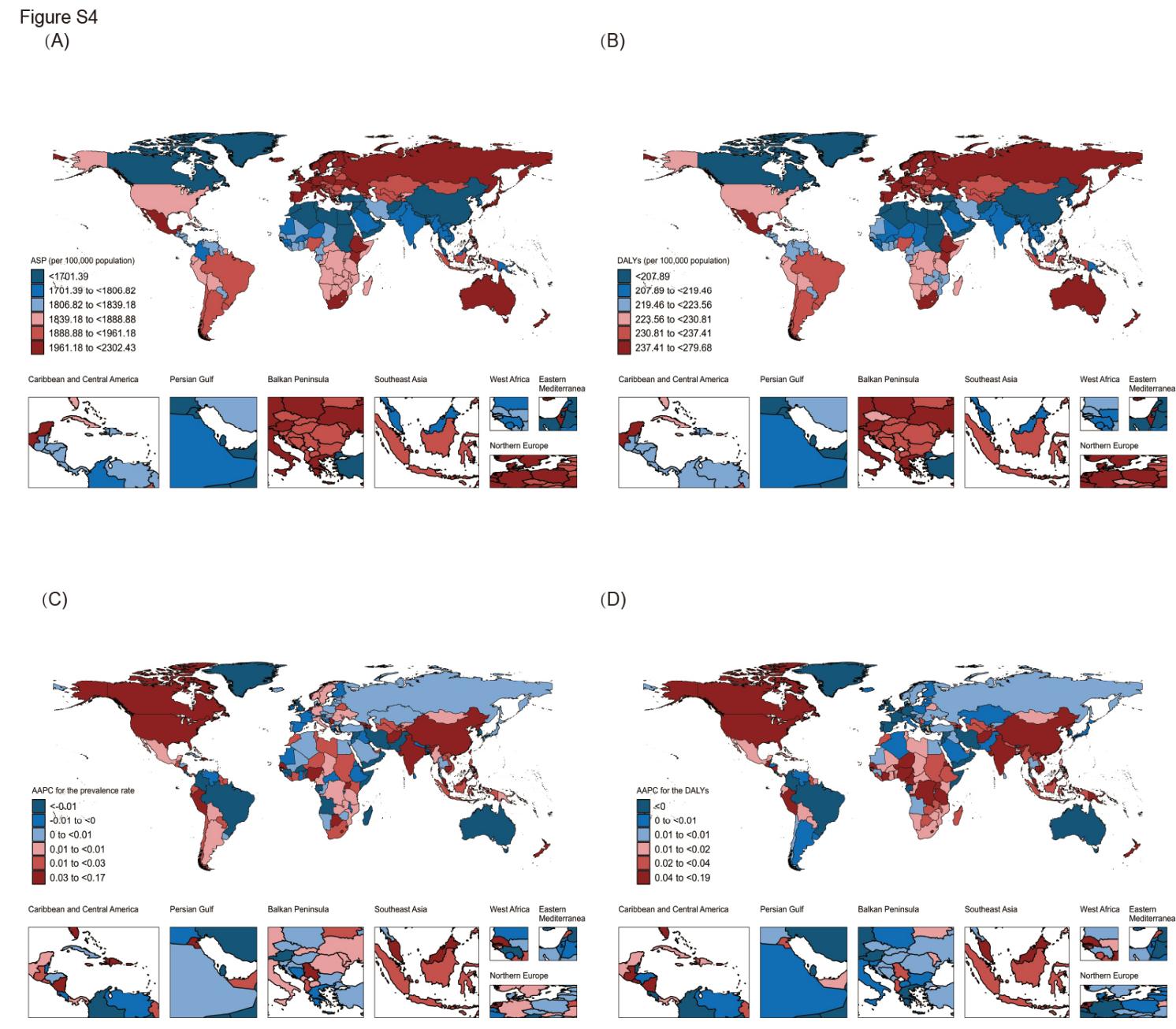


**Supplementary Figure 4.** Global map of (A) age-standardized prevalence (ASP) and (B) age-standardized disability-adjusted life years (DALYs) for bipolar disorder in individuals aged 10-24 in 2021, and average annual percent change in (C) ASP and (D) DALYs from 1990 to 2021 across 204 countries and territories.


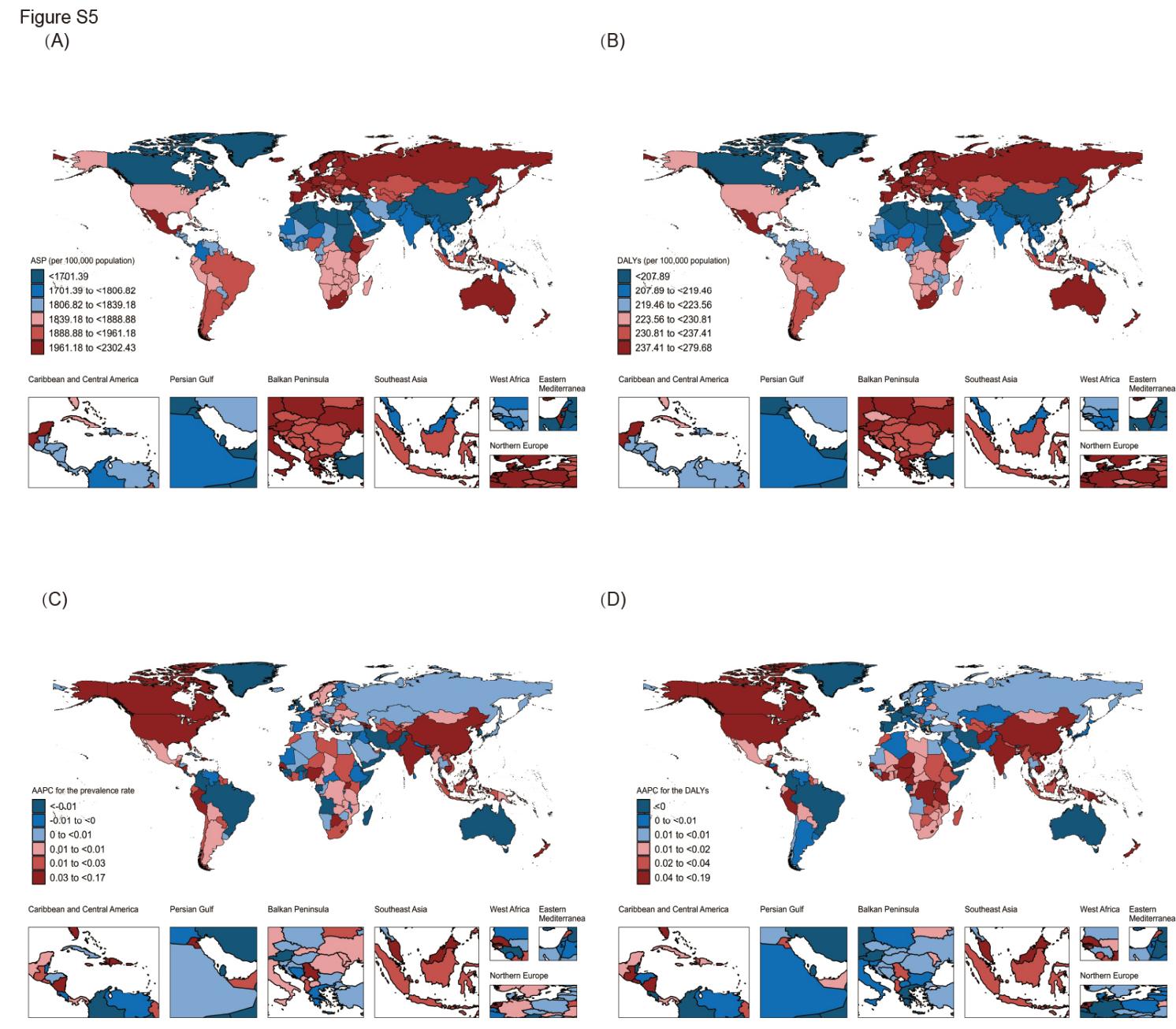


**Supplementary Figure 5.** Global map of (A) age-standardized prevalence (ASP) and (B) age-standardized disability-adjusted life years (DALYs) for conduct disorder in individuals aged 10-24 in 2021, and average annual percent change in (C) ASP and (D) DALYs from 1990 to 2021 across 204 countries and territories.


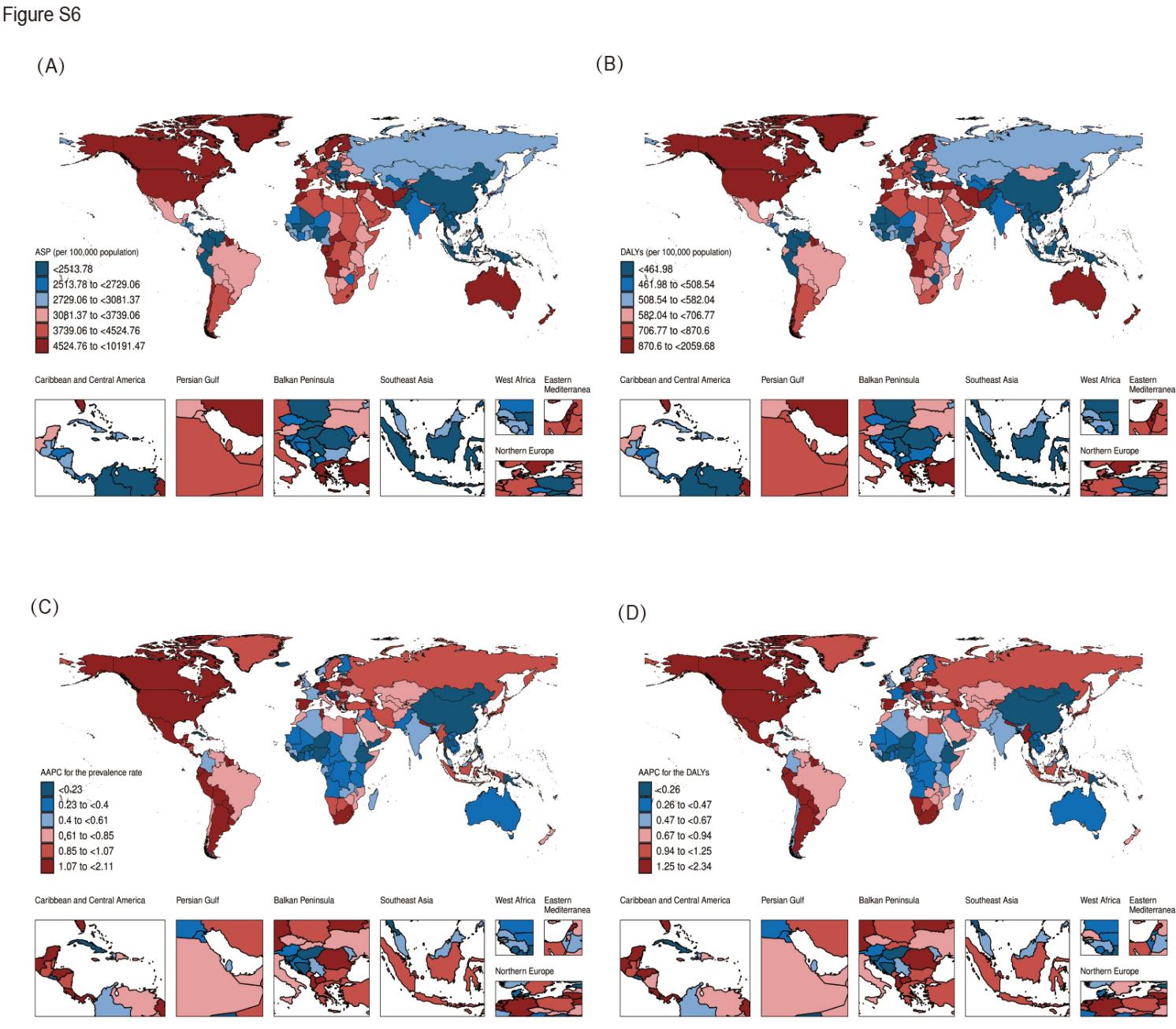


**Supplementary Figure 6.** Global map of (A) age-standardized prevalence (ASP) and (B) age-standardized disability-adjusted life years (DALYs) for depressive disorders in individuals aged 10-24 in 2021, and average annual percent change in (C) ASP and (D) DALYs from 1990 to 2021 across 204 countries and territories.


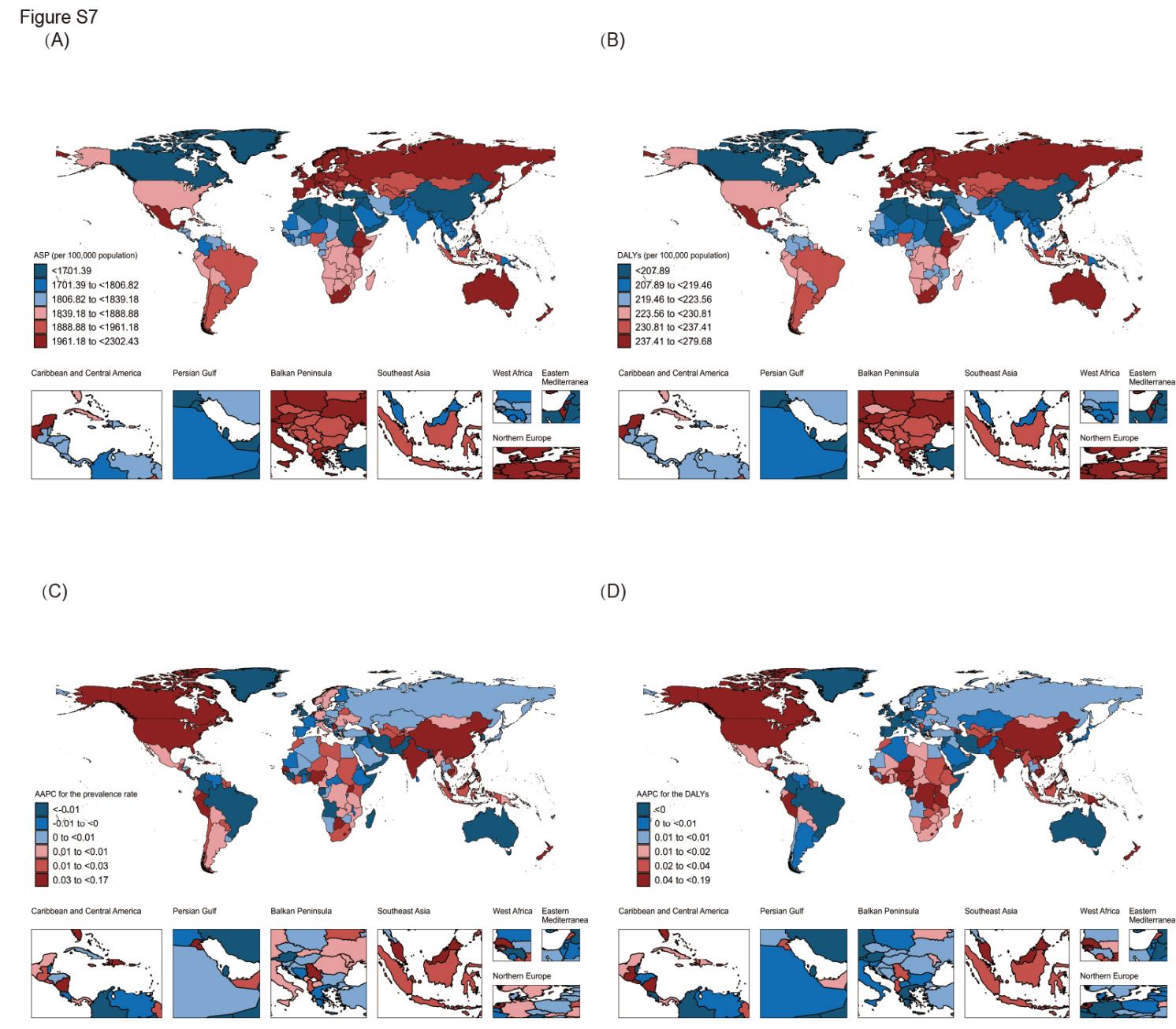


**Supplementary Figure 7.** Global map of (A) age-standardized prevalence (ASP) and (B) age-standardized disability-adjusted life years (DALYs) for eating disorders in individuals aged 10-24 in 2021, and average annual percent change in (C) ASP and (D) DALYs from 1990 to 2021 across 204 countries and territories.


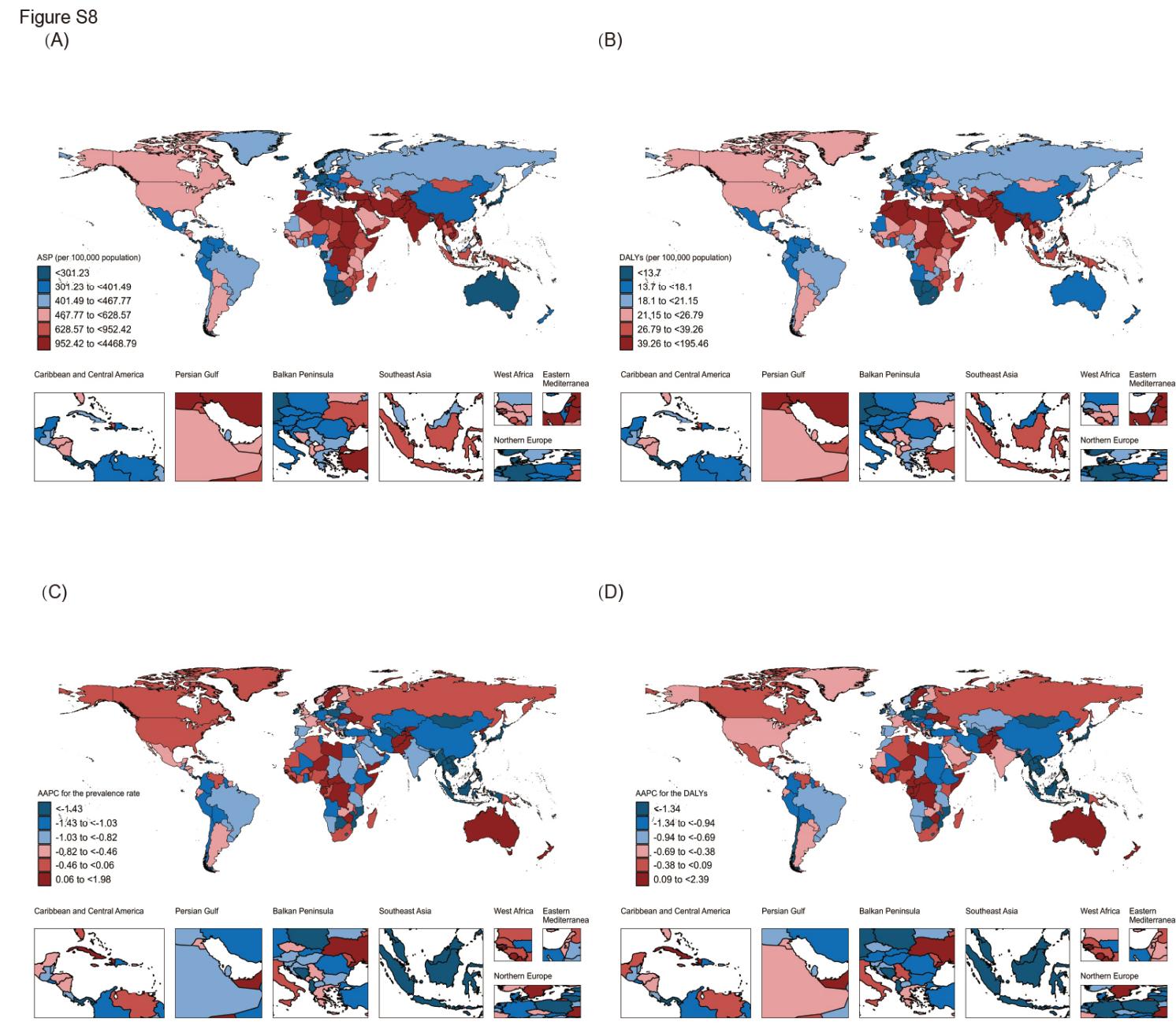


**Supplementary Figure 8.** Global map of (A) age-standardized prevalence (ASP) and (B) age-standardized disability-adjusted life years (DALYs) for idiopathic developmental intellectual disability in individuals aged 10-24 in 2021, and average annual percent change in (C) ASP and (D) DALYs from 1990 to 2021 across 204 countries and territories.


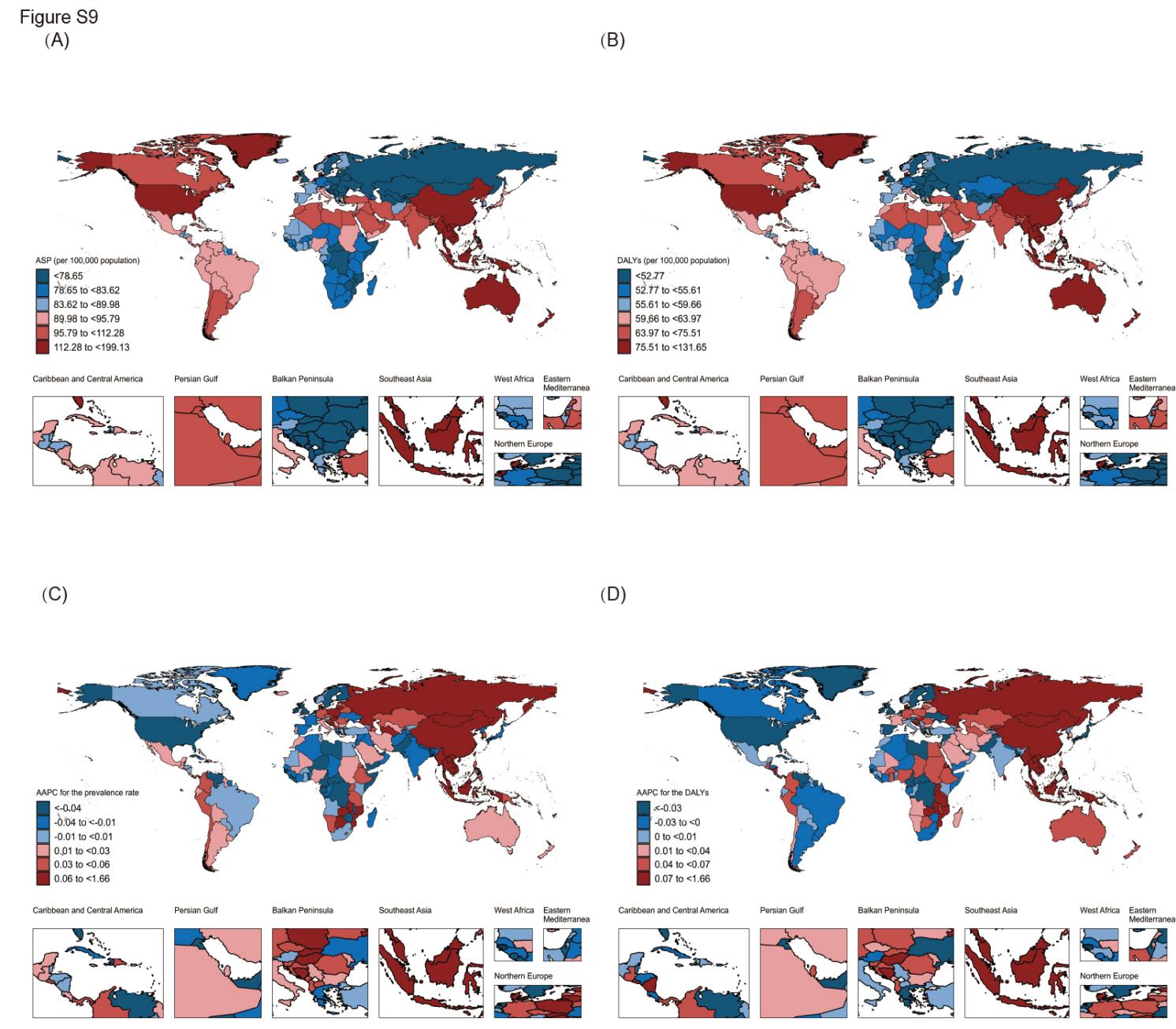


**Supplementary Figure 9.** Global map of (A) age-standardized prevalence (ASP) and (B) age-standardized disability-adjusted life years (DALYs) for schizophrenia in individuals aged 10-24 in 2021, and average annual percent change in (C) ASP and (D) DALYs from 1990 to 2021 across 204 countries and territories.


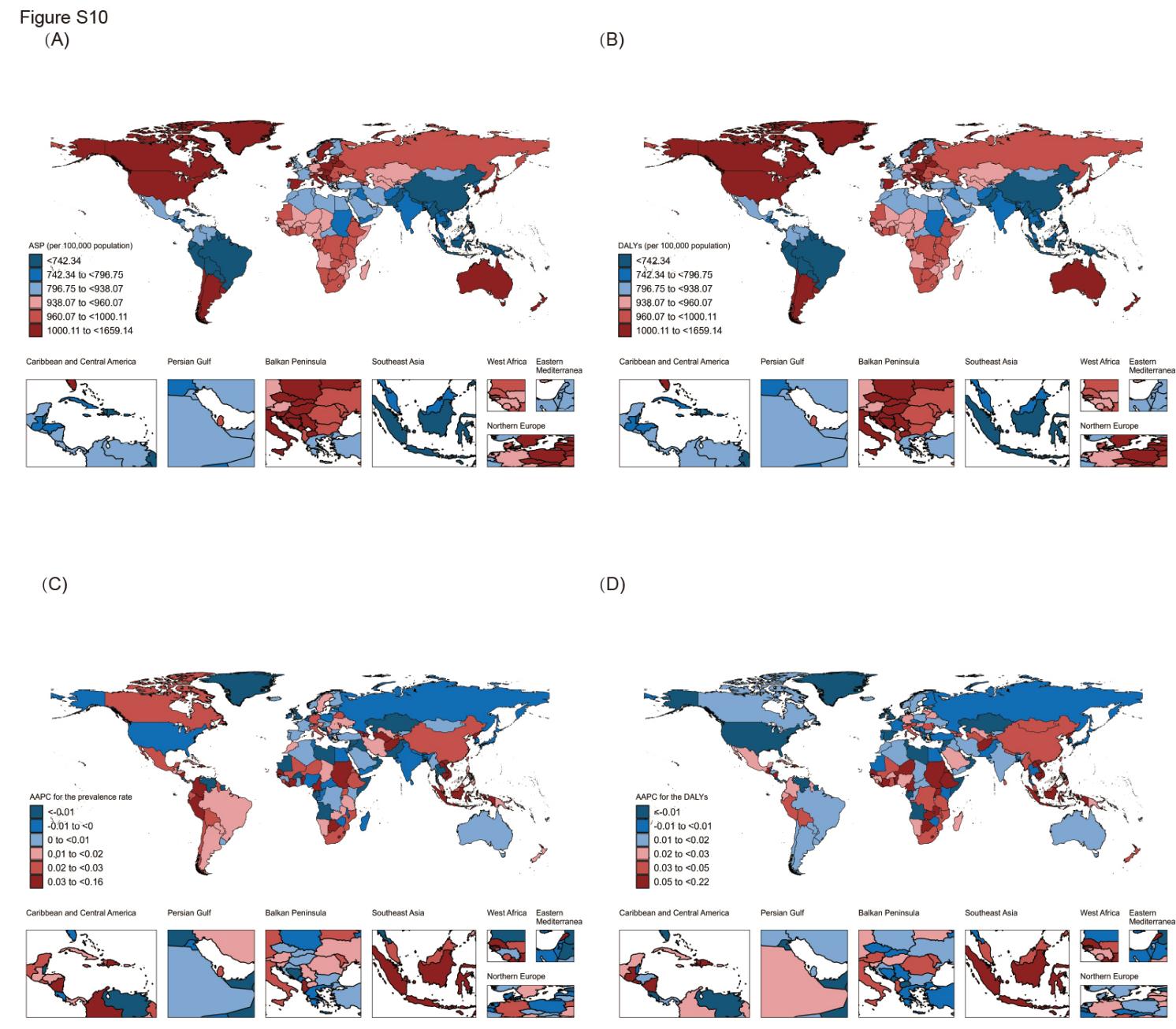


**Supplementary Figure 10.** Global map of (A) age-standardized prevalence (ASP) and (B) age-standardized disability-adjusted life years (DALYs) for other mental disorders in individuals aged 10-24 in 2021, and average annual percent change in (C) ASP and (D) DALYs from 1990 to 2021 across 204 countries and territories.


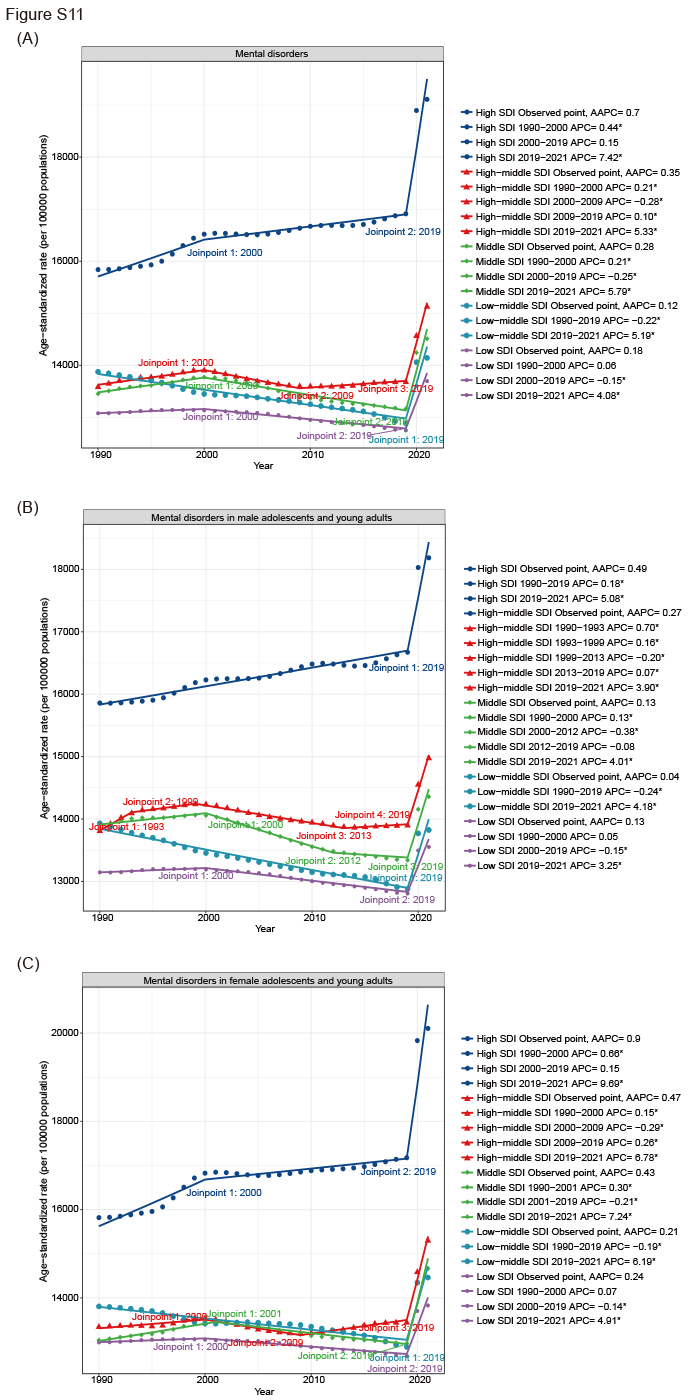


**Supplementary Figure 11.** Temporal trends of age-standardized prevalence (ASP) for ten mental disorders in (A) both sexes, (B) male, and (C) female by SDI regions from 1990 to 2021 globally. APC, annual percent change.


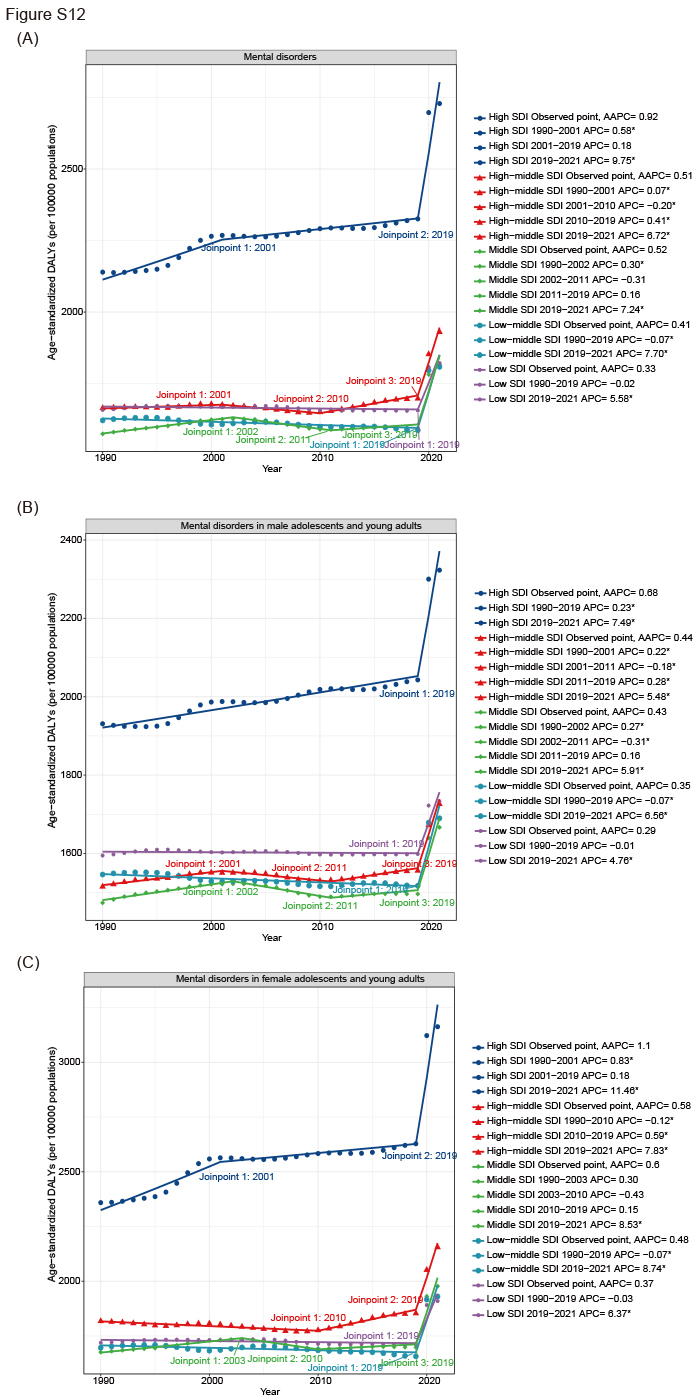


**Supplementary Figure 12.** Temporal trends of age-standardized disability-adjusted life years (DALYs) for ten mental disorders in (A) both sexes, (B) male, and (C) female by SDI regions from 1990 to 2021 globally. APC, annual percent change.


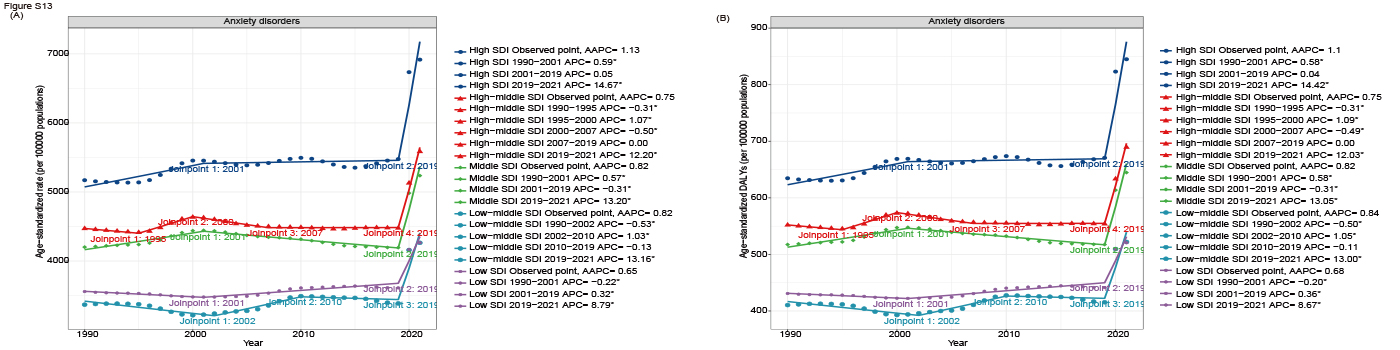


**Supplementary Figure 13.** Temporal trends of (A) age-standardized prevalence (ASP) and (B) age-standardized disability-adjusted life years (DALYs) for anxiety disorders by SDI regions from 1990 to 2021 globally. APC, annual percent change.


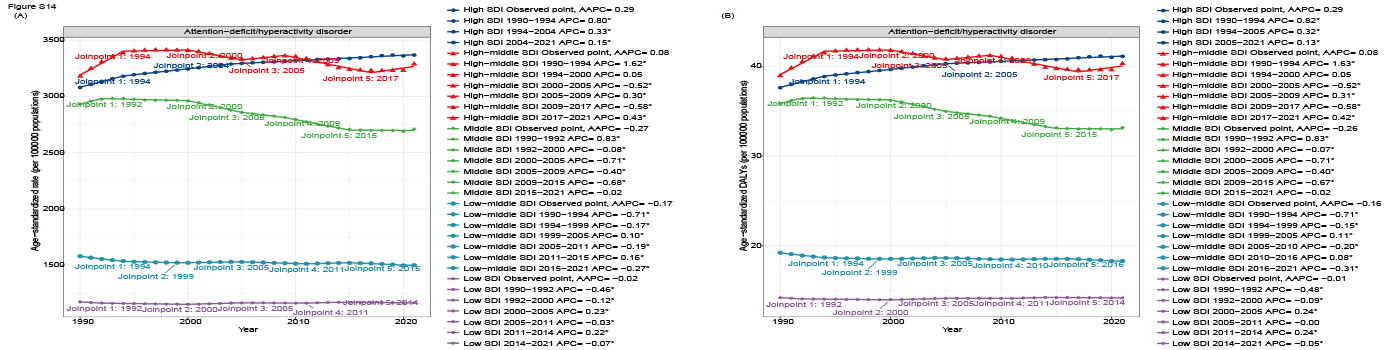


**Supplementary Figure 14.** Temporal trends of (A) age-standardized prevalence (ASP) and (B) age-standardized disability-adjusted life years (DALYs) for attention−deficit/hyperactivity disorder by SDI regions from 1990 to 2021 globally. APC, annual percent change.


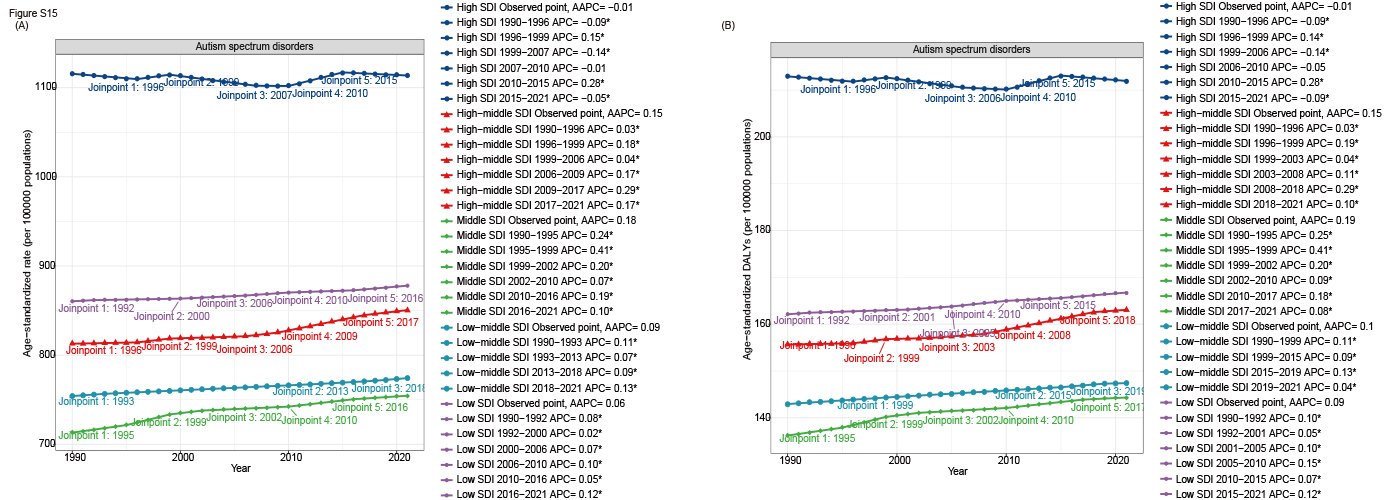


**Supplementary Figure 15.** Temporal trends of (A) age-standardized prevalence (ASP) and (B) age-standardized disability-adjusted life years (DALYs) for autism spectrum disorders by SDI regions from 1990 to 2021 globally. APC, annual percent change.


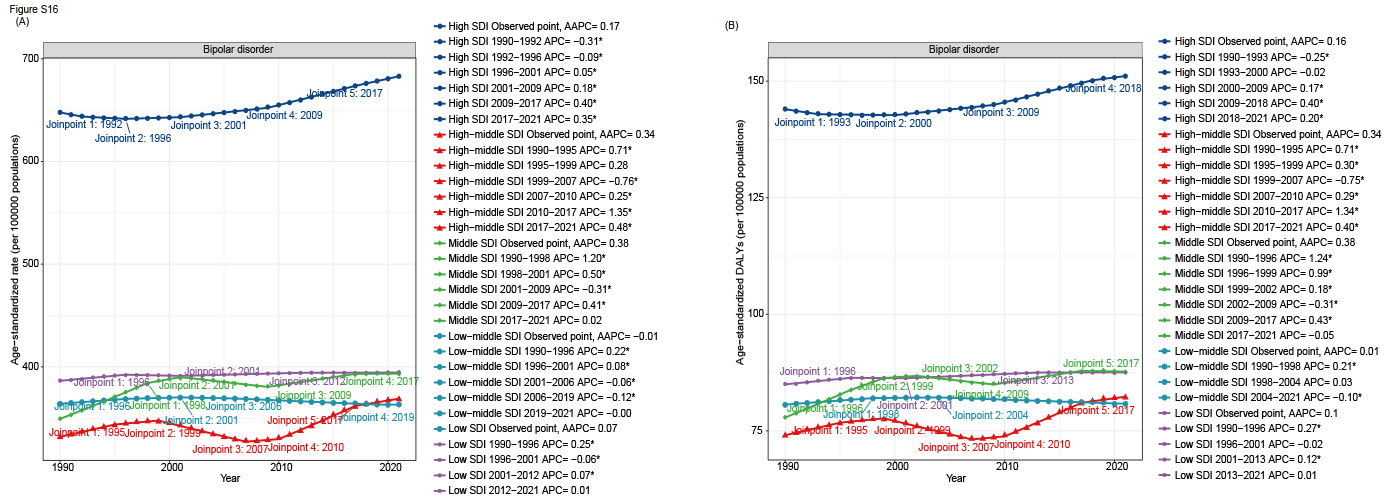


**Supplementary Figure 16.** Temporal trends of (A) age-standardized prevalence (ASP) and (B) age-standardized disability-adjusted life years (DALYs) for bipolar disorder by SDI regions from 1990 to 2021 globally. APC, annual percent change.


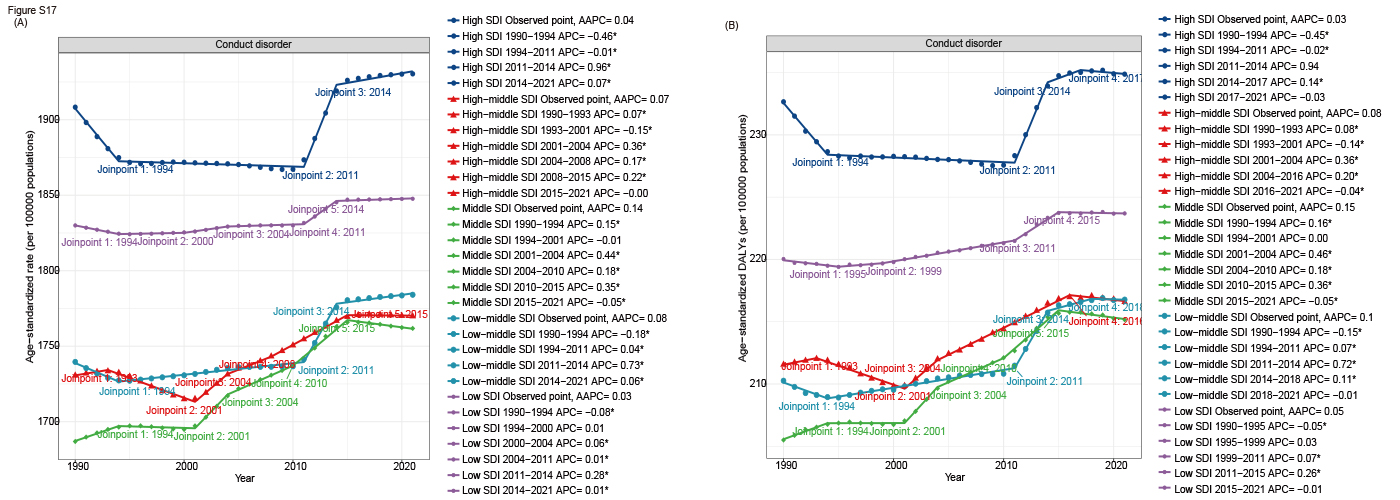


**Supplementary Figure 17.** Temporal trends of (A) age-standardized prevalence (ASP) and (B) age-standardized disability-adjusted life years (DALYs) for conduct disorder by SDI regions from 1990 to 2021 globally. APC, annual percent change.


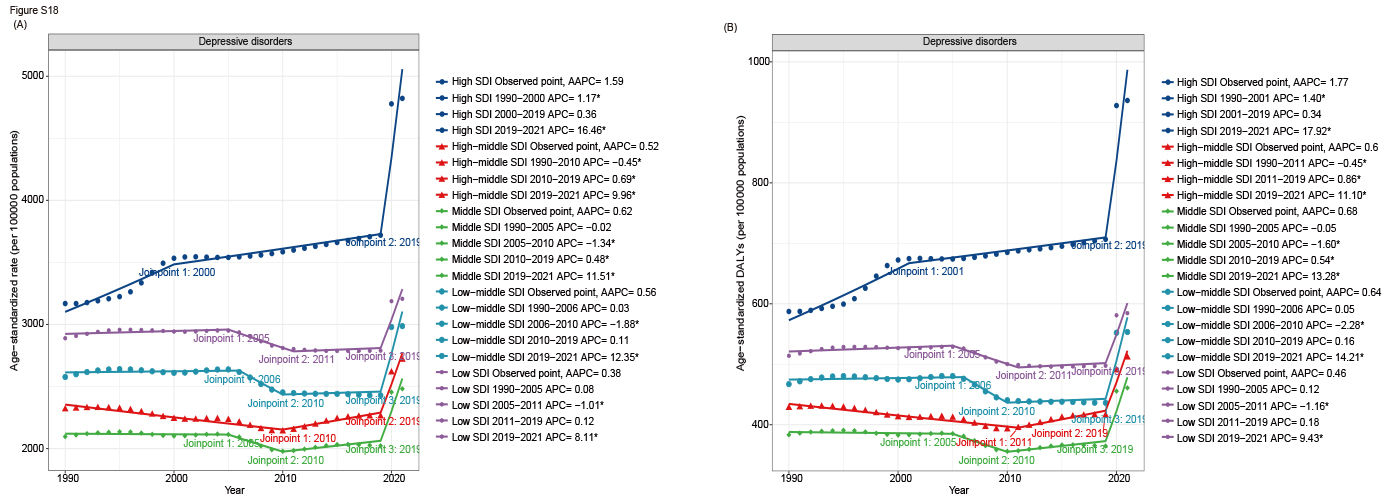


**Supplementary Figure 18.** Temporal trends of (A) age-standardized prevalence (ASP) and (B) age-standardized disability-adjusted life years (DALYs) for depressive disorders by SDI regions from 1990 to 2021 globally. APC, annual percent change.


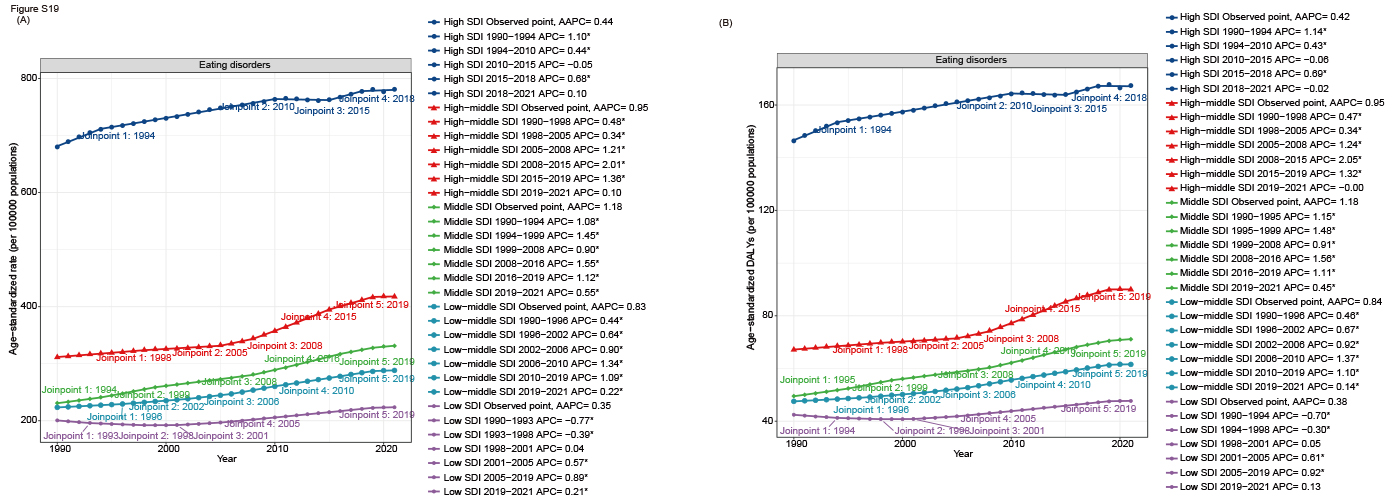


**Supplementary Figure 19.** Temporal trends of (A) age-standardized prevalence (ASP) and (B) age-standardized disability-adjusted life years (DALYs) for eating disorders by SDI regions from 1990 to 2021 globally. APC, annual percent change.


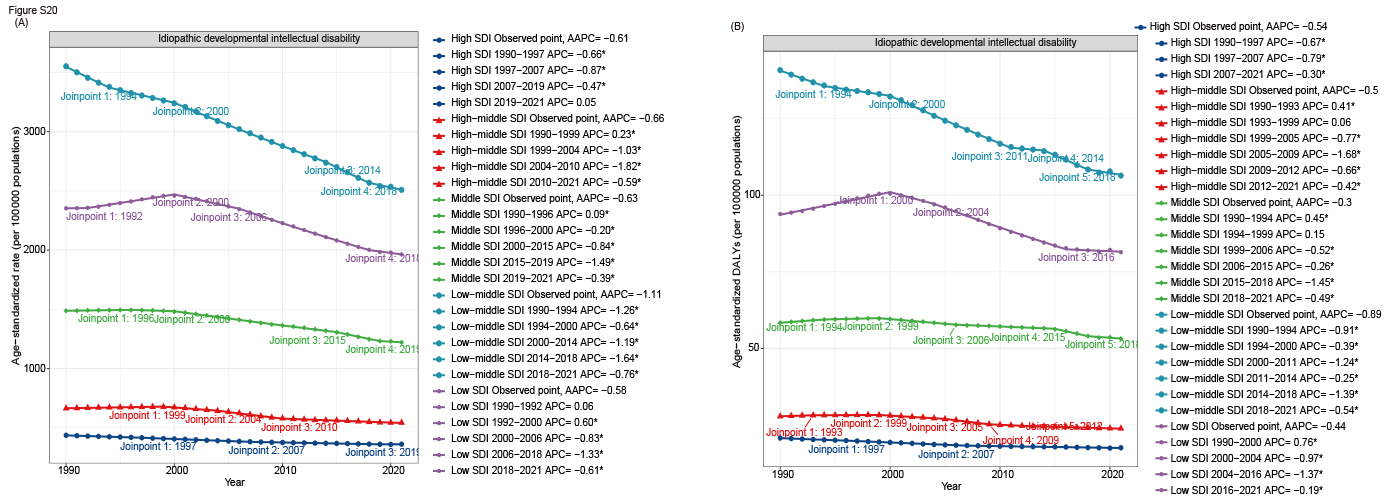


**Supplementary Figure 20.** Temporal trends of (A) age-standardized prevalence (ASP) and (B) age-standardized disability-adjusted life years (DALYs) for idiopathic developmental intellectual disability by SDI regions from 1990 to 2021 globally. APC, annual percent change.


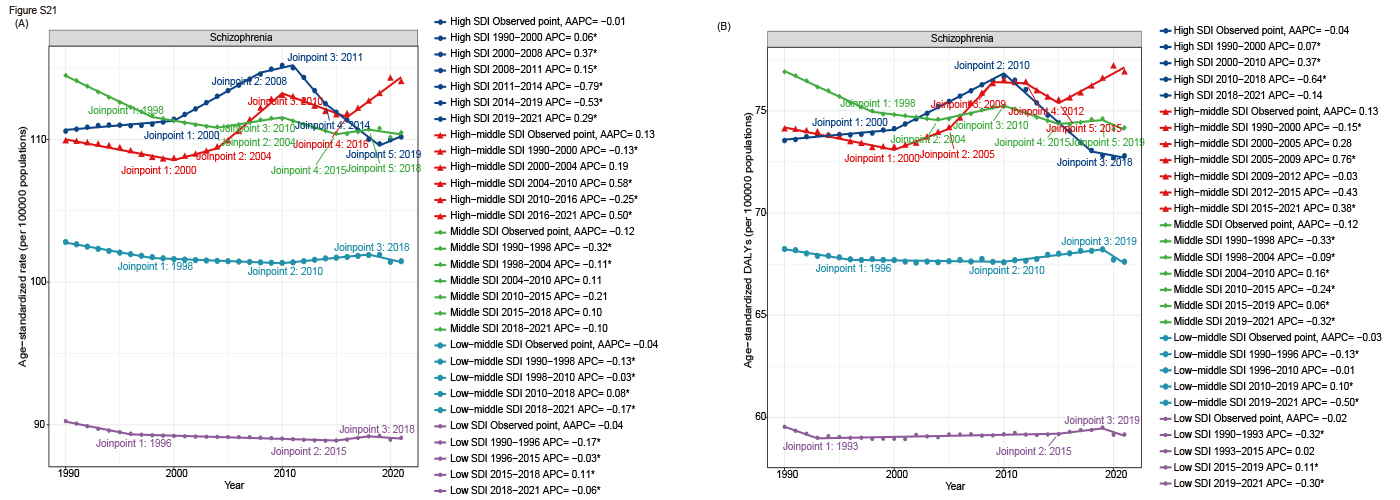


**Supplementary Figure 21.** Temporal trends of (A) age-standardized prevalence (ASP) and (B) age-standardized disability-adjusted life years (DALYs) for schizophrenia by SDI regions from 1990 to 2021 globally. APC, annual percent change.


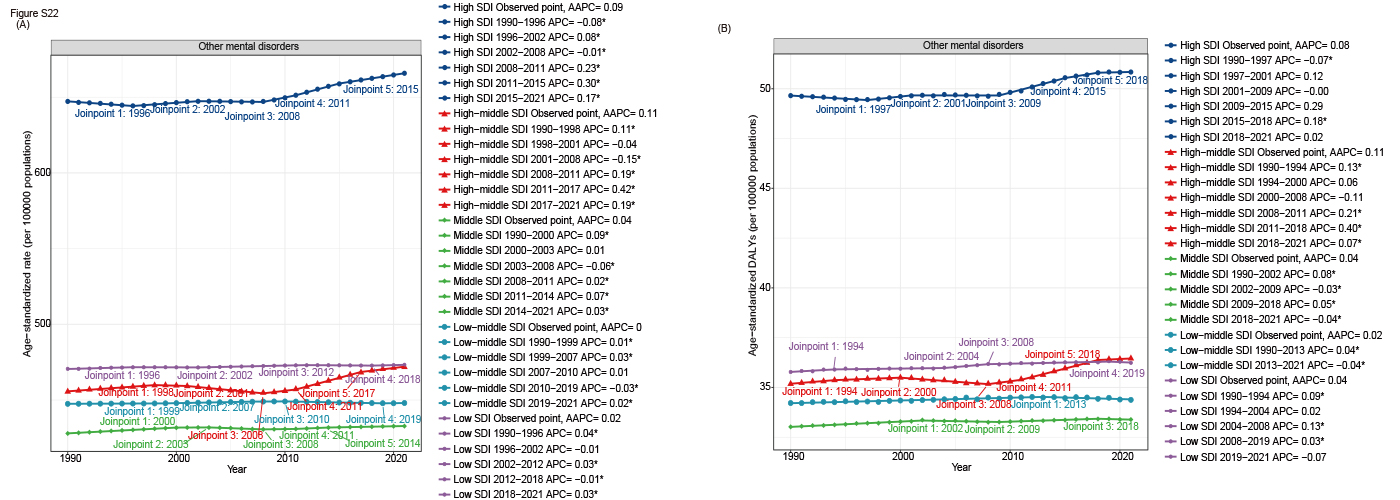


**Supplementary Figure 22.** Temporal trends of (A) age-standardized prevalence (ASP) and (B) age-standardized disability-adjusted life years (DALYs) for other mental disorders by SDI regions from 1990 to 2021 globally. APC, annual percent change.


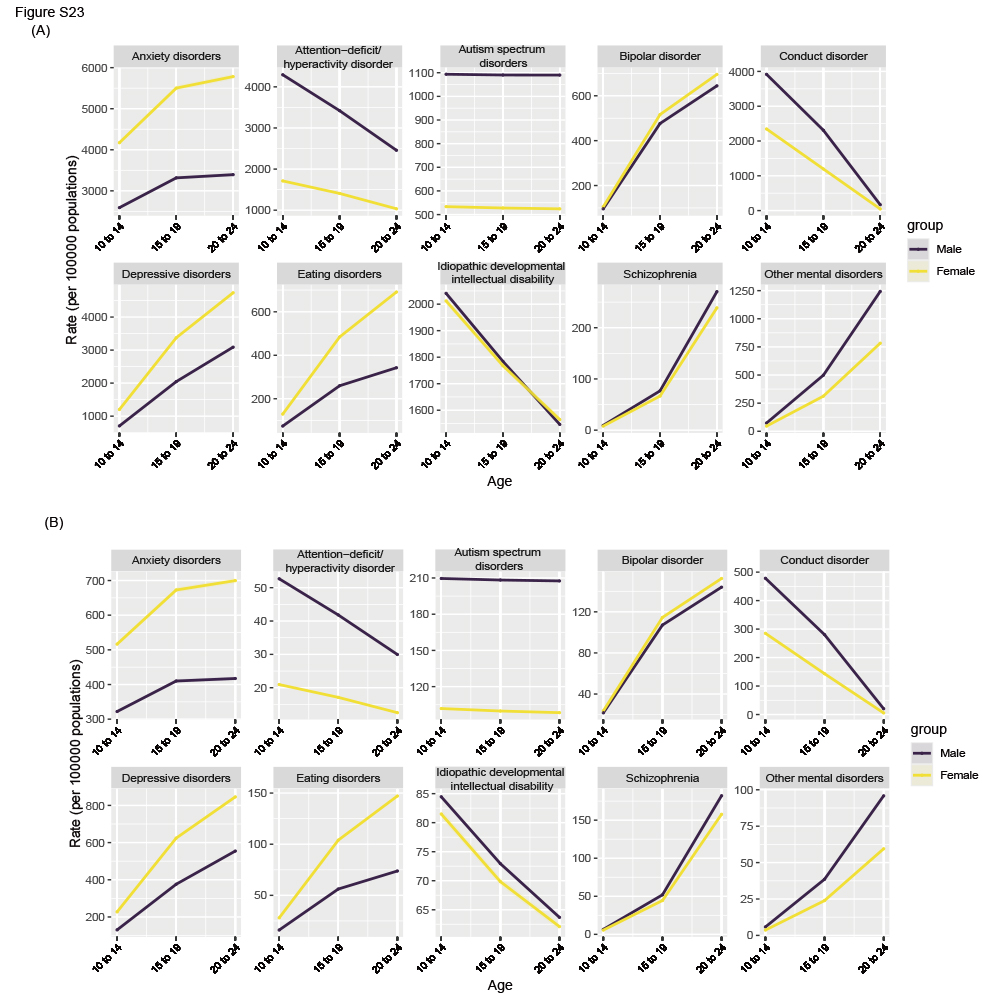


**Supplementary Figure 23.** The effect of age on (A) prevalence and (B) disability-adjusted life years (DALYs) for ten mental disorders and sex difference from 1990 to 2021.


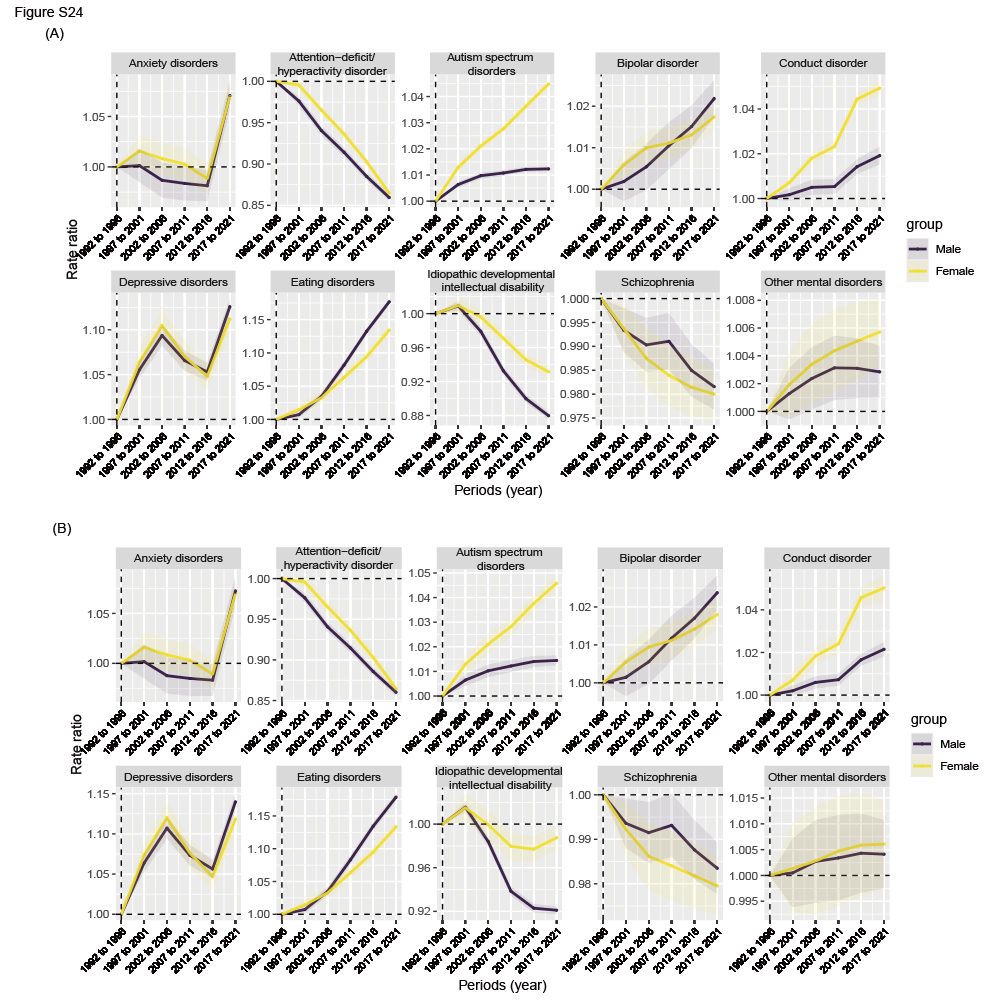


**Supplementary Figure 24.** The effect of period on (A) prevalence and (B) disability-adjusted life years (DALYs) for ten mental disorders and sex difference from 1990 to 2021.


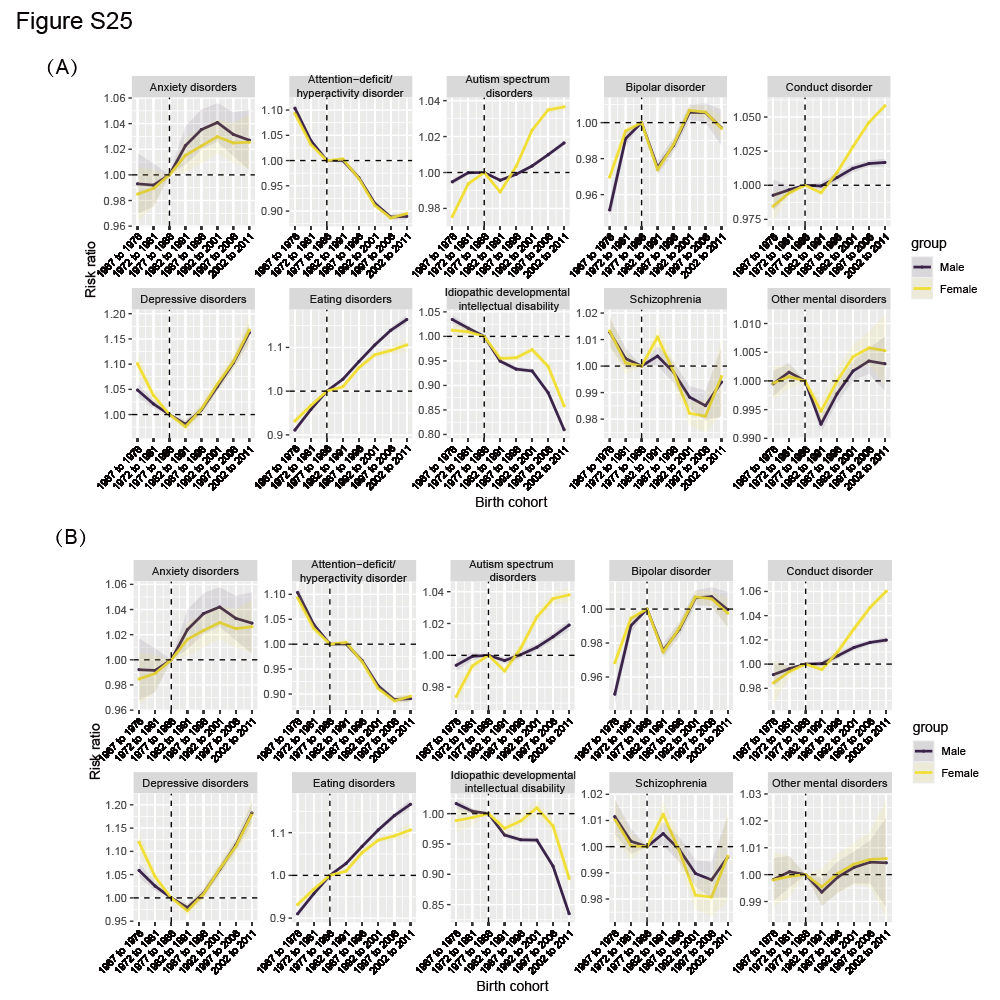


**Supplementary Figure 25.** The effect of cohort on (A) prevalence and (B) disability-adjusted life years (DALYs) for ten mental disorders and sex difference from 1990 to 2021.


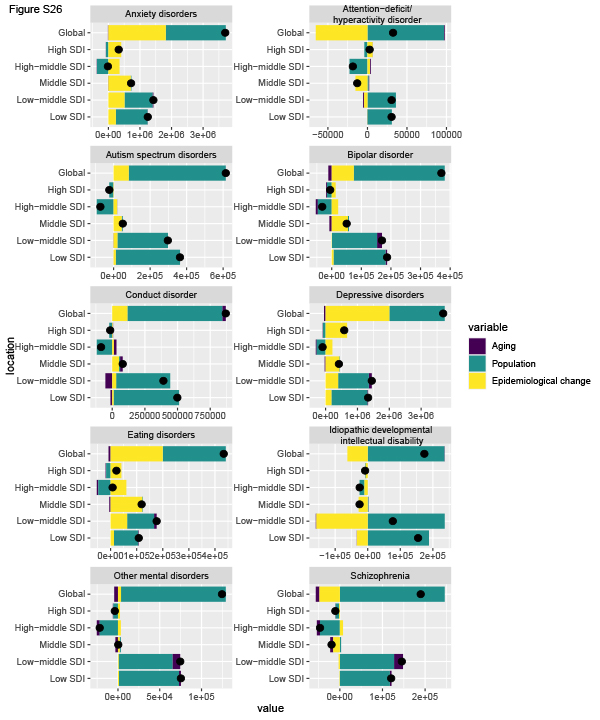


**Supplementary Figure 26.** Contribution of changes in population growth, population aging, and morbidity change to the percentage change in disability-adjusted life years (DALYs) due to ten mental disorders, 2000–2021.

## Supplementary **Table**s

**Table S1.** **Age-standardized prevalence and** **DALYs of mental disorders in adolescents and young adults in 204** **countries and territories, 1990-2021**

|  | **Age-standardized-Prevalence** **per 100,000 population** | | | **Age-standardized-DALYs per 100,000 population** | | |
| --- | --- | --- | --- | --- | --- | --- |
|  | 1990(95% UI) | 2021(95% UI) | AAPC (95% CI) | 1990(95% UI) | 2021(95% UI) | AAPC (95% CI) |
| **Afghanistan** | 18354(15454.2~21583.3) | 20976.1(17154.7~25455.9) | 0.49(0.43~0.57) | 2370.6(1666.7~3233.3) | 2828.3(1900.1~3909.5) | 0.62(0.55~0.72) |
| **Albania** | 11933.5(10084.5~13972.6) | 13840.1(11453.5~16888.4) | 0.51(0.45~0.59) | 1467.8(1046~1977.5) | 1822.1(1257.1~2536.1) | 0.74(0.67~0.85) |
| **Algeria** | 16744.3(14147.2~19811.1) | 17980.7(14868.4~21675.9) | 0.27(0.19~0.35) | 2268.1(1574.3~3099.4) | 2501.3(1684.2~3436.3) | 0.41(0.31~0.53) |
| **American Samoa** | 11893.1(9951.4~14023) | 12861.8(10475~15715.8) | 0.24(0.19~0.26) | 1514.5(1073~2069.9) | 1651.8(1125.6~2293.4) | 0.27(0.21~0.30) |
| **Andorra** | 17886.5(15135.8~21113.5) | 20570.6(16777.7~25045.8) | 0.58(0.45~0.77) | 2514.4(1773.8~3420.3) | 2982.9(2042.1~4225.5) | 0.64(0.46~0.79) |
| **Angola** | 13449.8(11246.3~15958.7) | 14441.9(11794.2~17492.6) | 0.27(0.22~0.35) | 2023.2(1407.2~2786.4) | 2230.9(1507.2~3176.4) | 0.38(0.32~0.49) |
| **Antigua and Barbuda** | 16370.4(13855.3~19387.1) | 18076.3(14917.9~21621.7) | 0.34(0.32~0.36) | 1769.5(1253.4~2395.3) | 2064.1(1416.8~2873.9) | 0.53(0.51~0.56) |
| **Argentina** | 15980.3(13525.2~18766.1) | 18514.4(15329.5~22424.3) | 0.57(0.44~0.69) | 2175.3(1546.5~2956) | 2626.4(1814.1~3622.1) | 0.68(0.55~0.84) |
| **Armenia** | 11172.1(9588.5~12917.5) | 13097.3(10876.3~15732.7) | 0.57(0.51~0.65) | 1388.1(984.8~1850.4) | 1766(1217~2437.7) | 0.90(0.82~1.02) |
| **Australia** | 22842(19748.8~26259.1) | 24324.4(20978.3~28136.2) | 0.23(0.21~0.26) | 3076.5(2198.5~4091.7) | 3369.1(2409.7~4527.6) | 0.33(0.30~0.37) |
| **Austria** | 18236.9(15390.7~21516.9) | 20507.4(16824.7~24745.7) | 0.44(0.35~0.55) | 2461.1(1728.3~3355) | 2820.7(1906~3975.1) | 0.48(0.36~0.57) |
| **Azerbaijan** | 10690.2(9201.3~12364.7) | 12142.9(10213.7~14274.2) | 0.46(0.42~0.50) | 1337.7(954.4~1796.8) | 1610.1(1124.4~2208.5) | 0.66(0.62~0.72) |
| **Bahamas** | 16401.8(13869.6~19355.2) | 18365.4(15214.6~21956.9) | 0.43(0.38~0.50) | 1793.8(1270.3~2416.8) | 2125.6(1469.3~2879.4) | 0.65(0.57~0.77) |
| **Bahrain** | 17370.4(14580.9~20552.6) | 19014.9(15509.5~23149.6) | 0.31(0.25~0.39) | 2503.5(1733.7~3438) | 2804.8(1885~3902.8) | 0.43(0.36~0.54) |
| **Bangladesh** | 11405.7(9589.9~13441.3) | 11363.6(9383.9~13605.3) | 0.06(-0.01~0.17) | 1535.4(1070.3~2104.5) | 1614.2(1094.1~2250.7) | 0.20(0.07~0.29) |
| **Barbados** | 16440.6(13913.4~19442) | 18575.1(15495.8~22513.5) | 0.39(0.37~0.40) | 1793.3(1275.2~2420.4) | 2154.5(1491.7~3004.2) | 0.59(0.57~0.6) |
| **Belarus** | 12663.1(10763.8~14828.6) | 16035.4(13031.2~19479.5) | 0.77(0.76~0.79) | 1666.8(1185.3~2255.9) | 2252.3(1542.1~3088.2) | 0.98(0.97~1.00) |
| **Belgium** | 17168.6(14558.2~20020.7) | 19440.2(16059.3~23901.8) | 0.54(0.40~0.75) | 2356.7(1672.6~3196.8) | 2774.4(1913.3~3819.7) | 0.63(0.45~0.81) |
| **Belize** | 16520.4(13954.6~19517.1) | 18068(14978.9~21498.9) | 0.35(0.29~0.44) | 1769.3(1255.6~2387.8) | 2061.6(1450.5~2788.5) | 0.57(0.48~0.71) |
| **Benin** | 11559.5(9725.6~13572.6) | 12191.7(10042.6~14827.1) | 0.19(0.17~0.21) | 1593.9(1119.4~2154.7) | 1724.8(1181.6~2425.6) | 0.28(0.25~0.31) |
| **Bermuda** | 16880(14338~19832.2) | 18528.8(15463.5~21951.6) | 0.32(0.30~0.34) | 1919.9(1360.9~2614.9) | 2198.1(1496.5~3052.5) | 0.47(0.44~0.52) |
| **Bhutan** | 11629.9(9878.2~13590) | 10746.5(9015.6~12698.3) | -0.22(-0.24~-0.17) | 1391.9(981~1864.4) | 1379.2(955.6~1892.8) | 0.04(0.00~0.1) |
| **Bolivia (Plurinational State of)** | 16224.3(13654.5~19126.3) | 19708.9(16203.1~24066.8) | 0.79(0.61~1.04) | 1909.5(1350.3~2601.1) | 2501.4(1715.4~3472) | 1.10(0.85~1.44) |
| **Bosnia and Herzegovina** | 12927.4(10948.6~15015.8) | 13937.8(11541.4~16799.2) | 0.27(0.24~0.31) | 1638(1174.8~2203.6) | 1863.4(1283.9~2552.1) | 0.45(0.41~0.50) |
| **Botswana** | 11611.5(9785.9~13675.5) | 14053.3(11396.1~17162.1) | 0.53(0.37~0.59) | 1652.6(1169.6~2237.5) | 2089.4(1435~2878.5) | 0.66(0.48~0.73) |
| **Brazil** | 16783.2(14641.2~19258.5) | 20058.2(17243.4~23074.4) | 0.65(0.59~0.74) | 2087.6(1491.4~2792) | 2594.5(1824.7~3489.2) | 0.77(0.69~0.90) |
| **Brunei Darussalam** | 12919.8(11091.5~14917) | 13760.2(11570.2~16351.6) | 0.22(0.21~0.24) | 1728.6(1249~2306.1) | 1864.1(1314.4~2540.2) | 0.27(0.26~0.30) |
| **Bulgaria** | 11853.5(10107.8~13855.6) | 14717.6(11969.2~17902.7) | 0.69(0.69~0.70) | 1513.4(1073.8~2039.2) | 1977.7(1353.6~2742.5) | 0.86(0.85~0.87) |
| **Burkina Faso** | 11668.7(9911.8~13730.1) | 12061.6(9907.7~14650.4) | 0.13(0.10~0.16) | 1596.7(1124.8~2158.1) | 1674.8(1172.1~2274.9) | 0.19(0.14~0.23) |
| **Burundi** | 12512.5(10633.5~14611.4) | 13771.5(11357.7~16523.1) | 0.33(0.31~0.36) | 1762(1228.1~2398.8) | 1931.7(1317.6~2663.7) | 0.31(0.29~0.35) |
| **Cabo Verde** | 11644.5(9824.7~13628.7) | 13242(10922.2~15971) | 0.47(0.40~0.57) | 1652.8(1161.5~2244.6) | 1979.2(1365.8~2731) | 0.64(0.57~0.75) |
| **Cambodia** | 13029.5(10941~15316.5) | 13611.4(11178.5~16479.7) | 0.10(0.04~0.15) | 1593.3(1124.1~2173.6) | 1801.4(1230.6~2514) | 0.32(0.24~0.35) |
| **Cameroon** | 11559.1(9743.7~13634.7) | 12276.3(10027.7~14875) | 0.22(0.20~0.24) | 1617.2(1137.3~2195.4) | 1747(1193.8~2405.3) | 0.28(0.26~0.32) |
| **Canada** | 15946.7(14172.9~17748.2) | 17999.6(15134.2~21136.6) | 0.44(0.38~0.51) | 2148.6(1559.5~2821.2) | 2547.4(1792.5~3429.3) | 0.61(0.54~0.72) |
| **Central African Republic** | 13800.6(11498.1~16257.6) | 14750.9(12003.4~17994.1) | 0.30(0.21~0.41) | 2015.9(1406.3~2749.5) | 2164(1463.6~3034.1) | 0.32(0.21~0.47) |
| **Chad** | 12236.6(10335.9~14398.4) | 12502.9(10433.1~15171.7) | 0.13(0.08~0.21) | 1700.7(1194.2~2332.2) | 1778.8(1224~2435) | 0.23(0.16~0.33) |
| **Chile** | 17863.6(15974.2~19882.3) | 20111.6(16683.6~24513.2) | 0.49(0.37~0.66) | 2606.6(1858.1~3449.8) | 3008.9(2047~4194.3) | 0.59(0.44~0.81) |
| **China** | 12827.7(11008.5~14793.5) | 13002.6(11069.6~15049.3) | -0.01(-0.07~0.03) | 1413.9(1016.5~1879.8) | 1371.4(974.5~1823.4) | -0.15(-0.2~-0.11) |
| **Colombia** | 14163.7(11930.5~16623.4) | 15608.6(12763.2~19149.3) | 0.34(0.23~0.43) | 1732.4(1227.4~2346) | 1977.1(1346.4~2745.4) | 0.52(0.38~0.63) |
| **Comoros** | 11603.8(9846.5~13595.3) | 12876.6(10694.5~15609.6) | 0.36(0.34~0.39) | 1633(1143.1~2185.8) | 1842.4(1265.9~2536.7) | 0.42(0.39~0.46) |
| **Congo** | 13216.7(11072.9~15737.3) | 14206.6(11633.3~17273.5) | 0.32(0.26~0.42) | 1994.6(1389.1~2749.1) | 2175.1(1483.4~3048.6) | 0.34(0.23~0.43) |
| **Cook Islands** | 12142.6(10165.3~14356.2) | 13252.2(10878.4~16077.9) | 0.26(0.20~0.28) | 1575.3(1094~2163.2) | 1771.9(1202~2502.7) | 0.35(0.29~0.38) |
| **Costa Rica** | 13949.3(11857.3~16343.9) | 15946.6(13214.8~19217.4) | 0.49(0.43~0.56) | 1752.7(1253.9~2377.4) | 2109.3(1450.7~2933) | 0.67(0.60~0.76) |
| **Coted'Ivoire** | 11245.8(9460.6~13234.9) | 11683(9703.9~14058) | 0.15(0.12~0.20) | 1541.6(1092~2074.7) | 1624.9(1130.2~2221.3) | 0.19(0.15~0.24) |
| **Croatia** | 12052.8(10265.9~14020.4) | 13532.4(11206.6~16322.7) | 0.41(0.37~0.46) | 1588.8(1140~2133.4) | 1827.1(1269.3~2527.1) | 0.47(0.43~0.54) |
| **Cuba** | 17655.9(14952.5~20700.5) | 18690(15606~22502.8) | 0.17(0.15~0.19) | 2057.1(1467.9~2795.1) | 2164.5(1509.5~2965.1) | 0.15(0.12~0.17) |
| **Cyprus** | 18004.8(15174.7~21315.2) | 20099.3(16344.1~24606.6) | 0.39(0.37~0.43) | 2455.4(1712.3~3351.7) | 2826.7(1892.4~3991.2) | 0.51(0.47~0.55) |
| **Czechia** | 11721.9(10015.8~13665.8) | 13356.4(10974.2~16047) | 0.43(0.40~0.48) | 1534.4(1094.5~2056) | 1814(1243.2~2487) | 0.54(0.51~0.59) |
| **Democratic People's Republic of Korea** | 11980.1(9982.7~14164.6) | 12599.6(10444.7~15241.3) | 0.16(0.16~0.17) | 1409.1(988~1930.7) | 1442.3(994.8~1977.4) | 0.08(0.07~0.08) |
| **Democratic Republic of the Congo** | 13284.3(11078.6~15744.3) | 14235.6(11727.9~17160) | 0.27(0.20~0.38) | 1936.5(1345.6~2646.4) | 2061(1442.8~2901.4) | 0.29(0.20~0.40) |
| **Denmark** | 17330.9(14736.4~20371.9) | 19046.8(15699.9~23020.5) | 0.36(0.30~0.44) | 2443.2(1732.5~3307.2) | 2758.1(1908.2~3720.4) | 0.44(0.37~0.52) |
| **Djibouti** | 11595.5(9876.4~13547.8) | 12661.9(10531.8~15189.6) | 0.33(0.29~0.40) | 1648(1156.8~2232.5) | 1834.9(1265.8~2514.3) | 0.41(0.35~0.48) |
| **Dominica** | 16469.3(13913.9~19465.4) | 18296.9(15192.5~21857.5) | 0.35(0.34~0.35) | 1750.9(1242.6~2353.4) | 2075.9(1436.6~2842.7) | 0.56(0.55~0.58) |
| **Dominican Republic** | 16850.4(14209.9~19940.4) | 18141.9(15077.1~21750.5) | 0.29(0.20~0.39) | 1858.1(1314.2~2560.2) | 2095(1461.7~2925.9) | 0.48(0.34~0.63) |
| **Ecuador** | 15979.3(13500.8~18817.7) | 18599.9(15293.6~22416.1) | 0.65(0.50~0.86) | 1936.8(1363.3~2629.1) | 2395.4(1631.5~3330) | 0.81(0.57~1.01) |
| **Egypt** | 16908.8(14375.8~19807.7) | 18268.5(14841.2~21983.4) | 0.29(0.19~0.37) | 2137.6(1487.1~2911.2) | 2504.4(1683.9~3444.9) | 0.57(0.48~0.68) |
| **El Salvador** | 14965.7(12600.9~17454.1) | 16230.1(13532.9~19610.7) | 0.35(0.25~0.49) | 1919.3(1372.9~2565) | 2147.1(1487.2~2963.3) | 0.45(0.33~0.63) |
| **Equatorial Guinea** | 13515.2(11298.9~16001.4) | 13962(11359~17177.4) | 0.16(0.08~0.26) | 2007(1390.9~2744.3) | 2169.3(1489~3091.3) | 0.36(0.28~0.49) |
| **Eritrea** | 12344.7(10409.7~14454.6) | 12999.8(10779.1~15517.1) | 0.16(0.14~0.17) | 1692.3(1190.8~2298.4) | 1855.5(1281.7~2580.7) | 0.30(0.28~0.31) |
| **Estonia** | 13025.5(11108.8~15139.1) | 14778.2(12151.9~18002.6) | 0.37(0.30~0.41) | 1772.4(1259.9~2390.3) | 2058.2(1417.1~2884.7) | 0.44(0.35~0.49) |
| **Eswatini** | 11710.6(9880.3~13767.9) | 15405.4(12510.7~18848.5) | 0.88(0.86~0.89) | 1667.2(1161.5~2266.2) | 2330.4(1603~3256.5) | 1.08(1.06~1.09) |
| **Ethiopia** | 12585.9(10899.4~14365.6) | 12941.4(11190.6~14824.8) | 0.10(0.06~0.14) | 1740.2(1246.2~2322.3) | 1840.1(1310.2~2461) | 0.21(0.16~0.27) |
| **Fiji** | 12201.9(10246.1~14352.9) | 13639.6(11087.1~16570.7) | 0.33(0.26~0.36) | 1527.8(1083.1~2084.3) | 1791.1(1227.8~2529.2) | 0.47(0.38~0.51) |
| **Finland** | 19829.7(16940.1~22795.6) | 19901.5(16759~23717.5) | 0.06(0.03~0.10) | 2773.1(1959~3758.3) | 2882.9(1991~3927.8) | 0.15(0.11~0.20) |
| **France** | 19775.5(16931.5~22935) | 21873.1(17797.8~26627) | 0.48(0.38~0.63) | 2778.1(1944.9~3733.4) | 3103.3(2109.7~4295.8) | 0.45(0.3~0.58) |
| **Gabon** | 13145.8(11018.8~15656.4) | 14665.6(11971.8~17985.4) | 0.38(0.36~0.41) | 2008.2(1380.7~2761.4) | 2277(1543.8~3260) | 0.44(0.42~0.48) |
| **Gambia** | 12313.7(10278.3~14487.9) | 13305.4(10998.6~16234.3) | 0.31(0.25~0.38) | 1790.7(1251.5~2469.6) | 1950.7(1329.9~2708.6) | 0.33(0.25~0.40) |
| **Georgia** | 11000.3(9483.8~12726.3) | 12781(10635~15224.5) | 0.48(0.47~0.49) | 1406.3(995.9~1894.8) | 1729.3(1186.3~2373.4) | 0.67(0.66~0.67) |
| **Germany** | 16296.6(13868~19207.9) | 19900.1(16265~24486.1) | 0.67(0.64~0.71) | 2368.9(1670.8~3202.4) | 2980.5(2015.4~4151.2) | 0.77(0.74~0.82) |
| **Ghana** | 11476.9(9671~13543) | 12169(9995.4~14696.9) | 0.23(0.19~0.27) | 1597.5(1112.6~2174.2) | 1747.6(1200~2388) | 0.33(0.29~0.38) |
| **Greece** | 19052.4(16075.3~22576.1) | 22625(18461.5~27652.4) | 0.59(0.54~0.63) | 2752(1919.3~3798.3) | 3405.3(2279.6~4785.3) | 0.73(0.68~0.78) |
| **Greenland** | 20357.5(17261.2~23854.9) | 23615.1(19615.7~28600.7) | 0.51(0.42~0.63) | 3104.9(2180~4279.3) | 3791(2612.6~5383.7) | 0.68(0.57~0.84) |
| **Grenada** | 16562.5(14021.4~19559.4) | 18385.8(15290.2~22253.8) | 0.34(0.34~0.35) | 1773.7(1243.2~2382.1) | 2105.2(1422.4~2928) | 0.57(0.55~0.58) |
| **Guam** | 12347(10403.2~14559.4) | 14118.5(11605.3~17126.7) | 0.47(0.42~0.52) | 1643.6(1171.3~2261.4) | 1951.5(1335.7~2725.1) | 0.60(0.54~0.66) |
| **Guatemala** | 14220.5(12014.1~16686.5) | 16118.5(13357.8~19557.2) | 0.48(0.39~0.59) | 1757.3(1239.4~2385.4) | 2111.2(1469.1~2938.3) | 0.67(0.56~0.82) |
| **Guinea** | 11573.8(9731~13656.2) | 12538.4(10328.4~15161.3) | 0.29(0.25~0.34) | 1597.7(1128.8~2180.5) | 1768.8(1230.9~2463.4) | 0.38(0.33~0.45) |
| **Guinea-Bissau** | 11473(9638.9~13481.8) | 12396.7(10283~14973.6) | 0.31(0.26~0.39) | 1578.9(1110.6~2139.2) | 1735.7(1189.2~2420.9) | 0.35(0.27~0.41) |
| **Guyana** | 17671.3(14878.1~20721.3) | 20697.2(17233.7~24688.7) | 0.53(0.52~0.55) | 2009.8(1422.9~2733.8) | 2615(1792.4~3674.7) | 0.89(0.85~0.92) |
| **Haiti** | 17020.1(14404.4~20145.8) | 18595.9(15314.5~22287.7) | 0.32(0.29~0.36) | 1825.2(1297.1~2508.1) | 2071.1(1426.8~2860.4) | 0.45(0.40~0.52) |
| **Honduras** | 13841(11704.1~16315.9) | 16250.3(13314~19648.9) | 0.60(0.52~0.69) | 1677.5(1191.7~2254.5) | 2087.4(1406.3~2901.7) | 0.82(0.72~0.95) |
| **Hungary** | 11891.6(10127.2~13863.9) | 13280.1(10993~15920) | 0.36(0.35~0.36) | 1550.5(1106.4~2085.5) | 1770.9(1227.4~2407.2) | 0.43(0.42~0.44) |
| **Iceland** | 17822.9(15009.1~21140.5) | 18662.1(15272.8~22574.6) | 0.18(0.13~0.23) | 2412.4(1684.7~3256.8) | 2553.3(1756.9~3547.2) | 0.25(0.19~0.32) |
| **India** | 14523.1(12219.4~16902.8) | 14083.8(12105.4~16262.3) | -0.07(-0.12~0.00) | 1510.6(1092~2000) | 1656.3(1180.9~2204.5) | 0.31(0.21~0.40) |
| **Indonesia** | 11358.4(9866.3~12971.9) | 12389.7(10710.5~14248.6) | 0.29(0.28~0.29) | 1355(972.7~1801.9) | 1610.2(1143.5~2169.6) | 0.57(0.56~0.58) |
| **Iran (Islamic Republic of)** | 21983.6(19203.7~25108.9) | 24525.7(21325.6~28025.8) | 0.45(0.38~0.57) | 2699.7(1915.9~3624.8) | 3210.9(2256~4329.8) | 0.76(0.64~0.95) |
| **Iraq** | 16626.7(13950~19582.2) | 17923.7(14672.4~21789.2) | 0.34(0.20~0.54) | 2190.2(1509.2~2986.2) | 2453.1(1669.6~3399.9) | 0.53(0.38~0.75) |
| **Ireland** | 20020.7(16899.9~23615.2) | 23619.3(19252.1~28927.9) | 0.56(0.46~0.62) | 2724.2(1920.2~3700.8) | 3425.5(2359.4~4856.1) | 0.78(0.68~0.88) |
| **Israel** | 16728.2(14295.3~19471.4) | 18154.4(15148.6~21704) | 0.30(0.22~0.39) | 2339.8(1637.6~3180.6) | 2613.3(1803~3672.1) | 0.41(0.31~0.52) |
| **Italy** | 17801.3(15475.6~20205.6) | 20427.8(17754.7~23336.5) | 0.56(0.44~0.71) | 2543(1807.2~3390.4) | 2985.8(2088.2~3982.3) | 0.64(0.49~0.84) |
| **Jamaica** | 16230.8(13705.7~19163.4) | 18285.6(14971.6~21896) | 0.38(0.37~0.39) | 1735.5(1231.6~2350.6) | 2081.7(1408.6~2930.1) | 0.59(0.58~0.59) |
| **Japan** | 13031.4(11415.6~14708.1) | 14534.9(12693.1~16408.1) | 0.35(0.34~0.36) | 1743.3(1275~2280.8) | 2042.2(1490.1~2679.1) | 0.51(0.50~0.52) |
| **Jordan** | 17050.1(14428.9~20041.4) | 18336.8(14922.6~22201.7) | 0.26(0.23~0.31) | 2297.5(1609.1~3150.3) | 2555.1(1716.2~3597.8) | 0.39(0.34~0.45) |
| **Kazakhstan** | 11029.3(9528.4~12692.9) | 12029.5(10104.9~14091.8) | 0.30(0.28~0.33) | 1446.4(1033.2~1955.9) | 1659(1159.8~2270.4) | 0.48(0.42~0.53) |
| **Kenya** | 11742.7(10284.9~13346.5) | 12646.5(10998.7~14415.1) | 0.30(0.23~0.39) | 1661(1194.2~2216.4) | 1816.8(1299.7~2432) | 0.33(0.24~0.41) |
| **Kiribati** | 12732.5(10675.4~15060.1) | 13703.3(11229.4~16582.7) | 0.21(0.15~0.24) | 1583.1(1115~2158.9) | 1714.1(1183.1~2386.4) | 0.23(0.17~0.26) |
| **Kuwait** | 16007.7(13473.6~18890.4) | 16643.8(13629~20277.3) | 0.21(0.15~0.30) | 2272.5(1563.9~3112.5) | 2393.8(1630.4~3348.9) | 0.29(0.20~0.41) |
| **Kyrgyzstan** | 11264.2(9665.7~12980) | 12619.9(10718.9~14785.5) | 0.44(0.37~0.53) | 1438.3(1020~1949.5) | 1669.6(1147.3~2291.7) | 0.57(0.46~0.70) |
| **Lao People's Democratic Republic** | 13230.3(11095.5~15547.9) | 13559.5(11015.2~16530.2) | 0.06(0.02~0.09) | 1614.1(1132.9~2192.2) | 1795.2(1239.2~2459.2) | 0.32(0.28~0.35) |
| **Latvia** | 12806.1(10896.1~14998.2) | 15655.5(12730.3~19310.9) | 0.56(0.46~0.61) | 1701.7(1203.7~2313.7) | 2171(1494.4~3025.8) | 0.72(0.61~0.78) |
| **Lebanon** | 18406.7(15518.8~21714.8) | 23351.7(18679.1~28767.9) | 0.75(0.70~0.80) | 2518.9(1772.6~3462.7) | 3453.1(2325.7~4894.8) | 1.00(0.97~1.04) |
| **Lesotho** | 12985.3(10883.1~15232) | 16172.2(13028.5~19663.9) | 0.66(0.57~0.73) | 1891.1(1332.5~2585.3) | 2489.3(1689.3~3447.9) | 0.84(0.73~0.93) |
| **Liberia** | 11959.3(10045.7~14111.3) | 12608.8(10474.3~15101.3) | 0.20(0.13~0.26) | 1636(1147.2~2204.4) | 1733.8(1196.8~2381.5) | 0.22(0.16~0.31) |
| **Libya** | 16750.1(14060.2~19848.8) | 19257.9(15737.6~23659.4) | 0.51(0.44~0.61) | 2345.5(1633~3185.9) | 2685.7(1809.4~3773.1) | 0.48(0.35~0.57) |
| **Lithuania** | 13409.5(11330~15655.3) | 16821.5(13828.7~20652.5) | 0.73(0.72~0.74) | 1796.7(1276.6~2422.8) | 2391.3(1629.1~3319.6) | 0.93(0.91~0.94) |
| **Luxembourg** | 17954.6(15227.3~21067.2) | 19584.4(16202.5~23551.7) | 0.33(0.22~0.41) | 2525.9(1789.7~3439.7) | 2792.7(1919~3867.2) | 0.39(0.27~0.49) |
| **Madagascar** | 12235.1(10355.3~14282.1) | 13504.1(11152.1~16198.4) | 0.34(0.31~0.39) | 1709.1(1199.7~2305.5) | 1930.5(1312.2~2658.6) | 0.44(0.39~0.50) |
| **Malawi** | 11964.5(10113.8~14154.6) | 13349.9(10960~16094.8) | 0.33(0.30~0.35) | 1620.4(1143.9~2188.6) | 1866.9(1295.2~2578.6) | 0.44(0.41~0.46) |
| **Malaysia** | 11368.6(9532.5~13503.1) | 13017.3(10522.8~16228.7) | 0.24(0.15~0.31) | 1593.5(1109.9~2151.6) | 1915.7(1288.6~2707.2) | 0.54(0.42~0.64) |
| **Maldives** | 12175.7(10243.7~14262.1) | 12431.6(10236.9~15116.5) | 0.07(0.05~0.09) | 1628.3(1145.8~2232.6) | 1707.1(1174.6~2335.4) | 0.17(0.14~0.21) |
| **Mali** | 11064(9317.9~13030.2) | 11484.6(9442~13799.2) | 0.16(0.11~0.24) | 1476.5(1051.5~1993) | 1571.6(1095.1~2167.3) | 0.24(0.17~0.31) |
| **Malta** | 18017(15190.7~21386.3) | 19800.4(16356.2~24269.8) | 0.40(0.33~0.50) | 2449.2(1718.4~3329.6) | 2783(1888.4~3881.7) | 0.48(0.39~0.56) |
| **Marshall Islands** | 12522.7(10495.7~14716.6) | 13429.4(11022.2~16186.7) | 0.20(0.14~0.23) | 1555.1(1088.4~2121.6) | 1698.1(1167.6~2358.6) | 0.24(0.17~0.27) |
| **Mauritania** | 10899.5(9181.4~12785.8) | 11513(9519.2~13887.1) | 0.21(0.17~0.27) | 1502.4(1060.3~2005.6) | 1617(1118.7~2217.3) | 0.28(0.23~0.35) |
| **Mauritius** | 12796(10779.6~14929) | 14079.6(11649.4~17037.5) | 0.32(0.30~0.35) | 1793.8(1255.8~2441.7) | 2076.7(1400.2~2899.6) | 0.50(0.47~0.54) |
| **Mexico** | 11705.2(10280.2~13281.3) | 14910.5(12984.3~16972.2) | 0.93(0.78~1.07) | 1560.6(1129.7~2083.7) | 2113.5(1497.1~2836.4) | 1.13(0.93~1.31) |
| **Micronesia (Federated States of)** | 12590.1(10605.6~14825.4) | 13442.4(11067.4~16216.4) | 0.19(0.13~0.22) | 1568.2(1102.7~2163.5) | 1699(1174.2~2348.1) | 0.24(0.18~0.27) |
| **Monaco** | 18329.1(15371.8~21620.3) | 21310.1(17228.9~26092.7) | 0.53(0.50~0.58) | 2647(1840.5~3591.7) | 3200.4(2132.7~4448.1) | 0.67(0.63~0.74) |
| **Mongolia** | 11918.4(10252.5~13764.2) | 11846.9(9951.1~13915.6) | -0.05(-0.1~-0.02) | 1547(1091~2112.1) | 1602.1(1109.8~2225.8) | 0.08(0.02~0.13) |
| **Montenegro** | 11832.9(10046.8~13805.8) | 14219.2(11772.9~17264.4) | 0.62(0.59~0.67) | 1524.3(1097.3~2047) | 1914.3(1314.9~2636.9) | 0.78(0.74~0.84) |
| **Morocco** | 17718.3(14993.9~20745) | 19822.9(16115.8~24050.6) | 0.49(0.34~0.69) | 2390.6(1667.4~3254.3) | 2820.9(1945.2~3968.2) | 0.63(0.43~0.80) |
| **Mozambique** | 12697(10655.7~14877.9) | 13924(11589.1~16773.9) | 0.28(0.25~0.30) | 1708.9(1200.3~2302.3) | 1983.8(1364.8~2723.4) | 0.47(0.44~0.49) |
| **Myanmar** | 12205.6(10341.9~14337) | 12780.8(10505.6~15423.1) | 0.13(0.10~0.16) | 1360.5(956.3~1830.6) | 1635.7(1116.5~2253) | 0.57(0.54~0.59) |
| **Namibia** | 11345.8(9592.7~13408.1) | 14266.1(11661.5~17324.7) | 0.67(0.56~0.73) | 1595.5(1136.1~2146.8) | 2096.3(1434.7~2885.5) | 0.80(0.69~0.86) |
| **Nauru** | 12134.5(10143.2~14371.8) | 13303.9(10889.7~16231.7) | 0.28(0.22~0.31) | 1566.1(1084~2146.5) | 1741.2(1169.3~2466.6) | 0.33(0.26~0.36) |
| **Nepal** | 12396.8(10546~14501) | 13460.1(11185.8~16157.1) | 0.35(0.28~0.44) | 1453.9(1034~1969.2) | 1768.9(1206~2503.3) | 0.75(0.65~0.89) |
| **Netherlands** | 17942.9(15353.5~20878.4) | 21791.5(17744.3~26483.8) | 0.71(0.62~0.85) | 2465.4(1739~3312.6) | 3097.4(2143~4295.5) | 0.84(0.73~1.02) |
| **New Zealand** | 22003.4(19242~24986.2) | 23035.3(19955~26501.6) | 0.15(0.13~0.16) | 3076(2190.7~4085.9) | 3269.8(2306.8~4362.5) | 0.19(0.18~0.21) |
| **Nicaragua** | 14446.8(12268.6~16919.3) | 16246.7(13513.2~19601.2) | 0.44(0.35~0.57) | 1809.6(1292.1~2459.7) | 2131.2(1473.6~2942.3) | 0.59(0.45~0.76) |
| **Niger** | 11408.4(9539.2~13360.5) | 11686.8(9769.2~13994.4) | 0.11(0.06~0.16) | 1558.7(1091.7~2130.5) | 1596.1(1107.6~2166.2) | 0.12(0.08~0.18) |
| **Nigeria** | 11041.8(9601.2~12553.7) | 11227.9(9768.1~12762.1) | 0.12(0.04~0.20) | 1525.1(1093.5~2022.4) | 1558.4(1116.8~2063.6) | 0.17(0.07~0.26) |
| **Niue** | 12260.4(10262.2~14505.9) | 13291.6(10893.6~16212.8) | 0.24(0.20~0.27) | 1559.3(1082.3~2134.5) | 1742.1(1179.2~2419.8) | 0.33(0.26~0.36) |
| **North Macedonia** | 11734.5(9955.5~13677.6) | 14433.3(11765~17482) | 0.72(0.66~0.81) | 1480.4(1054.8~1971.8) | 1930.5(1325.4~2646.9) | 0.95(0.87~1.06) |
| **Northern Mariana Islands** | 11846.5(9905.3~13976.9) | 13822.1(11296.6~17012.6) | 0.47(0.43~0.50) | 1543.9(1081.4~2108.5) | 1854.8(1258.8~2584.1) | 0.58(0.53~0.60) |
| **Norway** | 18726.9(16178.1~21482.3) | 20043.8(17250.5~23099) | 0.25(0.21~0.31) | 2665.3(1879~3592) | 2930.5(2056.3~3958.7) | 0.32(0.27~0.39) |
| **Oman** | 16282.8(13766~19127.4) | 18379.9(14869.1~22463.1) | 0.47(0.39~0.59) | 2227.4(1543.7~3036.3) | 2645.1(1765~3729.4) | 0.65(0.55~0.79) |
| **Pakistan** | 11537.6(9948~13227) | 12757.7(10919.1~14828.7) | 0.38(0.31~0.44) | 1389.7(990~1848.6) | 1533.2(1092.1~2058.9) | 0.43(0.36~0.52) |
| **Palau** | 12191.8(10186.8~14421.6) | 13213.6(10752.4~15901.6) | 0.24(0.18~0.26) | 1565.3(1086~2150) | 1739.8(1179.5~2447.6) | 0.31(0.24~0.34) |
| **Palestine** | 19670.8(16691.9~23165.8) | 21847(17700.4~26579.2) | 0.41(0.35~0.49) | 2727.6(1890~3740.8) | 3216.3(2146~4560) | 0.64(0.54~0.75) |
| **Panama** | 13548(11536~15817.5) | 15365.5(12693~18586.5) | 0.53(0.40~0.70) | 1682.9(1190.7~2269) | 2010.5(1377.4~2725.1) | 0.67(0.49~0.82) |
| **Papua New Guinea** | 12673.6(10594.1~14964.3) | 13159.6(10787.6~15868.6) | 0.10(0.04~0.12) | 1593.1(1116~2183.4) | 1677.1(1141.8~2342.5) | 0.13(0.06~0.16) |
| **Paraguay** | 16908.9(14246.6~19985.7) | 19869.1(16218.1~24046) | 0.53(0.52~0.55) | 2078.2(1460.1~2824.9) | 2605.2(1795.9~3667.2) | 0.74(0.73~0.77) |
| **Peru** | 15390.1(12891.1~18254.6) | 18455.3(15082~22547.6) | 0.73(0.56~0.96) | 1767.4(1252.1~2418.4) | 2258.1(1537.9~3151.8) | 0.98(0.76~1.28) |
| **Philippines** | 12147.1(10556.4~13864.1) | 13284.6(11509.2~15223.1) | 0.33(0.26~0.40) | 1533.1(1093~2053) | 1770.9(1254.2~2383) | 0.51(0.43~0.62) |
| **Poland** | 11327.5(9878~12889.9) | 12806.6(11087.1~14651.1) | 0.41(0.37~0.44) | 1383.8(995.7~1828.3) | 1667.7(1193~2225.9) | 0.62(0.57~0.67) |
| **Portugal** | 21338.4(17948.4~25254.6) | 24836.2(19975.8~30611) | 0.64(0.51~0.78) | 3066.2(2145.5~4166.3) | 3645.6(2446~5070.1) | 0.72(0.60~0.89) |
| **Puerto Rico** | 16241.1(13944.3~18510.8) | 17662.5(14670.6~21276.8) | 0.32(0.26~0.39) | 1765.3(1264.7~2372) | 1997.5(1369.9~2730.7) | 0.44(0.33~0.55) |
| **Qatar** | 16024.3(13481.4~18821.2) | 17007.4(14065.3~20595.7) | 0.21(0.14~0.27) | 2289.6(1597.2~3122.3) | 2487.9(1703.1~3460.7) | 0.29(0.21~0.37) |
| **Republic of Korea** | 13345.9(11440.2~15385.2) | 14087.3(11861.9~16686) | 0.18(0.14~0.21) | 1738.1(1265.6~2295.4) | 1951.1(1384.8~2650.2) | 0.40(0.36~0.43) |
| **Republic of Moldova** | 13026.1(11071.3~15261.4) | 14939.3(12220.4~18133.9) | 0.48(0.44~0.54) | 1666.4(1185.9~2253.5) | 1970.6(1370.1~2693.8) | 0.58(0.53~0.65) |
| **Romania** | 11567.5(9877.2~13462.1) | 13659.5(11352.1~16307.1) | 0.58(0.53~0.65) | 1468.5(1041.5~1973.1) | 1842.7(1279.1~2510) | 0.81(0.74~0.90) |
| **Russian Federation** | 12009.2(10447.2~13636.5) | 14202.4(12343.6~16179.1) | 0.55(0.54~0.56) | 1547.2(1107.5~2048.2) | 1924(1363.9~2574) | 0.72(0.69~0.74) |
| **Rwanda** | 12600.2(10665.8~14685.6) | 13646.6(11321.9~16468.6) | 0.25(0.24~0.26) | 1784.5(1247.6~2439.4) | 1985.3(1357~2761.3) | 0.33(0.32~0.35) |
| **Saint Kitts and Nevis** | 16703.3(14123.5~19765.6) | 18230.3(15027~21830.2) | 0.32(0.29~0.36) | 1839.2(1294.9~2496.1) | 2129.1(1446~2937.1) | 0.52(0.47~0.59) |
| **Saint Lucia** | 16576.5(14020.3~19554.2) | 18989.8(15945.4~22530.4) | 0.42(0.39~0.45) | 1799.5(1280.1~2456.5) | 2225.7(1521.2~3087.3) | 0.68(0.64~0.69) |
| **Saint Vincent and the Grenadines** | 16604.4(14027.2~19569.7) | 18573.1(15610.6~21944.3) | 0.36(0.36~0.37) | 1782.3(1258.8~2428) | 2152.7(1491.7~2946.9) | 0.62(0.61~0.63) |
| **Samoa** | 12485.1(10490.8~14754.7) | 13295(10838.2~16231.5) | 0.16(0.11~0.19) | 1560.4(1093.5~2133.2) | 1696.6(1170~2355.9) | 0.24(0.18~0.27) |
| **San Marino** | 18163.8(15249~21491.5) | 21170.9(17158.9~26000) | 0.62(0.49~0.79) | 2579.9(1771.9~3535) | 3111.2(2076.3~4404.6) | 0.75(0.59~0.97) |
| **Sao Tome and Principe** | 11311.3(9571.5~13309.1) | 11834.9(9805.7~14145.1) | 0.18(0.13~0.23) | 1566.9(1093.9~2121.2) | 1671.9(1148.2~2315.8) | 0.25(0.19~0.32) |
| **Saudi Arabia** | 15647.1(13164.8~18514.8) | 16884(13728.3~20681) | 0.36(0.25~0.50) | 2220.1(1533.6~3033.5) | 2469.1(1655.1~3472.9) | 0.49(0.36~0.67) |
| **Senegal** | 11072.8(9342.3~13042.1) | 12402(10245.9~14892.5) | 0.40(0.35~0.47) | 1522.3(1069.3~2064.5) | 1750.4(1182.3~2431.3) | 0.51(0.44~0.59) |
| **Serbia** | 11734.5(9991.9~13716.9) | 13248.7(10946.4~15877.3) | 0.42(0.39~0.45) | 1510(1077.4~2036.8) | 1748.4(1202.2~2400) | 0.50(0.46~0.55) |
| **Seychelles** | 11108.8(9461.6~13030.9) | 12796.5(10479.4~15523.1) | 0.44(0.39~0.46) | 1456.1(1034.6~1974.8) | 1766.9(1184.8~2472.6) | 0.60(0.56~0.62) |
| **Sierra Leone** | 11781.7(9915.7~13916.3) | 12104.9(9981.9~14583) | 0.16(0.06~0.26) | 1618.2(1138.5~2191.1) | 1672.3(1144.6~2314.7) | 0.20(0.11~0.33) |
| **Singapore** | 14072.3(12202.7~16101.7) | 14096.4(12030.3~16531.5) | 0.03(0.00~0.06) | 1985.2(1441~2636.1) | 2000.6(1406.4~2711.4) | 0.06(0.03~0.10) |
| **Slovakia** | 11635.2(9876.5~13501.5) | 13748.9(11397.4~16565.8) | 0.51(0.46~0.54) | 1491.6(1065.5~2006.4) | 1849.6(1264.6~2564.5) | 0.65(0.58~0.69) |
| **Slovenia** | 12217.7(10412.1~14201.4) | 13303.3(11048.2~15923.3) | 0.32(0.25~0.42) | 1641.8(1175.3~2200) | 1805.3(1253.7~2501) | 0.33(0.23~0.45) |
| **Solomon Islands** | 12834.1(10818.2~15151.9) | 13784.9(11359~16689.4) | 0.20(0.15~0.23) | 1577.7(1114.6~2163) | 1723.5(1177.5~2406.3) | 0.26(0.19~0.28) |
| **Somalia** | 12924.6(10899.2~15143.5) | 15068.3(12549.9~17979.9) | 0.49(0.47~0.51) | 1689.9(1188.4~2281.7) | 2018.7(1388.6~2746.3) | 0.56(0.53~0.57) |
| **South Africa** | 11811(10288~13486.5) | 14300.8(12340.9~16491.9) | 0.71(0.63~0.82) | 1693.1(1213~2265) | 2124.4(1508.9~2864.4) | 0.88(0.78~1.01) |
| **South Sudan** | 12290.6(10286.8~14406.3) | 13201.2(10915.2~15953.3) | 0.29(0.24~0.35) | 1746.8(1227~2369.9) | 1875.9(1283.2~2592.5) | 0.30(0.23~0.38) |
| **Spain** | 20705.3(18018~23400.7) | 24161.6(20327.3~28633.6) | 0.61(0.50~0.77) | 2581(1850.5~3426.4) | 3225.5(2264.7~4416.6) | 0.86(0.71~1.05) |
| **Sri Lanka** | 12251.7(10387.5~14362.2) | 13283.5(10933.4~15979.3) | 0.24(0.21~0.26) | 1631.7(1143.7~2230.5) | 1857.9(1289.4~2593.3) | 0.40(0.36~0.42) |
| **Sudan** | 18500.8(15738.1~21567.7) | 19566.5(16257.3~24015) | 0.24(0.14~0.37) | 2363.8(1653~3195.1) | 2649.8(1848.4~3699.3) | 0.46(0.34~0.62) |
| **Suriname** | 17434.2(14718.2~20488.3) | 20784(17284~24913.6) | 0.56(0.55~0.57) | 1997.7(1416.5~2718.7) | 2643.1(1802.4~3661.6) | 0.90(0.89~0.90) |
| **Sweden** | 17384(15308.9~19719.3) | 19659.8(16797~22744.1) | 0.47(0.38~0.60) | 2475.9(1763~3314.3) | 2897.4(2032.1~3947.3) | 0.58(0.46~0.73) |
| **Switzerland** | 20387.7(17231.7~23940.2) | 21521(17488.3~26394.4) | 0.22(0.17~0.29) | 2828.3(1998.7~3852.3) | 2999.7(2057.6~4160.5) | 0.25(0.19~0.33) |
| **Syrian Arab Republic** | 17660.9(14906.5~20837.3) | 19835.3(16221.8~24232.1) | 0.41(0.37~0.46) | 2321.3(1633.6~3168.9) | 2704.7(1842.5~3786.9) | 0.54(0.50~0.61) |
| **Taiwan (Province of China)** | 12110.6(10139.6~14225.2) | 11650.9(9501.5~14335.2) | -0.12(-0.13~-0.11) | 1524.5(1066.2~2050.4) | 1503.2(1040.3~2088.1) | -0.04(-0.05~-0.03) |
| **Tajikistan** | 10918.4(9319~12690.3) | 12332.1(10372.4~14663.2) | 0.41(0.39~0.44) | 1359.5(961.9~1823.3) | 1569.8(1085.3~2183.1) | 0.49(0.47~0.53) |
| **Thailand** | 13955.6(11925.5~16172.2) | 13718.5(11491.5~16529.8) | -0.07(-0.1~-0.05) | 1508.8(1069.9~2029.2) | 1609.6(1112.4~2188.8) | 0.18(0.14~0.21) |
| **Timor-Leste** | 12068.2(10169~14143.3) | 12533.8(10303.6~15005.9) | 0.11(0.08~0.13) | 1508.3(1065.7~2044.3) | 1658.4(1134.2~2263) | 0.29(0.26~0.31) |
| **Togo** | 11781.9(9874.8~13819.6) | 12402.2(10310.6~14824.1) | 0.21(0.17~0.26) | 1619.7(1131~2204.3) | 1724.6(1184.6~2336.9) | 0.24(0.18~0.28) |
| **Tokelau** | 12474(10470.3~14765.1) | 13370.2(10960.4~16095.2) | 0.20(0.14~0.23) | 1566.1(1084.9~2160.2) | 1742(1176.4~2420.7) | 0.32(0.24~0.35) |
| **Tonga** | 12324.4(10379.3~14511.7) | 13250.1(10892.2~16152) | 0.22(0.17~0.25) | 1518.5(1064.1~2061.8) | 1676.2(1156.4~2347.2) | 0.30(0.24~0.33) |
| **Trinidad and Tobago** | 16996.5(14433.5~20060.2) | 19771.9(16371.2~23602.8) | 0.46(0.39~0.50) | 1924.8(1370.7~2626.2) | 2444.7(1692.3~3381.5) | 0.73(0.64~0.78) |
| **Tunisia** | 17954.4(15209.2~21078.4) | 22051.3(17904.1~26918.5) | 0.67(0.66~0.67) | 2468(1710.6~3369.1) | 3281.9(2167.5~4624.6) | 0.93(0.92~0.95) |
| **Turkey** | 16295.6(14510.6~18169.7) | 18913.7(15630.4~22983.2) | 0.51(0.48~0.56) | 2156.5(1549.5~2829.4) | 2695.9(1812.3~3755.7) | 0.80(0.75~0.88) |
| **Turkmenistan** | 11129.2(9551.4~12790.3) | 11996.4(10077.1~14257.6) | 0.28(0.24~0.33) | 1415.5(1013.2~1894.7) | 1608.5(1104.6~2212.9) | 0.45(0.39~0.54) |
| **Tuvalu** | 12802.9(10749.5~15009) | 13644.7(11226.1~16581.2) | 0.18(0.12~0.21) | 1565.5(1095.4~2157.3) | 1735.4(1175.8~2401.8) | 0.32(0.25~0.35) |
| **Uganda** | 13439(11327.1~15789) | 14803(12188.2~17967) | 0.32(0.30~0.35) | 1953.1(1362.1~2659.9) | 2255.2(1520.1~3186.7) | 0.49(0.45~0.53) |
| **Ukraine** | 12268.2(10665.6~13942.6) | 14303.7(12194.9~16715.3) | 0.49(0.48~0.50) | 1605.5(1134.4~2143.4) | 1936(1358.3~2674.8) | 0.60(0.59~0.61) |
| **United Arab Emirates** | 14434(12204.3~17007.5) | 16699(13440.1~20711.5) | 0.54(0.48~0.63) | 2185(1530.3~2980.4) | 2533.7(1700.6~3500.6) | 0.56(0.49~0.67) |
| **United Kingdom** | 17399.3(15169.5~19845.5) | 19551.8(16929.3~22312.3) | 0.47(0.37~0.61) | 2495.6(1763.9~3364.1) | 2818.7(1987.1~3791.3) | 0.52(0.38~0.71) |
| **United Republic of Tanzania** | 12163.4(10313.4~14258.9) | 13101.5(10743.1~15818.5) | 0.26(0.24~0.29) | 1684.1(1194.7~2286.7) | 1884.7(1306.5~2593.9) | 0.40(0.38~0.43) |
| **United States of America** | 17283.5(15179.9~19492.5) | 22093.4(19456.9~24942.7) | 0.88(0.76~0.98) | 2332.2(1680~3075.7) | 3239(2320~4282.9) | 1.19(1.01~1.34) |
| **United States Virgin Islands** | 16560.4(14014.4~19540.2) | 18454.5(15416.9~22052.9) | 0.37(0.34~0.40) | 1828.8(1301.7~2500.3) | 2170.5(1494.2~2997.3) | 0.58(0.55~0.63) |
| **Uruguay** | 15987.5(13621.8~18685.3) | 19144.7(15871.1~23058.6) | 0.55(0.48~0.60) | 2170.1(1559.4~2921.6) | 2755(1891.1~3818) | 0.73(0.65~0.80) |
| **Uzbekistan** | 11175.3(9570.8~12860) | 11790.6(9987.2~13817.6) | 0.22(0.17~0.29) | 1394.1(1003~1870.7) | 1548.3(1091.7~2126.2) | 0.41(0.34~0.51) |
| **Vanuatu** | 12705.6(10727.6~14925.4) | 13724.9(11244.4~16710.4) | 0.22(0.17~0.25) | 1578.9(1110~2145.8) | 1739.1(1193.6~2402) | 0.28(0.21~0.31) |
| **Venezuela (Bolivarian Republic of)** | 15458.2(13025.8~18203.4) | 16500.6(13614.6~19711.1) | 0.21(0.19~0.25) | 1754.8(1253.6~2371.4) | 1938.6(1339.9~2644.3) | 0.36(0.32~0.43) |
| **Viet Nam** | 10306.5(8785.7~11901.4) | 10407.5(8790.8~12327.5) | -0.01(-0.09~0.04) | 1249.3(891~1683.8) | 1375.6(956.9~1881.7) | 0.26(0.17~0.34) |
| **Yemen** | 17801.6(14936.4~21063.6) | 18271.2(15054.1~22150.3) | 0.09(0.05~0.13) | 2392(1663.6~3290.8) | 2454.5(1693.4~3391) | 0.12(0.07~0.19) |
| **Zambia** | 11741.4(9938.4~13863.9) | 13336.7(10960.1~16271.2) | 0.41(0.37~0.43) | 1615(1145~2184) | 1900.6(1317.3~2628.7) | 0.51(0.46~0.54) |
| **Zimbabwe** | 10796.8(9107.6~12690) | 12352(10156.4~15048.6) | 0.42(0.40~0.43) | 1470.3(1043~1978.5) | 1706(1178.3~2338.9) | 0.47(0.45~0.48) |

DALYs, disability-adjusted life years; UI, uncertainty interval; AAPC, average annual percent change; CI, confidence interval; SDI, socio-demographic index.

**Table S2. Global and regional age-standardized DALYs per 100,000 population (95% UI) of ten mental disorders in adolescents and young adults, 2021**

|  | Schizophrenia | Depressive disorders | Bipolar disorder | Anxiety disorders | Eating disorders | Autism spectrum disorders | Attention-deficit/hyperactivity disorder | Conduct disorder | Idiopathic developmental intellectual disability | Other mental disorders |
| --- | --- | --- | --- | --- | --- | --- | --- | --- | --- | --- |
| **Total** | 69.7(43.5~107.9) | 564.5(351.2~853) | 91.3(55.9~141.4) | 609.4(377.5~909.1) | 76(40.9~129.2) | 158.5(107~223.1) | 26.6(13.9~44.8) | 219.4(114.7~356.4) | 67.3(31.5~113.8) | 36.4(20.8~57.8) |
| Male | 74.8(46.5~116.4) | 437.6(270.4~663.6) | 88.6(54.2~137.5) | 467.6(287.2~702.2) | 54.1(27.2~97.9) | 210.8(143~297.3) | 37.7(19.6~63.1) | 276(145.7~444.2) | 66.2(28.8~114.6) | 44.7(26.2~70.7) |
| Female | 64.4(39.8~100) | 697.3(435.1~1050) | 94.1(57.1~146.2) | 758.3(470.1~1124.3) | 98.8(54.6~163.9) | 103.5(70.8~145.9) | 15(7.9~25.5) | 159.5(81.3~268.3) | 68.5(34.4~113.2) | 27.8(15.2~44.3) |
| **SDI** |  |  |  |  |  |  |  |  |  |  |
| Low | 59.2(35.3~96.3) | 584.4(354.4~897.1) | 87.6(50.4~141.2) | 520.6(315.2~793.6) | 47.7(25.3~82.7) | 166.6(114.2~234) | 14.2(7.2~24.0) | 223.7(117.4~363.2) | 81.4(37.9~137.4) | 36.2(20.5~57.8) |
| Low-Middle | 67.6(40.4~107.3) | 553.3(342.2~838.4) | 80.8(48.4~128.3) | 522.0(320.4~778.6) | 61.6(32.3~106.4) | 147.4(100.1~207.4) | 18.3(9.4~30.7) | 216.8(112.9~353.6) | 106.4(53.8~175.2) | 34.4(19.1~54.8) |
| Middle | 74.2(45.8~115.5) | 460.9(285.7~695.8) | 87.6(53.3~136) | 644.7(401.6~953.1) | 71.1(37.5~121.8) | 144.3(97.7~203.7) | 33.2(17.3~55.7) | 215.2(112.6~350.5) | 53.2(24~91.4) | 33.4(18.6~53.2) |
| High-Middle | 76.9(48.1~114.7) | 511.4(311.6~775.7) | 82.2(48.9~131.4) | 689.4(425~1034.2) | 90(48.2~151.8) | 163(110.7~228.6) | 40.3(21.5~67.3) | 216.6(114~352.2) | 23.8(7.6~46.0) | 36.5(20.8~57.9) |
| High | 72.8(45.6~110.5) | 936.2(602.9~1396.4) | 151.1(96.8~225.9) | 845.0(523.3~1256.5) | 167.4(94.4~277.1) | 211.9(145.9~295.7) | 41.1(21.0~69.3) | 234.9(123.5~380.7) | 17.6(4.6~34.3) | 50.8(30.7~79) |
| **Regions** |  |  |  |  |  |  |  |  |  |  |
| Andean Latin America | 61.7(31.6~106.1) | 492.0(288.9~774) | 182.7(100.7~305.3) | 1011.1(584.7~1600.9) | 113.9(59.2~193.2) | 139.9(95~197.1) | 52.9(27.3~88.5) | 226.6(117.9~377.7) | 19.5(5.5~38.9) | 40.9(22.4~65.9) |
| Australasia | 127.7(87.1~172.4) | 1038.6(634.4~1612) | 286(172.2~448.5) | 914.1(532.9~1430.1) | 351.1(208.2~564) | 236.3(161.5~331.7) | 78.7(42.3~125.4) | 241.9(126.4~396.8) | 14.3(3.2~30.1) | 62.8(38.0~94.0) |
| Caribbean | 59.3(32.1~100.5) | 572.9(339.2~896) | 181.6(100.7~301.7) | 724.1(421.7~1146.2) | 85.0(45.3~144.9) | 137.4(94.5~192.6) | 73.9(39.2~123.8) | 222.5(116.2~365.1) | 22.4(5.8~44.6) | 40.4(23.0~63.9) |
| Central Asia | 52.3(27.6~91.9) | 520.1(315.2~806.3) | 84.2(45.1~143) | 375.6(217.3~585.1) | 62.7(33.7~107.4) | 180.9(123.4~255.7) | 24.8(12.6~41.9) | 231.5(119.9~381.1) | 29.3(9.1~56.5) | 40.8(23.1~65.2) |
| Central Europe | 52.2(29.6~86.3) | 418.7(251.6~640.9) | 91.5(53.0~148.2) | 622.2(373.7~938.3) | 80.4(43.2~135) | 194.4(134.3~270.7) | 25.2(12.9~42.4) | 238.0(125.5~387.0) | 17.3(2.9~36.4) | 37.7(21.7~59.8) |
| Central Latin America | 62.0(36.9~99.2) | 525.5(325.2~801.3) | 182.8(110.7~284.9) | 727.4(444.6~1103.7) | 96.7(50.5~166.2) | 155.1(105.2~219.7) | 35.2(18.6~59.1) | 233.5(122.2~380.8) | 15.2(2.5~32) | 35.7(20.3~56.5) |
| Central Sub-Saharan Africa | 52.3(26.5~91.4) | 879.4(506.6~1393.3) | 90.7(46.2~156.1) | 555.6(320.9~895.9) | 45.1(22.8~78.2) | 181.3(122.9~257) | 12.4(6.2~21.4) | 224.4(116.3~366.2) | 31.1(8.2~62.8) | 40.3(23.1~64.2) |
| East Asia | 90.7(56.7~133.6) | 220.8(138.1~328.7) | 32.9(19.7~52.3) | 549.5(341.4~817.8) | 55.8(28.7~96.3) | 134.3(91.4~189.9) | 54.1(28.9~89.8) | 188.0(97.6~307.7) | 16.4(4.4~32.7) | 32.2(17.6~51.6) |
| Eastern Europe | 49.9(29.8~78.2) | 554.5(342.7~845.6) | 80.7(48.7~126.2) | 668.4(419.7~991.4) | 78.8(43.0~133.7) | 189.7(130.0~266.5) | 26(13.5~44.4) | 249.8(132.7~402.6) | 20.2(3.7~41.8) | 32.1(17.7~51.4) |
| Eastern Sub-Saharan Africa | 53.3(30.7~87.9) | 654.7(392.6~1004.8) | 103(60.2~166.1) | 570.8(346~872.9) | 45.8(23.8~78.6) | 183.2(125.0~257.3) | 12.5(6.5~21.4) | 233.1(123.5~377.8) | 30.7(8.3~60.3) | 36.5(20.5~58.1) |
| High-income Asia Pacific | 61.3(35.9~98.1) | 516.6(324.4~772.8) | 94.1(56.7~147.3) | 542.2(335.6~822.6) | 170.9(97.3~281) | 312(214.2~437.6) | 33.2(16.9~56.2) | 234.7(122.9~377.9) | 3.9(0.1~12.7) | 42.6(24.4~68) |
| High-income North America | 84.4(53.4~126.5) | 1356.7(887.0~2002.4) | 168.1(108.9~242.5) | 810(518.9~1189.3) | 176.5(97.3~298) | 219.5(151.3~307.4) | 51.9(26.1~88.3) | 222.8(116.2~366.6) | 24.8(6.8~45.9) | 62.1(37.2~96.5) |
| North Africa and Middle East | 64.5(36.6~106.8) | 871.4(505.1~1374) | 155.4(89.7~253) | 1001.1(604.5~1530.4) | 85.9(45.0~149.4) | 153.8(104.0~216.5) | 28.7(14.9~48.1) | 207.7(108.3~333.7) | 74.5(33.4~128.9) | 39.6(22.9~63.4) |
| Oceania | 75.7(40.8~131.6) | 459.1(260.4~736.2) | 43.4(21.2~77.4) | 623.8(339.8~1021.6) | 41.1(21.4~71.3) | 137.3(93.4~191.6) | 27.0(13.5~46.5) | 208.5(105.8~346) | 35.9(13.1~65.2) | 40.8(22.6~67.4) |
| South Asia | 70.4(42.1~109.7) | 496.5(310.4~750.7) | 53.1(31.7~83.7) | 389.9(239.7~571.2) | 61.1(31.9~106.3) | 140(95.6~197.2) | 13.9(7.0~23.4) | 213.8(111.0~350.3) | 166.4(87.2~269) | 32.1(17.5~51.6) |
| Southeast Asia | 85.3(50.8~136.6) | 427.6(258.6~652.4) | 51.7(30.2~83.4) | 572.9(358.3~862.3) | 52.6(27.6~89.8) | 138.6(94.3~195.5) | 22.6(11.6~38.7) | 224(117.4~367.5) | 33.8(13.1~62.6) | 35.2(19.9~56.3) |
| Southern Latin America | 65.1(33.7~110.5) | 829.3(508.3~1257.9) | 155.2(87.2~255.5) | 983.2(548.7~1533.6) | 141.6(75.1~239.7) | 214.8(145.8~302.9) | 31.8(16.3~53.6) | 236.1(122.7~384.6) | 23.1(6.1~44.2) | 50.5(29.4~80.6) |
| Southern Sub-Saharan Africa | 54.3(32.4~86.9) | 666.3(413.2~997.1) | 88.3(51.8~139.1) | 675.2(420.5~992.2) | 69.3(36.5~118.5) | 185.3(127~260.6) | 12.7(6.4~22) | 238.9(124.7~390.5) | 16.7(3.3~35.5) | 34(18.9~53.9) |
| Tropical Latin America | 61.5(38.2~94.3) | 602.1(381~908.3) | 257.2(158.9~392.5) | 1111.8(697.2~1627.3) | 99.6(53~166.3) | 125.3(84.8~175.9) | 52.3(27.4~89.3) | 235.6(121.8~382.9) | 18.4(5~35.9) | 31.3(17.3~50.2) |
| Western Europe | 58(35.5~92.3) | 888.1(538.2~1365.6) | 177.4(107.2~278.5) | 1145.3(700.2~1697.8) | 188.8(106.8~311.9) | 180.6(123.9~252) | 34.6(17.7~58.1) | 263.9(140.2~426.3) | 20.7(6.5~39.4) | 46.8(27.4~73.2) |
| Western Sub-Saharan Africa | 57.9(34.2~93.4) | 464.2(282.6~712.2) | 82.6(48.2~132.6) | 490.5(297.9~743.6) | 55.0(28.7~95.1) | 180.8(124.3~253.3) | 13.1(6.7~22.2) | 228.0(119~372) | 21.6(4.8~44.5) | 35.6(20.0~57.0) |

DALYs, disability-adjusted life years; UI, uncertainty interval; SDI, socio-demographic index.

**Table S3. Global and regional average annual percent change (95% CI) in age-standardized prevalence per 100,000 population of ten mental disorders among adolescents and young adults**

|  | Schizophrenia | Depressive disorders | Bipolar disorder | Anxiety disorders | Eating disorders | Autism spectrum disorders | Attention-deficit/hyperactivity disorder | Conduct disorder | Idiopathic developmental intellectual disability | Other mental disorders |
| --- | --- | --- | --- | --- | --- | --- | --- | --- | --- | --- |
| Total | -0.12(-0.13~-0.12) | 0.76(0.64~0.93) | 0.16(0.15~0.16) | 0.68(0.58~0.8) | 0.53(0.53~0.54) | 0.10(0.10~0.10) | -0.44(-0.45~-0.43) | 0.10(0.09~0.10) | -0.38(-0.39~-0.37) | 0.02(0.02~0.02) |
| Male | -0.11(-0.11~-0.1) | 0.83(0.72~0.99) | 0.18(0.17~0.18) | 0.7(0.61~0.83) | 0.64(0.63~0.64) | 0.05(0.05~0.05) | -0.45(-0.46~-0.44) | 0.05(0.05~0.05) | -0.49(-0.51~-0.48) | 0.01(0.01~0.01) |
| Female | -0.14(-0.15~-0.13) | 0.73(0.6~0.9) | 0.14(0.14~0.14) | 0.75(0.66~0.88) | 0.49(0.49~0.5) | 0.16(0.16~0.16) | -0.45(-0.46~-0.45) | 0.15(0.15~0.16) | -0.26(-0.27~-0.25) | 0.02(0.02~0.02) |
| SDI |  |  |  |  |  |  |  |  |  |  |
| Low | -0.04(-0.05~-0.04) | 0.38(0.29~0.5) | 0.07(0.06~0.07) | 0.65(0.57~0.75) | 0.35(0.35~0.36) | 0.07(0.06~0.07) | -0.02(-0.03~-0.02) | 0.03(0.03~0.03) | -0.58(-0.59~-0.57) | 0.02(0.02~0.02) |
| Low-Middle | -0.04(-0.05~-0.04) | 0.56(0.42~0.75) | -0.01(-0.01~0.00) | 0.82(0.70~0.99) | 0.83(0.82~0.83) | 0.09(0.08~0.09) | -0.17(-0.18~-0.16) | 0.08(0.08~0.09) | -1.11(-1.12~-1.1) | 0(0~0) |
| Middle | -0.12(-0.13~-0.11) | 0.62(0.51~0.78) | 0.38(0.38~0.39) | 0.82(0.73~0.95) | 1.18(1.16~1.19) | 0.18(0.18~0.18) | -0.27(-0.28~-0.26) | 0.14(0.14~0.14) | -0.63(-0.64~-0.62) | 0.04(0.04~0.04) |
| High-Middle | 0.13(0.11~0.13) | 0.52(0.42~0.62) | 0.34(0.33~0.36) | 0.75(0.71~0.80) | 0.95(0.94~0.96) | 0.15(0.14~0.15) | 0.08(0.05~0.1) | 0.07(0.07~0.08) | -0.66(-0.68~-0.65) | 0.11(0.11~0.12) |
| High | -0.01(-0.02~-0.01) | 1.59(1.43~1.84) | 0.17(0.17~0.17) | 1.13(0.99~1.32) | 0.44(0.42~0.45) | -0.01(-0.01~0) | 0.29(0.28~0.29) | 0.04(0.04~0.05) | -0.61(-0.63~-0.6) | 0.09(0.09~0.09) |
| Regions |  |  |  |  |  |  |  |  |  |  |
| Andean Latin America | 0.04(0.03~0.04) | 1.29(0.93~1.81) | -0.01(-0.01~-0.01) | 1.67(1.32~2.15) | 0.6(0.59~0.61) | 0.08(0.08~0.09) | 0.05(0.05~0.05) | 0.03(0.03~0.03) | -1.13(-1.15~-1.11) | 0.03(0.03~0.03) |
| Australasia | 0.01(0.01~0.02) | 0.37(0.3~0.47) | -0.01(-0.01~-0.01) | 0.31(0.25~0.38) | 0.84(0.73~0.97) | 0.07(0.06~0.07) | 0.1(0.1~0.1) | -0.03(-0.03~-0.03) | 1.94(1.87~2.03) | 0(-0.01~0) |
| Caribbean | -0.04(-0.04~-0.04) | 0.38(0.27~0.53) | -0.02(-0.02~-0.02) | 0.89(0.79~1.02) | 0.14(0.12~0.15) | -0.04(-0.05~-0.04) | 0.03(0.03~0.03) | 0.02(0.02~0.02) | 0.24(0.22~0.26) | 0.02(0.01~0.02) |
| Central Asia | 0.02(0.01~0.02) | 0.71(0.6~0.84) | 0(0~0) | 0.89(0.8~1) | 0.39(0.36~0.41) | 0.01(0.01~0.01) | 0.03(0.03~0.03) | 0.01(0.01~0.01) | -0.62(-0.67~-0.58) | 0.02(0.02~0.02) |
| Central Europe | 0.06(0.05~0.06) | 0.72(0.62~0.82) | -0.04(-0.04~-0.04) | 1.25(1.18~1.36) | 0.86(0.85~0.87) | 0.06(0.06~0.06) | 0.02(0.02~0.02) | 0.01(0.01~0.01) | -1.56(-1.58~-1.55) | -0.02(-0.03~-0.02) |
| Central Latin America | 0.01(0~0.02) | 1.33(1.12~1.65) | -0.02(-0.02~-0.02) | 1.52(1.29~1.74) | 0.17(0.16~0.17) | 0.04(0.04~0.05) | 0(0~0.01) | 0(0~0) | -0.62(-0.64~-0.61) | 0.04(0.04~0.04) |
| Central Sub-Saharan Africa | -0.06(-0.06~-0.06) | 0.28(0.14~0.41) | 0(0~0) | 0.49(0.35~0.62) | -0.14(-0.15~-0.13) | 0.04(0.04~0.05) | 0(0~0) | 0(0~0) | 1.07(1.04~1.1) | 0(0~0) |
| East Asia | 0.15(0.14~0.16) | -1.06(-1.11~-1.03) | 0(0~0.01) | 0.01(-0.14~0.11) | 1.67(1.65~1.69) | 0.23(0.22~0.24) | 0.36(0.33~0.39) | 0.15(0.15~0.16) | -1.28(-1.3~-1.27) | 0.04(0.04~0.04) |
| Eastern Europe | 0.13(0.11~0.15) | 0.93(0.9~0.97) | -0.01(-0.01~-0.01) | 1.24(1.2~1.27) | 0.26(0.24~0.28) | 0.05(0.05~0.05) | 0.01(0.01~0.01) | 0.01(0.01~0.01) | -0.28(-0.34~-0.23) | -0.01(-0.01~-0.01) |
| Eastern Sub-Saharan Africa | 0.02(0.02~0.02) | 0.41(0.36~0.48) | 0(0~0) | 0.69(0.65~0.73) | 0.37(0.36~0.37) | 0.05(0.05~0.05) | 0.01(0.01~0.01) | 0(0~0) | -0.52(-0.54~-0.5) | 0.01(0.01~0.01) |
| High-income Asia Pacific | -0.01(-0.01~0) | 0.91(0.88~0.96) | -0.07(-0.08~-0.07) | 0.6(0.58~0.63) | 0.62(0.61~0.64) | 0.22(0.21~0.23) | -0.04(-0.05~-0.03) | -0.03(-0.03~-0.03) | -3.9(-3.95~-3.86) | 0(0~0) |
| High-income North America | -0.29(-0.31~-0.28) | 2.04(1.85~2.33) | -0.04(-0.04~-0.04) | 1.37(1.11~1.58) | 0.01(0~0.03) | 0.05(0.04~0.06) | 0.28(0.27~0.3) | 0.17(0.15~0.18) | -0.33(-0.35~-0.32) | 0(0~0) |
| North Africa and Middle East | -0.02(-0.02~-0.02) | 0.83(0.67~1.06) | -0.01(-0.01~-0.01) | 0.81(0.68~1) | 0.41(0.39~0.42) | 0.01(0.01~0.01) | -0.32(-0.34~-0.29) | -0.02(-0.02~-0.02) | -0.57(-0.58~-0.56) | 0.05(0.05~0.06) |
| Oceania | 0.02(0.02~0.03) | 0.09(0~0.13) | 0(0~0) | 0.4(0.28~0.45) | 0.16(0.15~0.16) | -0.02(-0.03~-0.02) | 0.01(0.01~0.01) | 0.01(0.01~0.01) | -0.21(-0.22~-0.2) | 0.01(0.01~0.01) |
| South Asia | -0.03(-0.03~-0.02) | 0.42(0.19~0.62) | 0(0~0) | 0.73(0.53~0.93) | 1.1(1.09~1.11) | 0(0~0) | -0.02(-0.03~-0.01) | 0.13(0.12~0.14) | -0.9(-0.91~-0.89) | -0.02(-0.02~-0.02) |
| Southeast Asia | 0.06(0.02~0.08) | 0.66(0.64~0.69) | -0.01(-0.01~-0.01) | 1.01(1~1.02) | 0.97(0.97~0.98) | 0.06(0.06~0.06) | -0.2(-0.2~-0.2) | 0.03(0.03~0.03) | -2.23(-2.27~-2.2) | -0.03(-0.03~-0.03) |
| Southern Latin America | 0.02(0.02~0.03) | 0.93(0.73~1.24) | -0.18(-0.19~-0.17) | 1.07(0.87~1.28) | 0.53(0.52~0.54) | 0.04(0.04~0.04) | 0(0~0) | 0.01(0.01~0.01) | -0.76(-0.78~-0.75) | 0.01(0.01~0.02) |
| Southern Sub-Saharan Africa | -0.01(-0.02~0) | 1.1(0.98~1.22) | 0(0~0) | 1.17(1.09~1.27) | 0.1(0.09~0.11) | 0.04(0.04~0.04) | 0.03(0.03~0.04) | 0.01(0.01~0.01) | 0.03(0.02~0.04) | 0.06(0.06~0.07) |
| Tropical Latin America | 0.01(-0.01~0.01) | 0.81(0.56~1.07) | -0.01(-0.01~-0.01) | 1.46(1.32~1.6) | 0.45(0.44~0.46) | 0.02(0.02~0.02) | 0.01(-0.03~0.05) | -0.04(-0.04~-0.03) | -0.93(-0.95~-0.92) | 0.03(0.03~0.03) |
| Western Europe | -0.07(-0.08~-0.07) | 0.7(0.45~0.87) | 0.05(0.05~0.06) | 0.95(0.78~1.15) | 0.39(0.39~0.4) | 0.02(0.02~0.02) | 0.11(0.1~0.11) | 0(0~0) | -0.97(-0.99~-0.95) | 0.02(0.02~0.02) |
| Western Sub-Saharan Africa | -0.01(-0.02~-0.01) | 0.2(0.11~0.31) | 0(0~0) | 0.39(0.3~0.49) | 0.28(0.27~0.29) | 0.05(0.04~0.05) | 0.08(0.07~0.08) | 0.03(0.03~0.03) | -0.31(-0.34~-0.29) | 0.01(0.01~0.01) |

CI, confidence interval; SDI, socio-demographic index.

**Table S4. Global and regional average annual percent change (95% CI) in age-standardized DALYs per 100,000 population of ten mental disorders among adolescents and young adults**

|  | Schizophrenia | Depressive disorders | Bipolar disorder | Anxiety disorders | Eating disorders | Autism spectrum disorders | Attention-deficit/hyperactivity disorder | Conduct disorder | Idiopathic developmental intellectual disability | Other mental disorders |
| --- | --- | --- | --- | --- | --- | --- | --- | --- | --- | --- |
| **Total** | 0.02(0.01~0.02) | 0.84(0.7~1.03) | 0.16(0.15~0.16) | 0.68(0.58~0.8) | 0.53(0.52~0.54) | 0.10(0.10~0.10) | -0.44(-0.45~-0.43) | 0.10(0.10~0.11) | -0.16(-0.17~-0.15) | 0.02(0.01~0.02) |
| Male | -0.11(-0.12~-0.11) | 0.93(0.8~1.12) | 0.18(0.17~0.18) | 0.7(0.61~0.83) | 0.64(0.63~0.64) | 0.06(0.06~0.06) | -0.45(-0.46~-0.44) | 0.06(0.06~0.06) | -0.28(-0.3~-0.26) | 0.01(0.01~0.01) |
| Female | -0.15(-0.16~-0.14) | 0.67(0.45~0.81) | 0.14(0.13~0.14) | 0.74(0.65~0.87) | 0.48(0.47~0.49) | 0.16(0.16~0.16) | -0.45(-0.46~-0.45) | 0.16(0.15~0.16) | -0.03(-0.04~-0.02) | 0.01(0.01~0.02) |
| **SDI** |  |  |  |  |  |  |  |  |  |  |
| Low | -0.02(-0.04~-0.01) | 0.46(0.36~0.61) | 0.1(0.09~0.1) | 0.68(0.59~0.78) | 0.38(0.37~0.39) | 0.09(0.09~0.09) | -0.01(-0.01~0) | 0.05(0.05~0.06) | -0.44(-0.46~-0.43) | 0.04(0.04~0.05) |
| Low-Middle | -0.03(-0.04~-0.03) | 0.64(0.48~0.86) | 0.01(0~0.01) | 0.84(0.71~1.00) | 0.84(0.83~0.85) | 0.10(0.10~0.10) | -0.16(-0.17~-0.15) | 0.10(0.09~0.11) | -0.89(-0.9~-0.88) | 0.02(0.01~0.02) |
| Middle | -0.12(-0.13~-0.12) | 0.68(0.55~0.87) | 0.39(0.38~0.39) | 0.82(0.73~0.95) | 1.18(1.17~1.19) | 0.19(0.18~0.19) | -0.27(-0.28~-0.25) | 0.15(0.14~0.15) | -0.30(-0.31~-0.29) | 0.04(0.03~0.04) |
| High-Middle | 0.13(0.12~0.13) | 0.6(0.49~0.7) | 0.34(0.33~0.35) | 0.75(0.71~0.80) | 0.95(0.93~0.96) | 0.15(0.15~0.15) | 0.08(0.06~0.10) | 0.08(0.07~0.08) | -0.50(-0.51~-0.49) | 0.11(0.11~0.12) |
| High | -0.04(-0.05~-0.03) | 1.77(1.6~2.04) | 0.16(0.15~0.16) | 1.10(0.97~1.29) | 0.42(0.41~0.44) | -0.01(-0.02~-0.01) | 0.29(0.28~0.29) | 0.03(0.03~0.04) | -0.54(-0.56~-0.53) | 0.08(0.07~0.08) |
| **Regions** |  |  |  |  |  |  |  |  |  |  |
| Andean Latin America | 0.04(0.03~0.05) | 1.47(1.07~2.07) | -0.01(-0.02~0) | 1.67(1.33~2.15) | 0.59(0.58~0.61) | 0.1(0.09~0.1) | 0.04(0.04~0.05) | 0.04(0.04~0.05) | -0.96(-0.98~-0.94) | 0.04(0.03~0.05) |
| Australasia | **0.01(0~0.02)** | **0.42(0.34~0.52)** | **0(-0.01~0.01)** | **0.31(0.25~0.38)** | **0.84(0.73~0.96)** | **0.07(0.07~0.07)** | **0.11(0.1~0.11)** | **-0.02(-0.02~-0.01)** | **2.32(2.24~2.42)** | **0.01(0~0.02)** |
| Caribbean | 0.03(0.03~0.04) | 0.41(0.29~0.58) | -0.02(-0.03~-0.02) | 0.88(0.78~1.02) | 0.14(0.13~0.15) | -0.04(-0.05~-0.04) | 0.03(0.03~0.03) | 0.01(0.01~0.02) | 0.3(0.28~0.31) | 0.03(0.03~0.04) |
| Central Asia | **0.02(0.01~0.04)** | **0.84(0.71~0.98)** | **0(0~0.02)** | **0.9(0.81~1.01)** | **0.38(0.35~0.41)** | **0.02(0.01~0.02)** | **0.03(0.03~0.04)** | **0.02(0.02~0.03)** | **-0.5(-0.53~-0.46)** | **0.02(0.01~0.04)** |
| Central Europe | -0.02(-0.02~-0.01) | 0.86(0.74~0.98) | -0.03(-0.04~-0.03) | 1.26(1.19~1.36) | 0.89(0.87~0.9) | 0.07(0.07~0.07) | 0.02(0.01~0.02) | 0.02(0.01~0.02) | -1.36(-1.38~-1.34) | -0.02(-0.02~-0.01) |
| Central Latin America | **0.04(0.03~0.05)** | **1.52(1.28~1.88)** | **-0.02(-0.02~-0.01)** | **1.52(1.3~1.74)** | **0.17(0.16~0.18)** | **0.05(0.05~0.06)** | **0.01(0~0.01)** | **0.01(0~0.01)** | **-0.47(-0.49~-0.45)** | **0.04(0.03~0.05)** |
| Central Sub-Saharan Africa | 0.03(0.02~0.04) | 0.34(0.2~0.49) | 0.03(0.02~0.04) | 0.53(0.39~0.65) | -0.1(-0.11~-0.09) | 0.07(0.07~0.08) | 0.02(0.01~0.04) | 0.03(0.03~0.04) | 0.88(0.83~0.9) | 0.03(0.02~0.04) |
| East Asia | **0.04(0.03~0.05)** | **-1.25(-1.3~-1.21)** | **0.01(0.01~0.02)** | **0.03(-0.13~0.12)** | **1.69(1.67~1.71)** | **0.24(0.23~0.25)** | **0.36(0.33~0.39)** | **0.17(0.16~0.17)** | **-1.02(-1.04~-1.01)** | **0.04(0.03~0.05)** |
| Eastern Europe | -0.01(-0.01~0) | 1.07(1.04~1.12) | 0(-0.01~0) | 1.24(1.21~1.28) | 0.27(0.25~0.29) | 0.05(0.04~0.05) | 0.01(0.01~0.02) | 0.01(0.01~0.02) | -0.19(-0.24~-0.15) | -0.01(-0.01~0) |
| Eastern Sub-Saharan Africa | **0.04(0.03~0.04)** | **0.52(0.45~0.6)** | **0.03(0.02~0.03)** | **0.68(0.63~0.73)** | **0.39(0.38~0.4)** | **0.07(0.07~0.07)** | **0.03(0.03~0.04)** | **0.02(0.02~0.02)** | **-0.56(-0.59~-0.54)** | **0.04(0.03~0.04)** |
| High-income Asia Pacific | 0(-0.01~0) | 1.04(1~1.1) | -0.08(-0.08~-0.07) | 0.6(0.57~0.62) | 0.63(0.61~0.65) | 0.22(0.21~0.23) | -0.04(-0.05~-0.02) | -0.03(-0.03~-0.02) | -3.84(-3.9~-3.8) | 0(-0.01~0) |
| High-income North America | **-0.03(-0.03~-0.02)** | **2.3(2.09~2.59)** | **-0.07(-0.08~-0.07)** | **1.34(1.08~1.54)** | **-0.03(-0.05~-0.01)** | **0.03(0.02~0.04)** | **0.28(0.27~0.29)** | **0.16(0.14~0.17)** | **-0.34(-0.36~-0.33)** | **-0.03(-0.03~-0.02)** |
| North Africa and Middle East | 0.06(0.06~0.07) | 0.91(0.74~1.17) | -0.01(-0.01~0) | 0.81(0.68~0.99) | 0.41(0.39~0.42) | 0.01(0.01~0.02) | -0.32(-0.34~-0.29) | -0.02(-0.02~-0.02) | -0.45(-0.46~-0.44) | 0.06(0.06~0.07) |
| Oceania | **0.02(0.01~0.03)** | **0.12(0.01~0.16)** | **0.01(0~0.03)** | **0.42(0.31~0.47)** | **0.18(0.16~0.19)** | **0(-0.01~0.01)** | **0.03(0.03~0.04)** | **0.03(0.02~0.04)** | **-0.15(-0.17~-0.14)** | **0.02(0.01~0.03)** |
| South Asia | -0.01(-0.02~0) | 0.54(0.38~0.78) | 0.03(0.03~0.03) | 0.75(0.56~0.94) | 1.12(1.11~1.13) | 0.01(0.01~0.02) | -0.01(-0.02~0) | 0.15(0.14~0.16) | -0.61(-0.63~-0.58) | -0.01(-0.02~0) |
| Southeast Asia | **-0.02(-0.02~-0.01)** | **0.79(0.76~0.82)** | **0.01(0~0.01)** | **1.02(1.01~1.04)** | **0.98(0.97~0.99)** | **0.07(0.07~0.08)** | **-0.19(-0.2~-0.19)** | **0.05(0.04~0.05)** | **-2.2(-2.23~-2.17)** | **-0.02(-0.02~-0.01)** |
| Southern Latin America | 0.01(0~0.02) | 1.01(0.79~1.35) | -0.19(-0.2~-0.18) | 1.03(0.86~1.23) | 0.52(0.5~0.54) | 0.04(0.03~0.04) | 0(-0.01~0) | 0.01(0~0.01) | -0.78(-0.81~-0.76) | 0.01(0~0.02) |
| Southern Sub-Saharan Africa | **0.07(0.06~0.08)** | **1.28(1.15~1.42)** | **0(-0.01~0)** | **1.18(1.1~1.3)** | **0.1(0.09~0.11)** | **0.04(0.04~0.05)** | **0.04(0.04~0.05)** | **0.01(0.01~0.02)** | **0.14(0.13~0.15)** | **0.07(0.06~0.08)** |
| Tropical Latin America | 0.03(0.02~0.03) | 0.77(0.5~1) | -0.01(-0.02~-0.01) | 1.45(1.31~1.59) | 0.45(0.44~0.47) | 0.02(0.02~0.02) | 0.01(-0.03~0.06) | -0.03(-0.04~-0.03) | -0.79(-0.8~-0.78) | 0.03(0.02~0.03) |
| Western Europe | **0.01(0~0.02)** | **0.78(0.52~0.97)** | **0.05(0.04~0.05)** | **1.04(0.87~1.24)** | **0.38(0.37~0.38)** | **0.01(0.01~0.02)** | **0.1(0.09~0.11)** | **-0.01(-0.01~0)** | **-0.93(-0.96~-0.9)** | **0.01(0~0.02)** |
| Western Sub-Saharan Africa | 0.04(0.04~0.05) | 0.23(0.12~0.35) | 0.02(0.02~0.03) | 0.41(0.33~0.52) | 0.3(0.29~0.31) | 0.07(0.07~0.07) | 0.09(0.09~0.1) | 0.06(0.06~0.06) | -0.19(-0.21~-0.17) | 0.04(0.04~0.05) |

DALYs, disability-adjusted life years; CI, confidence interval; SDI, socio-demographic index.
